# Accounting for nonsampling error in estimates of HIV epidemic trends from antenatal clinic sentinel surveillance

**DOI:** 10.1097/QAD.0000000000001419

**Published:** 2017-04

**Authors:** Jeffrey W. Eaton, Le Bao

**Affiliations:** a Department of Infectious Disease Epidemiology, Imperial College London, London, UK; b Department of Statistics, the Pennsylvania State University, University Park, Pennsylvania, USA

**Keywords:** ANC sentinel surveillance, EPP model, HIV epidemic trends, mathematical model, statistical uncertainty

## Abstract

**Objectives:**

The aim of the study was to propose and demonstrate an approach to allow additional nonsampling uncertainty about HIV prevalence measured at antenatal clinic sentinel surveillance (ANC-SS) in model-based inferences about trends in HIV incidence and prevalence.

**Design:**

Mathematical model fitted to surveillance data with Bayesian inference.

**Methods:**

We introduce a variance inflation parameter $\sigma_{in\;fl}^{2}$ that accounts for the uncertainty of nonsampling errors in ANC-SS prevalence. It is additive to the sampling error variance. Three approaches are tested for estimating $\sigma_{in\;fl}^{2}$ using ANC-SS and household survey data from 40 subnational regions in nine countries in sub-Saharan, as defined in UNAIDS 2016 estimates. Methods were compared using in-sample fit and out-of-sample prediction of ANC-SS data, fit to household survey prevalence data, and the computational implications.

**Results:**

Introducing the additional variance parameter $\sigma_{in\;fl}^{2}$ increased the error variance around ANC-SS prevalence observations by a median of 2.7 times (interquartile range 1.9–3.8). Using only sampling error in ANC-SS prevalence ( $\sigma_{in\;fl}^{2}=0$), coverage of 95% prediction intervals was 69% in out-of-sample prediction tests. This increased to 90% after introducing the additional variance parameter $\sigma_{in\;fl}^{2}$. The revised probabilistic model improved model fit to household survey prevalence and increased epidemic uncertainty intervals most during the early epidemic period before 2005. Estimating $\sigma_{in\;fl}^{2}$ did not increase the computational cost of model fitting.

**Conclusions:** We recommend estimating nonsampling error in ANC-SS as an additional parameter in Bayesian inference using the Estimation and Projection Package model. This approach may prove useful for incorporating other data sources such as routine prevalence from Prevention of mother-to-child transmission testing into future epidemic estimates.

## Background

The primary data for estimating HIV epidemic trends in sub-Saharan Africa are sentinel surveillance of HIV prevalence among pregnant women attending antenatal clinics (ANC). Since the early 1990s, HIV prevalence was measured among women attending a selection of ANC sentinel sites every 1–3 years, furnishing a time series of HIV prevalence observations in each clinic. Additional clinics have been added over time. Estimates of HIV prevalence and incidence trends are created by statistically fitting the Estimation and Projection Package (EPP) model [1,2], a simple ‘susceptible–infected’ HIV epidemic model, to ANC sentinel surveillance (ANC-SS) prevalence and prevalence from nationally representative household surveys in a Bayesian framework. A linear mixed-effects model is used to account for the potentially unbalanced repeated observations at the same sites when inferring a population prevalence trend from ANC-SS [3]. One assumption underpinning this estimation is the discrepancies between the model predictions, and the observed ANC-SS prevalence is explained by the random sampling error expected based on the sample sizes in each clinic [3].

In practice, we observe greater variation in year-to-year prevalence in time series from individual clinics than would be expected from random sampling error alone, given the sample sizes in each clinic. For example, Brown [5] noted that the in-sample coverage of clinic-level 95% prediction intervals is on average around only 88% (instead of the theoretical 95%). A number of factors could potentially contribute to the underestimation of the uncertainty, such as uncertain sampling procedures or changes in sampling procedures; changes in inclusion criteria for women attending participating facilities; poor quality control in laboratories, contamination, or changes in diagnostic tests over time; or heterogeneous variation in local epidemic trends not captured by the site-level random-effects intercepts.

Although national survey prevalence is included in the likelihood for fitting the model, in settings with large numbers of ANC sites and frequent sentinel surveys, ANC prevalence data tend to overwhelm the higher-quality national survey data, resulting in epidemic trends that can be inconsistent with national surveys. At present, there is a transition from using sentinel surveillance of HIV prevalence among a fixed number of pregnant women at a selection of ANC sites to utilizing prevalence estimated via routine HIV testing of all pregnant women attending ANC. For these data, the sampling variation of the observed prevalence is negligible due to the large sample sizes, but the nonsampling error may be expected to be even larger than for sentinel surveys [4].

In this study, we propose an additional variance parameter to allow nonsampling error in ANC prevalence observations, compare different methods for estimating the additional parameter, and examine the implications of this for estimates of HIV prevalence and incidence from the EPP model.

## Methods

### Estimation and Projection Package model

Estimation and Projection Package model is a simple susceptible-infected epidemic model that is used to infer internally consistent estimates of adult HIV prevalence and incidence trends from ANC-SS prevalence and household survey prevalence. The model stratifies the adult (age 15–49 years old) population according to susceptible individuals, CD4^+^ stage of infection, and the antiretroviral therapy (ART) population.

Further details of the EPP model are available from Brown *et al.* [2]. EPP implements two flexible models for the transmission rate *r*(*t*). The ‘r-trend’ model is a flexible seven-parameter model [5], and the ‘r-spline’ model uses penalized B-splines with seven knots, a smoothing penalty, and initial seed incidence rate (nine parameters total) [6]. In the EPP software, the r-spline model implements an equilibrium prior assumption for *r*(*t*) after the end of the data [7]. In this analysis, we examine the r-spline model both with and without the equilibrium prior assumption.

### Incorporating nonsampling error

The likelihood function currently used for fitting the EPP model to ANC-SS data assumes that all uncertainty about ANC prevalence *γ_st_* for a clinic *s* in year *t* is captured by the binomial sampling variation associated with the sample size *N_st_*, that is, the number of HIV-positive women *Y_st_* ~ Binomial (*N_st_*,*γ_st_*). Repeated observations at the same clinics over time are accounted for via a hierarchical linear model. The observed prevalence at clinic *s* in year *t* is modeled on probit scale [3]. Following notation of Alkema, Raftery, and Clark, define $\Phi^{-1}\left(\frac{Y_{st}+0.5}{N_{st}+1}\right)$, (where *Φ*^−1^ is the inverse of the normal cumulative distribution function), and the model is

$$W_{st}=\Phi^{-1}(\rho_{t})+\alpha_{\mathrm{ANC}}+b_{s}+\varepsilon_{st}\;b_{s}\sim N(0,\sigma^{2})\;\varepsilon_{st}\sim N(0,{\hat{\nu }}_{st})$$

where *ρ_t_* is the prevalence in year *t* predicted by the model, *α*_ANC_ is the systematic bias between prevalence among the ANC population compared to the general population, *b_s_* is a site-level random effect, and ∈*_st_* is the residual error. The variance of ∈*_st_* is approximated by:

$${\hat{v}}_{st}=2\pi \text{exp}\{\Phi^{-1}{\left({\hat{\gamma }}_{st}\right)}^{2}\}{\hat{\gamma }}_{st}(1-{\hat{\gamma }}_{st})/N_{st},$$

where ${\hat{\gamma }}_{st}=\frac{Y_{st}+0.5}{N_{st}+1}$ is substituted for the true prevalence *γ_st_* in the delta method approximation to var(*W_st_*). As the sample size *N_st_* increases, the variance of prevalence at the probit scale declines to zero at the rate of 1/*N_st_*.

To allow additional nonsampling error in observed ANC prevalence, we propose adding an additional variance term $\sigma_{in\;fl}^{2}$ to inflate the variance associated with clinic-level ANC prevalence observations. The residual variance becomes

$$\varepsilon_{st}\sim N(0,{\hat{v}}_{st}+\sigma_{in\;fl}^{2}).$$

The above approach maintains the relationship that uncertainty about ANC prevalence observations is related in magnitude to the expected binomial sample error, but allows additional variance in ANC prevalence observations as suggested by the data.

The additional variance could be global, with a single value fixed or estimated across countries and regions, or different values could be chosen for each setting. Consistent with the paradigm for application of the EPP model, we estimate $\sigma_{in\;fl}^{2}$ independently for each subnational region to which EPP is applied.

### Estimating $\sigma_{in\;fl}^{2}$

We consider the following three different approaches for estimating $\sigma_{in\;fl}^{2}$:

1. The simplest implementation applies a linear regression model to the residuals obtained from the original EPP model. It leads to *a priori* estimation of $\sigma_{in\;fl}^{2}$, which is then a fixed input parameter for the model fitting process,
2. Johnson *et al.* [8] and Ševčíková *et al.* [9] propose substituting an unbiased sample variance estimator for $\sigma_{in\;fl}^{2}$ in the likelihood calculation, or
3. The full Bayesian approach estimates the joint posterior distribution of $\sigma_{in\;fl}^{2}$ and other unknown parameters simultaneously.

For (1), we fit a linear regression to the probit-transformed observed ANC prevalences *W_st_* with dummy indicator variables for each clinic *s* and time *t*, analogous to the hierarchical ANC likelihood, but with fixed effects for each clinic and year. Assume the residuals of transformed ANC prevalence are independent and identically distributed with an equal variance *σ*^2^, and let *σ̂*^2^ be its maximum likelihood estimate (MLE). We estimate the excess variance by subtracting the expected sampling variation *v̂_st_*:

$${\tilde{\sigma }}_{in\;fl}^{2}=\frac{1}{\sum_{s}T_{s}}\sum_{s}\sum_{t}{\hat{\sigma }}^{2}-{\hat{v}}_{st}.$$

The estimate may underestimate the true ${\tilde{\sigma }}_{in\;fl}^{2}$ since the survey year fixed effects are completely independent, potentially allowing greater year-to-year change in prevalence rather than being constrained by the epidemiologic model. If the estimate ${\tilde{\sigma }}_{in\;fl}^{2}<0$, we set ${\tilde{\sigma }}_{in\;fl}^{2}=0$.

Second, Johnson *et al.* [8] and Ševčíková *et al.* [9] propose substituting an MLE conditional on the model output to approximate an unknown variance in the likelihood calculation. For (2), let *ρ_t_*(*θ*) be the predicted prevalence among pregnant women for a vector of input parameters *θ*. The clinic level random effect *b_s_* is approximated by

$${\hat{b}}_{s}=\frac{1}{T_{s}}\sum_{t}\left(W_{st}-\Phi^{-1}(\rho_{t}(\theta ))-\alpha_{\mathrm{ANC}}\right),$$

where T_s is the number of observations for site s, and *γ̂_st_ = Φ*^−1^(*ρ_t_*(*θ*)) + *α_ANC_* + *b̂_s_* the model predicted transformed prevalence for clinic *s* at time *t*. In the likelihood calculation we substitute

$${\hat{\sigma }}_{in\;fl}^{2}=\frac{1}{\sum T_{s}-S}\sum_{s}\sum_{t}\left\{{\left(W_{st}-{\hat{\gamma }}_{st}\right)}^{2}-{\hat{v}}_{st}\right\}$$

We subtract the number of sites *S* in the denominator to account for the lost degrees of freedom for calculating *b̂_s_* at each site.

Finally, approach (3) is to explicitly estimate $\sigma_{in\;fl}^{2}$ as an additional parameter in Bayesian inference. We used an exponential prior distribution with rate *v*_0_*=*0.015^−1^, informed by the regression analysis for approach (1). We test the sensitivity to the prior using more diffuse priors with rate *v*_0_∈{0.1^−1^,1}.

### Analysis

Data were taken from national estimates country files provided by UNAIDS for the 2016 UNAIDS estimates. We used data from nine countries in southern and eastern Africa with two or more prevalence estimates from household-based surveys: Botswana, Kenya, Lesotho, Malawi, Tanzania, Uganda, South Africa, Zambia, and Zimbabwe. For Botswana, Lesotho, Tanzania, Uganda, and Zambia models were fit separately to data from urban and rural regions. Data from Malawi were stratified by region (northern/central/southern), Kenya by eight former provinces, and South Africa and Zimbabwe by province (9 and 10, respectively), for a total of 40 subnational regions as defined for the 2016 UNAIDS estimates.

Each of the three approaches for incorporating $\sigma_{in\;fl}^{2}$ are implemented for the EPP r-trend model [5], the EPP r-spline model [7], and the EPP r-spline model without the equilibrium prior. Outcomes are compared to results without additional variance for the ANC prevalence ( $\sigma_{in\;fl}^{2}=0$).

Parameter estimation is conducted via Incremental Mixture Importance Sampling (IMIS) [10] with *B*_0_*=*100 000 initial samples and *B=*10 000 samples per iteration. R code for reproducing the analyes is available from https://github.com/jeffeaton/anc-over-dispersion.

Model outputs compared are HIV prevalence, HIV incidence, and the transmission rate *r*(*t*) for the age 15–49 population represented by EPP.

### Model selection and validation

We compare the model performance using a number of metrics: such as the coverage of the predictive interval, the log-posterior predictive density (LPPD) [11], the computing cost, and coefficient of variation at different time points. We obtain the posterior predictive distribution for each ANC observation and calculate the coverage of 95%predictive intervals (in-sample fit).We also conduct an out-of-sample validation by fitting the model to 90% of the ANC observations as a training dataset while ensuring at least one observation is retained for each ANC site, and withholding the remaining 10% as a test dataset. Fifty training/test splits are created randomly for each dataset and the coverages of the 95%predictive intervals are calculated by using the observations from test datasets. Second, we evaluate the effect of including $\sigma_{in\;fl}^{2}$ for model fit to household survey HIV prevalence by calculating the LPPD, a measure of model accuracy in Bayesian framework [11]. The computational implications of incorporating $\sigma_{in\;fl}^{2}$ were assessed by comparing the median number and interquartile range (IQR) of iterations for convergence of the IMIS algorithm. Finally, we summarize the overall uncertainty about the epidemic by calculating the coefficient of variation for estimated HIV prevalence and HIV incidence at different time points in the epidemic.

## Results

### Derivation of prior

The first estimation approach does not require refitting the EPP models to the surveillance data, and provides a reasonable initial approximation to the value of the additional variance parameter. Following that approach, regression-based estimates of ${\tilde{\sigma }}_{in\;fl}^{2}$ for the 40 datasets considered range from 0 to 0.030, with mean of 0.007 and median 0.003. Based on this, we propose an exponential distribution with rate 0.015^−1^ as an informative prior distribution for the full Bayesian approach. This distribution has a median of 0.01 and 90% of the mass less than 0.035.

### Estimates for $\sigma_{in\;fl}^{2}$

**Fig. 1:**
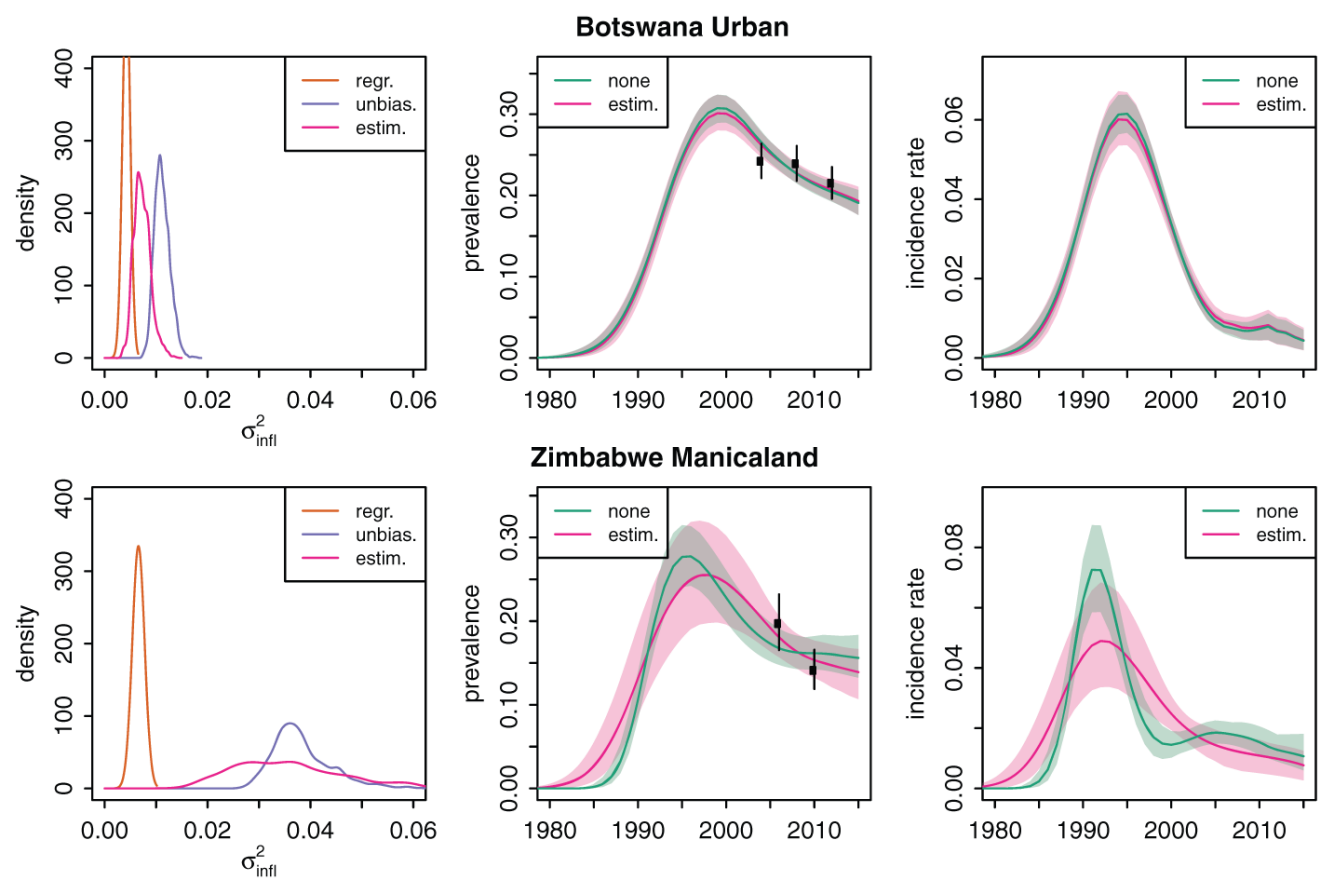
Example of effect of accounting for nonsampling error in ANC-SS prevalence for Botswana Urban and Zimbabwe Manicaland regions Left panels illustrate estimates for $\sigma_{in\;fl}^{2}$ by different approaches. Center panels show the effect on estimates of adult HIV prevalence when $\sigma_{in\;fl}^{2}$ is estimated in full Bayesian inference (pink) versus not included ( $\sigma_{in\;fl}^{2}=0$; green). Right panels illustrate the estimated trend in HIV incidence. Shaded regions represent 95% credible intervals. Results for other approaches and regions are provided in Supplementary Appendix (http://links.lww.com/QAD/B45). ANC-SS, antenatal clinic sentinel surveillance.

Table 1 summarizes the estimates of $\sigma_{in\;fl}^{2}$ by different approaches for the 40 datasets, and the left panels of Fig. 1 shows the posterior density estimates for the three estimation approaches. Estimates for all sites are shown in supplementary figures (http://links.lww.com/QAD/B45). For each estimation approach, estimated values for $\sigma_{in\;fl}^{2}$ were similar across the three EPP model variants r-trend, r-spline with equilibrium prior, and r-spline without equilibrium prior. The first estimation approach based on regression output yielded smaller values (median 0.003, IQR 0.0–0.007) than the two alternatives, suggesting that the regression estimator underestimated the extra variance. The point estimates between the unbiased variance estimator and the full Bayesian estimator were highly correlated across the 40 datasets (correlation*=*0.91).

By comparison, the mean value for *v̂_st_* in each dataset, the transformed sampling variance around observed ANC prevalence, has a median of 0.008 (IQR 0.006–0.014). Estimating $\sigma_{in\;fl}^{2}$ with the r-spline model increased the error variance about ANC prevalence by 2.7 times (IQR 1.9–3.8), measured by ( $\sigma_{in\;fl}^{2}+{\hat{v}}_{st}/{\hat{v}}_{st}$).

Analysis of variance (ANOVA) shows that estimates of $\sigma_{in\;fl}^{2}$ for regions within the same country were more similar than values of $\sigma_{in\;fl}^{2}$ between countries (*P*<0.001), with country explaining 56% of the variation in the log of the mean value of $\sigma_{in\;fl}^{2}$. The number of regions into which a country was stratified, urban versus rural location, the number of sentinel sites, and the median number of observations per site were not associated with the estimated value of $\sigma_{in\;fl}^{2}$.

### Comparing model fit

To choose a recommended approach, we compared the approaches in terms of how well they fit the ANC data in in-sample comparisons, out-of-sample prediction, fit to household survey prevalence data, and the computational implications (Table 2). Fitting the r-trend model, with sampling variance only ( $\sigma_{in\;fl}^{2}=0$) the average coverage of 95% prediction intervals was 78%. With the fixed regression estimator, this increased to 86%, and substituting the unbiased variance estimator or explicitly estimating $\sigma_{in\;fl}^{2}$ in the Bayesian model achieved very close to the theoretical 95% coverage. In out-of-sample prediction, when $\sigma_{in\;fl}^{2}=0$, the coverage was 69%. This increased to 90 and 89%, respectively, when using the unbiased variance estimator or jointly estimating $\sigma_{in\;fl}^{2}$. Similar coverage results were observed for r-spline models with and without equilibrium prior.

Incorporating $\sigma_{in\;fl}^{2}$ improved the fit to household survey prevalence data, measured by the log posterior predictive density (LPPD). This is expected because the effect of additional variance for ANC data is to give relatively less weight to ANC data in the likelihood, and hence more weight to household survey prevalence.

Finally, we used the number of iterations of the IMIS algorithm, which scales with the number of likelihood evaluations required, as a proxy for the computational implications of the candidate approaches for incorporating $\sigma_{in\;fl}^{2}$. The bottom rows of Table 2 show the median number of IMIS iterations required for convergence across 50 fits for the 40 regions in out-of-sample prediction simulations, and the width of the IQR as a measure of the variability in model fitting. The median number of iterations was twice as many for the r-spline model than the r-trend model (55 for r-spline, 56 without the equilibrium prior, 27 for r-trend). None of the approaches systematically affected the number of iterations required for convergence, including explicit estimation of an additional model parameter. They also did not increase the variation in number of iterations for model fitting.

### Implications for HIV estimates

Figure 1 illustrates the trend for HIV prevalence (second column) and incidence (third column) estimated by the r-spline model without additional variance ( $\sigma_{in\;fl}^{2}=0$; green lines) and when $\sigma_{in\;fl}^{2}$ is estimated as an additional parameter in Bayes (pink lines) for two EPP regions: Botswana Urban and Zimbabwe Manicaland. For Botswana Urban, the posterior mean of $\sigma_{in\;fl}^{2}$ was 0.007 [95% confidence interval (CI) 0.005–0.011], and estimates for incidence and prevalence were relatively similar with slightly greater epidemic uncertainty when allowing nonsampling error. In contrast, for Zimbabwe Manicaland, the estimates of $\sigma_{in\;fl}^{2}$ were larger (mean 0.036; 95% CI 0.019–0.060), and the inferred epidemic trend peaks and declines less rapidly and is more consistent with the prevalence trend observed in national household surveys. Uncertainty is much wider.

Allowing nonsampling error in ANC prevalence systematically increases the uncertainty about epidemic estimates (Table 3). The magnitude of the increase in uncertainty depends on the timing of the epidemic and the model choice. Using the r-trend model, the median increase in the coefficient of variation about prevalence was 1.49 times in 1990, 1.32 in 1995, 1.25 in 2000, and 1.04 times in 2005. For the r-spline models the increase was slightly less: 1.40 times in 1990 declining to 1.03 times in 2005. The decline over time is because during the late 2000 period, the level and uncertainty is largely determined by household survey prevalence, whereas uncertainty about the course of the epidemic from ANC-SS data has a larger effect on the estimates before the survey availability.

### Sensitivity to prior

**Fig. 2:**
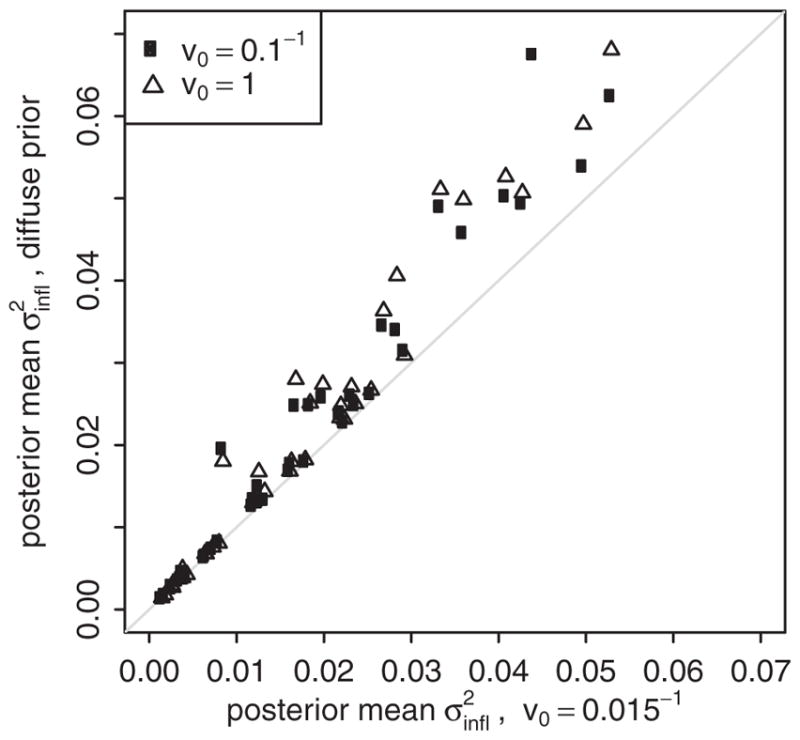
Sensitivity of estimates $\sigma_{\mathrm{infl}}^{2}$ to the prior distribution Scatter plot illustrates the posterior mean estimate for $\sigma_{\mathrm{infl}}^{2}$ using the informative prior with rate *v*_0_*=*0.015^−1^ compared to the posterior mean with more diffuse priors (*v*_0_*=*0.1^−1^: circles; *v*_0_*=*1: triangles). Points represent estimates from the same dataset. Results are presented for the r-spline model.

Figure 2 presents the posterior mean for $\sigma_{in\;fl}^{2}$ using the informative prior with rate *v*_0_*=*0.015^−1^ (horizontal axis) compared to more diffuse priors with *v*_0_*=*0.1^−1^ (dots) and *v*_0_*=*1 (triangles). At small values of $\sigma_{in\;fl}^{2}$ the estimates are very similar, whereas at larger values of $\sigma_{in\;fl}^{2}$ there is some evidence of attenuation using the informative prior. Results in Fig. 2 are based the r-spline model, and are similar for the other model variants.

## Discussion

Probabilistic uncertainty for HIV epidemic estimates was a major innovation for the robustness of HIV estimates and projections from HIV surveillance data [12]. The imperfect statistical fit of the statistical model for ANC-SS has noted previously [5], potentially giving too much weight to outliers and overemphasizing ANC-SS data relative to other data sources. In this study, we propose a method to account for additional variance and demonstrate that this improves the statistical fit to data from both ANC sentinel surveillance and from national household-based prevalence surveys, which generally have more rigorous sampling.

We recommended estimating the additional variance $\sigma_{in\;fl}^{2}$ as an additional parameter in the full Bayesian inference because this captures the full uncertainty about the error variance without incurring additional computational cost. Although the number of parameters has increased, the added parameter flattens the sharp peaks in the likelihood, which are likely caused by underestimates of the residual variance of ANC-SS data. Estimating $\sigma_{in\;fl}^{2}$ as an additional parameter performed similarly to substituting an unbiased estimator for the variance in the likelihood calculation, an approach that has been used elsewhere [8,9]. That values of $\sigma_{in\;fl}^{2}$ were more similar for regions within the same country suggest that greater nonsampling error may reflect changes over time in the methodology or quality of the national sentinel surveillance systems.

Accounting for nonsampling error systematically and appropriately increases the uncertainty about HIV prevalence and incidence during early periods of the epidemic. This additional variance allows for greater uncertainty than would be expected relative to plausible epidemic trends represented by the EPP model. Some error could also be attributable to ‘model error’ if the model is not flexible enough to capture the true underlying epidemic patterns. This highlights a key challenge for further methodological enhancements to improve model-based HIV epidemic estimates: the need for model structure to constrain epidemiologically plausible estimates during the early epidemic period when HIV data were very sparse, but the desire for a suitably flexible model that is sensitive to recent changes in epidemic trends which are of greatest policy interest.

The additional uncertainty does not overcome other fundamental assumptions that underpin the interpretation of ANC-SS data for estimating population-wide epidemic trends, including potential selection biases arising from initial sentinel sites having been selected as a ‘convenience sample’ [13], and the assumption that the epidemic trend is homogenous in all ANC sites. These may prove to be substantially greater sources of uncertainty than that discussed here. It also does not account for the true uncertainty about other model parameters which are currently treated as fixed in epidemic inference, such as disease progression and survival, mortality rates on ART, and the relationship between fertility and HIV [14,15]. Reflecting the uncertainty about these is expected to further increase probabilistic uncertainty intervals generated by the EPP model around incidence trends inferred from prevalence and is an area for future development.

In some datasets, different approaches to estimating $\sigma_{in\;fl}^{2}$ or different EPP model variants resulted in dramatically different and sometimes implausible epidemic patterns (supplementary figures, http://links.lww.com/QAD/B45). This highlights the extent to which multiple epidemic trends could be considered broadly consistent with available data, suggesting even expanded uncertainty intervals may understate the true uncertainty about the epidemic. In real applications of the EPP software to create country-level HIV estimates, users have the opportunity to specify prior constraints on HIV prevalence to exclude implausible epidemic curves in formal Bayesian Melding, but ideally these should be specified *a priori* before doing any model fitting. Hierarchically sharing information between neighboring regions constitutes a systematic approach to enhance epidemiologically plausible estimates in regions where data are sparse [16].

Fully quantifying all sources of uncertainty about data becomes increasingly important as more data sources are incorporated into estimates and each must be given appropriate weight relative to others. Examples of other data sources which could enhance future estimates of HIV epidemic trends include routine test of pregnant women attending ANC, adult mortality data, phylodynamic data, HIV incidence assays, and biomarkers for antiretroviral drug usage. The approach proposed here may be naturally applicable to the inclusion of routine prevalence data from Prevention of mother-to-child transmission testing, where the sampling error may be negligible owing to very large sample sizes, but nonsampling error may be a much greater source of uncertainty because of unknowns about the sampling frame, completeness, reporting errors, and other factors related to the ‘routine’ nature of the data [4].

## Supplementary Material

1

## Figures and Tables

**Table 1:** Summary of estimates of $\sigma_{\mathrm{infl}}^{2}$ across the 40 datasets for the three approaches.

$\sigma_{in\;fl}^{2}$ estimator	Median	Mean	IQR	Min/max
r-trend model				
Regression	0.003	0.007	(0.000, 0.007)	(0.000, 0.030)
Unbiased	0.016	0.023	(0.007, 0.030)	(0.002, 0.080)
Estimated	0.018	0.019	(0.007, 0.026)	(0.001, 0.052)
r-spline model				
Regression	0.003	0.007	(0.000, 0.007)	(0.000, 0.030)
Unbiased	0.016	0.022	(0.006, 0.028)	(0.001, 0.082)
Estimated	0.017	0.019	(0.006, 0.026)	(0.001, 0.053)
r-spline, no equilibrium prior				
Regression	0.003	0.007	(0.000, 0.007)	(0.000, 0.030)
Unbiased	0.015	0.021	(0.006, 0.027)	(0.001, 0.066)
Estimated	0.017	0.018	(0.006, 0.025)	(0.001, 0.051)
Mean sampling error (*v̂_st_*)	0.008	0.011	(0.006, 0.014)	(0.004, 0.030)
Relative increase $(\sigma_{\mathrm{infl}}^{2}+{\hat{v}}_{st})/{\hat{v}}_{st}$	2.664	2.982	(1.882, 3.809)	(1.093, 6.876)

For comparison, the bottom two rows show the summary of the average sampling variance *v̂_st_* for each dataset, and the relative increase in the variance for ANC observations when estimating $\sigma_{\mathrm{infl}}^{2}$ in full Bayesian inference. IQR, interquartile range.

**Table 2:** Effect of incorporating $\sigma_{in\;fl}^{2}$ for statistical fit to ANC-SS and household survey data with r-trend model, r-spline model, and r-spline model without equilibrium prior.

	r-trend				r-spline				r-spline, no equilibrium prior			
		
	None	Regr.	Unbia.	Estim.	None	Regr.	Unbia.	Estim.	None	Regr.	Unbia.	Estim.
In-sample ANC coverage (%)^a^	77.8	86.4	95.7	94.9	77.7	85.9	95.2	94.5	78.1	86.9	95.4	94.7
Out-of-sample prediction coverage (%)^b^	68.6	76.7	89.7	89.2	68.3	76.5	90	89.6	69.2	77.4	89.4	89.6
LPPD for HH survey prevalence^c^	2.70	2.95	3.24	3.24	2.81	3.13	3.26	3.26	2.66	2.93	3.09	3.08
Change in LPPD^d^		0.25	0.55	0.55		0.33	0.45	0.45		0.27	0.43	0.43
Median IMIS iterations^e^	25.5	26.4	28.6	26.9	52.6	53.5	52.7	55.0	56.2	55.5	53.5	56.0
IQR of IMIS iterations^f^	7.0	6.9	6.6	6.2	11.5	12.2	11.5	11.8	12.7	12.5	12.2	12.7

Results represent means over 40 regions. ‘None’*=*no additional variance ( $\sigma_{in\;fl}^{2}=0$); ‘regr’*=*regression estimator (approach 1); ‘unbia.’*=*substituting unbiased variance estimator (approach 2); ‘estim.’*=*full Bayesian inference (approach 3). ANC, antenatal clinic; HH, household; IMIS, Incremental Mixture Importance Sampling; IQR, interquartile range; LPPD, log-posterior predictive density.

a In-sample coverage of 95% posterior predictive interval for observed ANC prevalence data points that were included in model fitting.

b Coverage of 95% posterior predictive intervals for 10% of withheld ANC prevalence data points, averaged over 50 randomly created training / test data splits.

c Log posterior predictive density (LPPD) for population prevalence in household surveys.

d Change in LPPD defined as the average difference in LPPD when incorporating $\sigma_{in\;fl}^{2}$ compared to ‘none’.

e Median number of IMIS iteration in 50 model fits for out-of-sample prediction test, average over 40 datasets.

f Span of the interquartile range of the number of IMIS iterations required for the 50 out-of-sample prediction model fits, averaged over 40 datasets.

**Table 3:** Effect of estimating $\sigma_{\mathrm{infl}}^{2}$ on uncertainty about epidemic estimates of HIV prevalence and incidence.

	HIV prevalence				HIV incidence			
	
	1990	1995	2000	2005	1990	1995	2000	2005
r-trend model								
CV, no $\sigma_{\mathrm{infl}}^{2}$	0.19 [0.12–0.23]	0.09 [0.07–0.11]	0.08 [0.05–0.09]	0.06 [0.05–0.09]	0.13 [0.11–0.20]	0.11 [0.09–0.14]	0.11 [0.07–0.15]	0.12 [0.09–0.17]
CV, $\sigma_{\mathrm{infl}}^{2}$ estim.	0.26 [0.18–0.39]	0.13 [0.09–0.19]	0.10 [0.07–0.12]	0.07 [0.05–0.09]	0.25 [0.18–0.39]	0.16 [0.13–0.20]	0.13 [0.10–0.19]	0.15 [0.10–0.19]
Ratio	1.49 [1.26–1.78]	1.32 [1.22–1.68]	1.25 [1.11–1.46]	1.04 [1.01–1.12]	1.52 [1.25–1.94]	1.36 [1.20–1.73]	1.27 [1.06–1.39]	1.11 [1.01–1.31]
r-spline model								
CV, no $\sigma_{\mathrm{infl}}^{2}$	0.16 [0.12–0.21]	0.08 [0.07–0.10]	0.07 [0.06–0.08]	0.06 [0.05–0.09]	0.12 [0.11–0.16]	0.10 [0.09–0.13]	0.10 [0.08–0.14]	0.12 [0.09–0.16]
CV, $\sigma_{\mathrm{infl}}^{2}$ estim.	0.21 [0.16–0.29]	0.12 [0.09–0.17]	0.08 [0.07–0.11]	0.07 [0.05–0.09]	0.17 [0.13–0.23]	0.13 [0.09–0.18]	0.10 [0.08–0.13]	0.12 [0.09–0.18]
Ratio	1.40 [1.19–1.54]	1.33 [1.17–1.51]	1.18 [1.09–1.29]	1.03 [1.00–1.08]	1.34 [1.11–1.50]	1.21 [1.12–1.40]	1.03 [0.95–1.13]	1.10 [0.98–1.18]
r-spline, no equilibrium prior								
CV, no $\sigma_{\mathrm{infl}}^{2}$	0.16 [0.12–0.21]	0.09 [0.07–0.11]	0.07 [0.06–0.08]	0.07 [0.05–0.09]	0.12 [0.11–0.16]	0.11 [0.09–0.13]	0.10 [0.07–0.15]	0.13 [0.10–0.18]
CV, $\sigma_{\mathrm{infl}}^{2}$ estim.	0.21 [0.17–0.29]	0.13 [0.10–0.16]	0.10 [0.08–0.11]	0.07 [0.06–0.09]	0.17 [0.15–0.23]	0.15 [0.12–0.17]	0.12 [0.09–0.16]	0.15 [0.12–0.22]
Ratio	1.36 [1.10–1.60]	1.36 [1.18–1.56]	1.23 [1.09–1.35]	1.06 [1.03–1.13]	1.37 [1.15–1.54]	1.29 [1.15–1.43]	1.14 [1.01–1.27]	1.16 [1.09–1.32]

Measured via coefficient of variation (CV) in years 1990, 1995, 2000, and 2005 when estimating $\sigma_{\mathrm{infl}}^{2}$ versus not. Results are median and interquartile range (IQR) over 40 datasets. CV, coefficient of variation.

## References

[1] Stover J, Brown T, Puckett R, Peerapatanapokin W (2017). Updates to the Spectrum/Estimations and Projections Package model for estimating trends and current estimates for key HIV indicators. AIDS.

[2] Brown T, Bao L, Eaton JW, Hogan DR, Mahy M, Marsh K (2014). Improvements in prevalence trend fitting and incidence estimation in EPP 2013. AIDS.

[3] Alkema L, Raftery AE, Clark SJ (2007). Probabilistic projections of HIV prevalence using Bayesian melding. Ann Appl Stat.

[4] Sheng B, Marsh K, Slavkovic AB, Gregson S, Eaton JW, Bao L (2017). Statistical models for incorporating data from routine HIV testing of pregnant women at antenatal clinics into HIV/AIDS epidemic estimates. AIDS.

[5] Bao L (2012). A new infectious disease model for estimating and projecting HIV/AIDS epidemics. Sex Transm Infect.

[6] Hogan DR, Zaslavsky AM, Hammitt JK, Salomon JA (2010). Flexible epidemiological model for estimates and short-term projections in generalised HIV/AIDS epidemics. Sex Transm Infect.

[7] Hogan DR, Salomon JA (2012). Spline-based modelling of trends in the force of HIV infection, with application to the UNAIDS Estimation and Projection Package. Sex Transm Infect.

[8] Johnson LF, Hallett TB, Rehle TM, Dorrington RE (2012). The effect of changes in condom usage and antiretroviral treatment coverage on human immunodeficiency virus incidence in South Africa: a model-based analysis. J R Soc Interface.

[9] Ševčíková H, Raftery AE, Waddell PA (2007). Assessing uncertainty in urban simulations using Bayesian melding. Transp Res Part B Methodol.

[10] Raftery AE, Bao L (2010). Estimating and projecting trends in HIV/ AIDS generalized epidemics using incremental mixture importance sampling. Biometrics.

[11] Gelman A, Hwang J, Vehtari A (2013). Understanding predictive information criteria for Bayesian models. Stat Comput.

[12] Alkema L, Raftery AE, Brown T (2008). Bayesian melding for estimating uncertainty in national HIV prevalence estimates. Sex Transm Infect.

[13] UNAIDS/WHO Working Group on Global HIV/AIDS and STI Surveillance (2003). Guidelines for Conducting HIV Sentinel Serosurveys among Pregnant Women and Other Groups.

[14] Johnson L (2014). THEMBISA version 1.0: A model for evaluating the impact of HIV/AIDS in South Africa. Cent Infect Dis Epidemiol Res Work Pap.

[15] Wang H, Wolock TM, Carter A, Nguyen G, Kyu HH, Gakidou E, GBD 2015 HIV Collaborators (2016). Estimates of global, regional, and national incidence, prevalence, and mortality of HIV, 1980–2015: the Global Burden of Disease Study 2015. lancet HIV.

[16] Niu X, Zhang A, Brown T, Puckett R, Mahy M, Bao L (2017). Incorporation of hierarchical structure into estimation and projection package fitting with examples of estimating subnational HIV/AIDS dynamics. AIDS.

