# Supplementary material for: Accounting for nonsampling error in estimates of HIV epidemic trends from antenatal clinic sentinel surveillance
Source: AIDS. Author manuscript; Available in PMC 2017 Nov 9. (PMC5679137; doi:10.1097/QAD.0000000000001419)
Supplement: 1 [file NIHMS917413-supplement-1.pdf]

Botswana Urban

r−trend

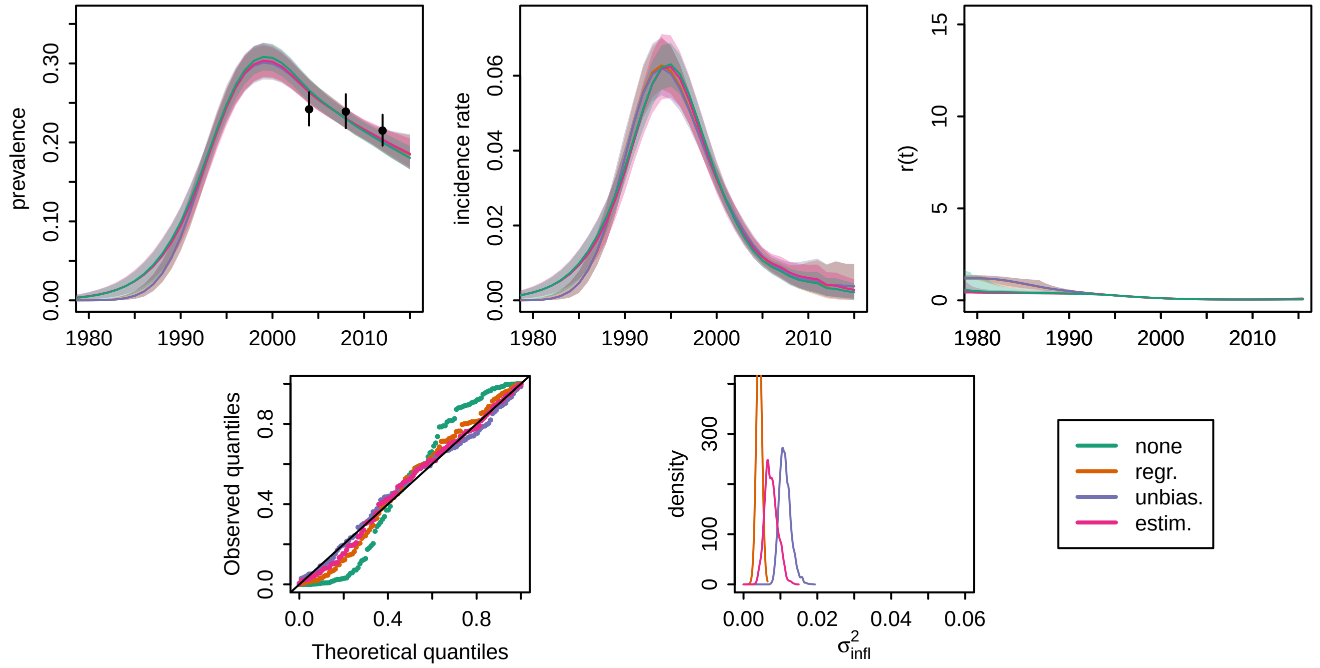

r−spline

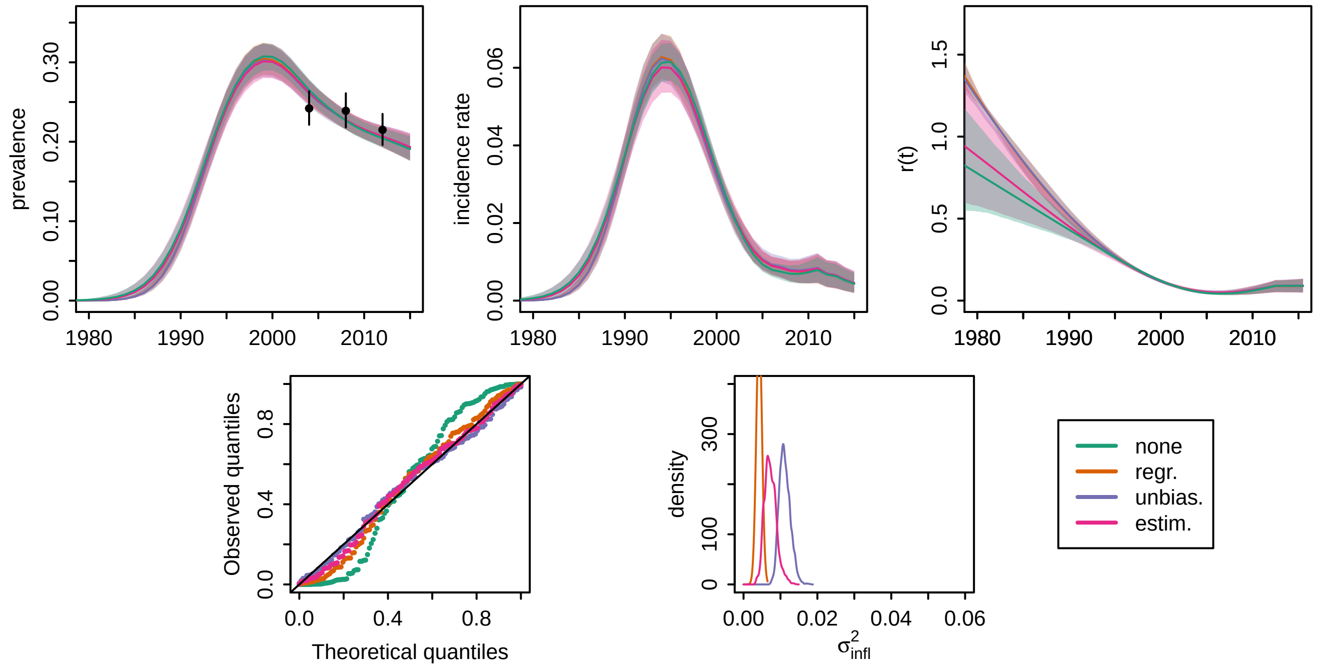

r−spline, no equil. prior

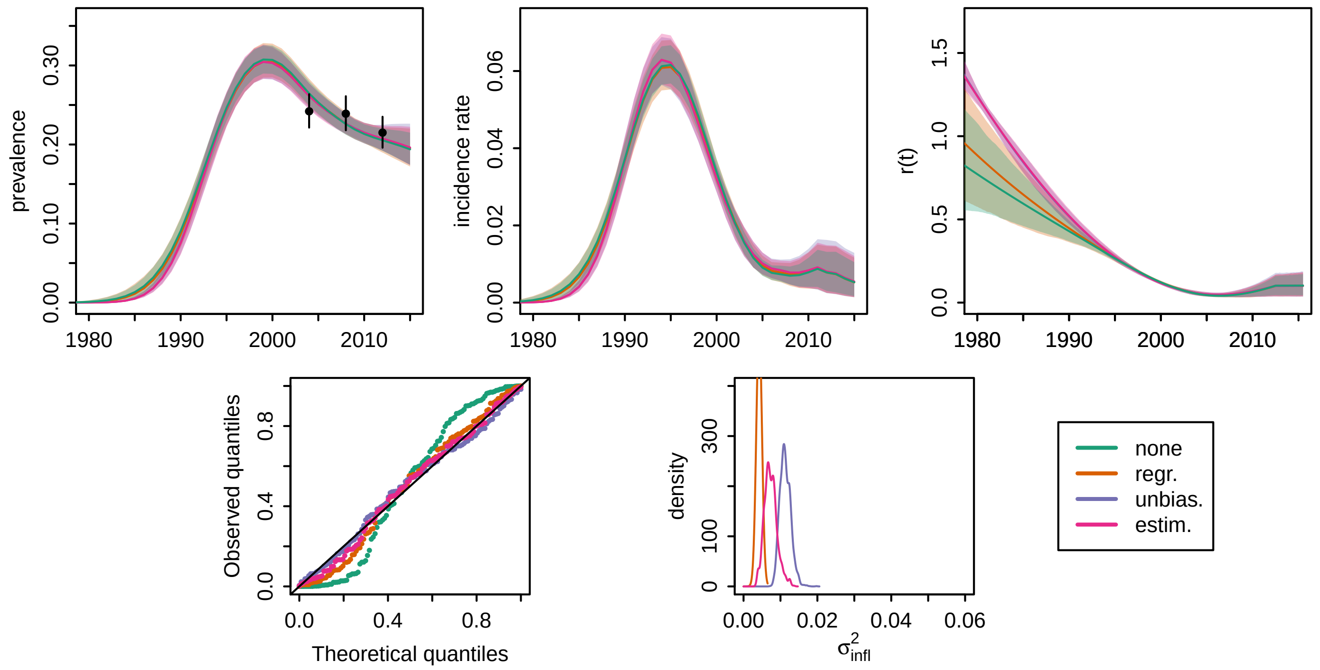

r−trend

Botswana Rural

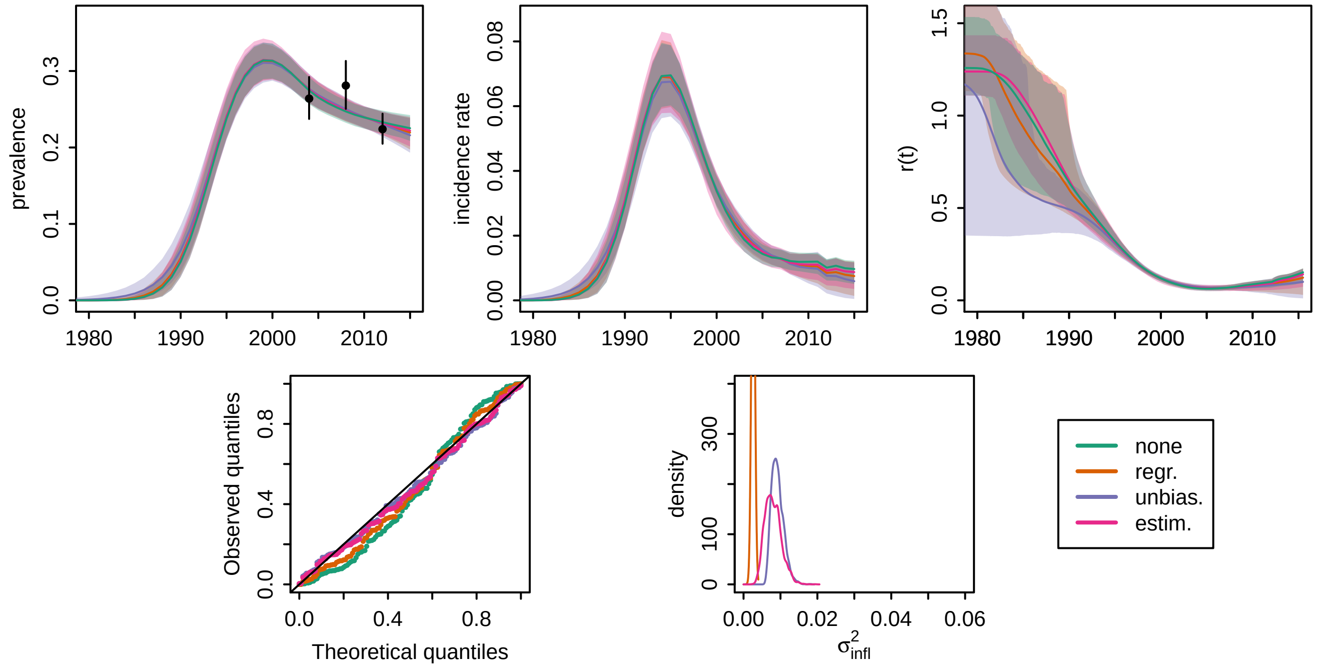

r−spline

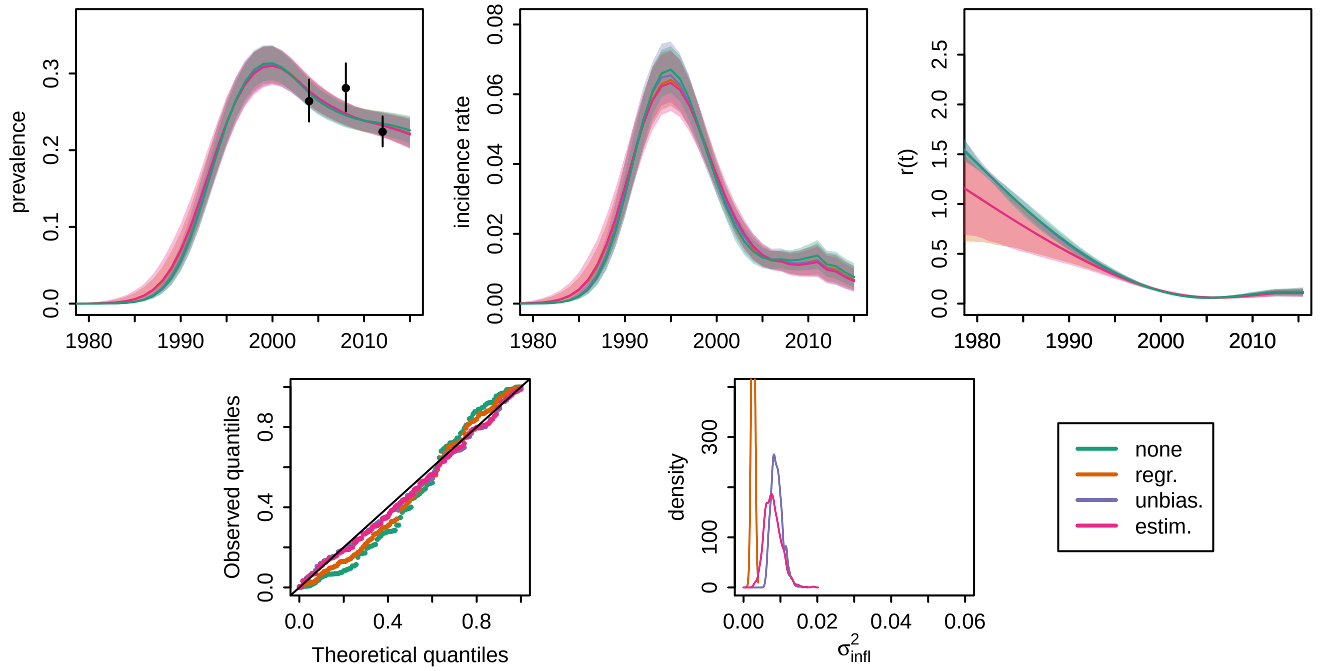

r−spline, no equil. prior

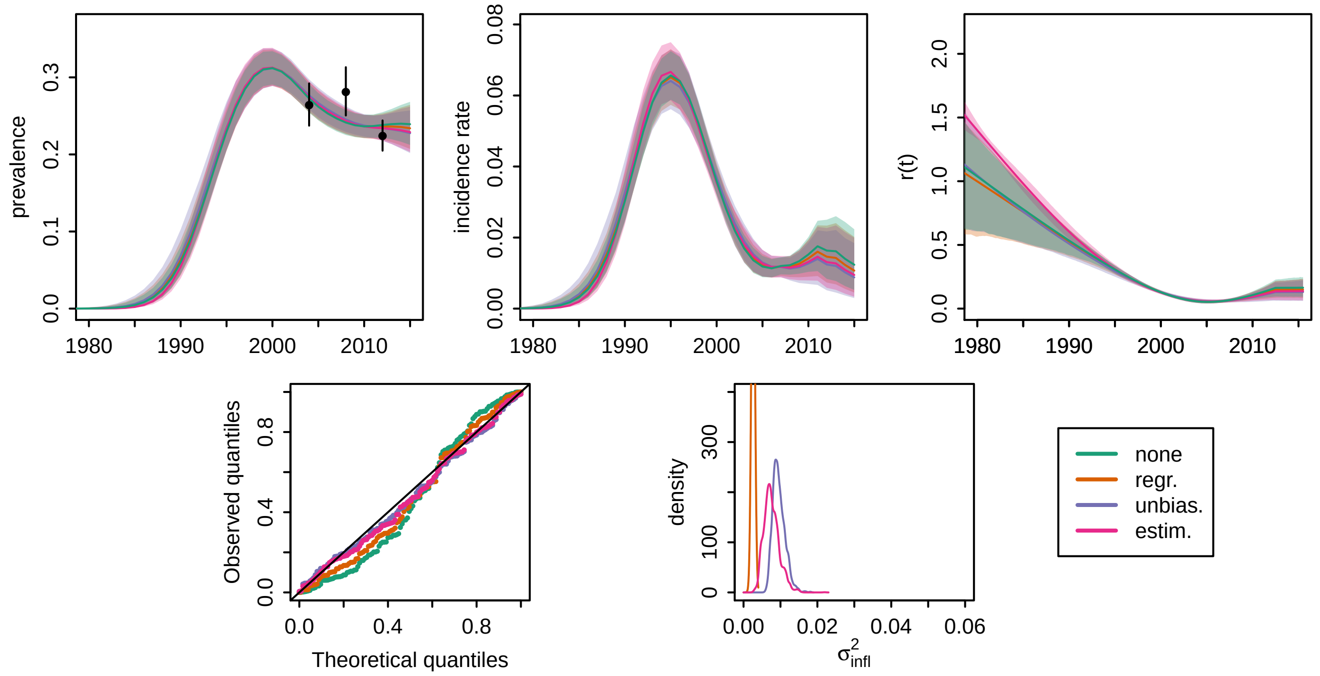

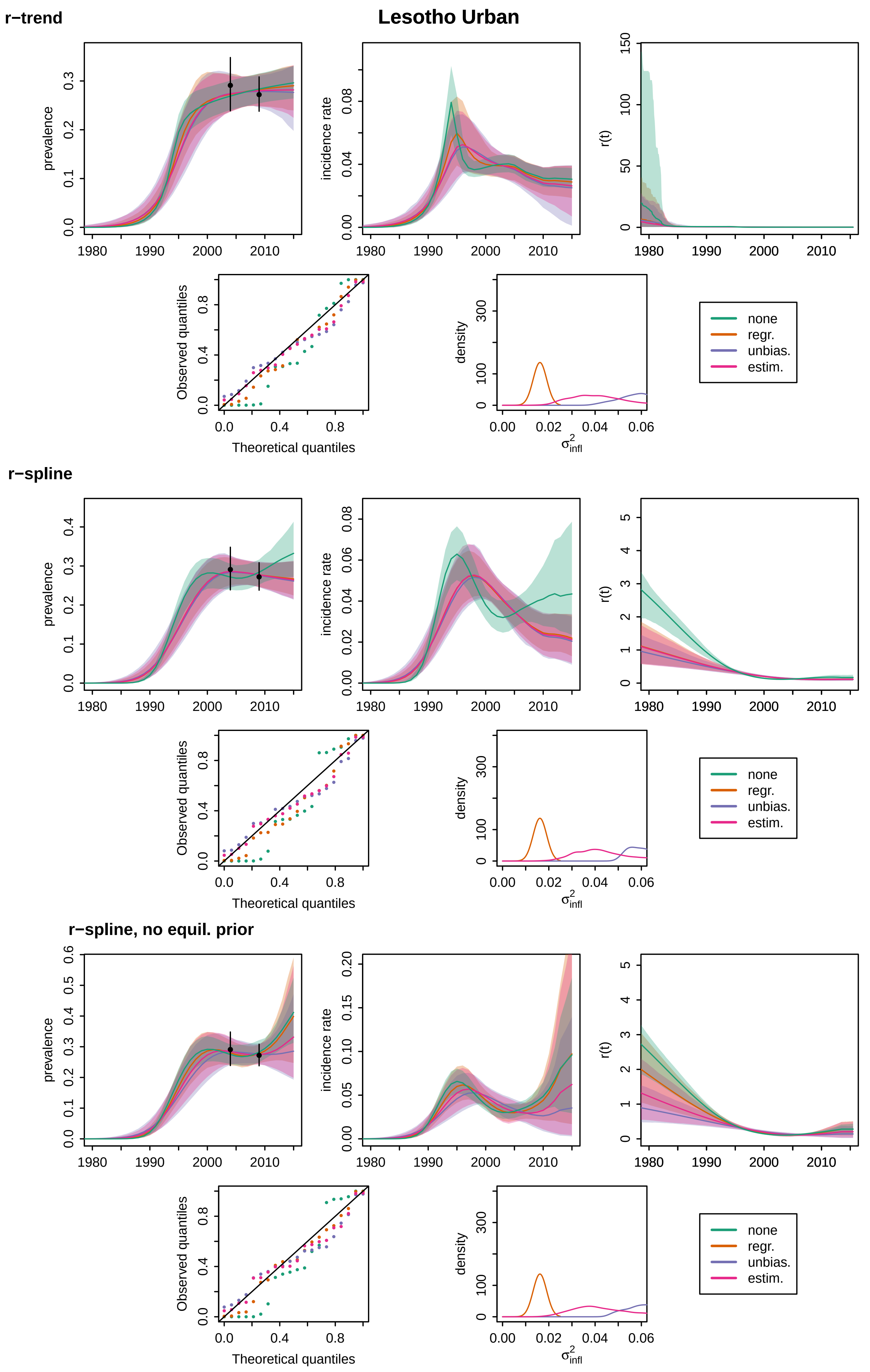

Lesotho Urban
r−trend
r−spline
r−spline, no equil. prior
prevalence
incidence rate
r(t)
Observed quantiles
Theoretical quantiles
density
σ²infl
none
regr.
unbias.
estim.

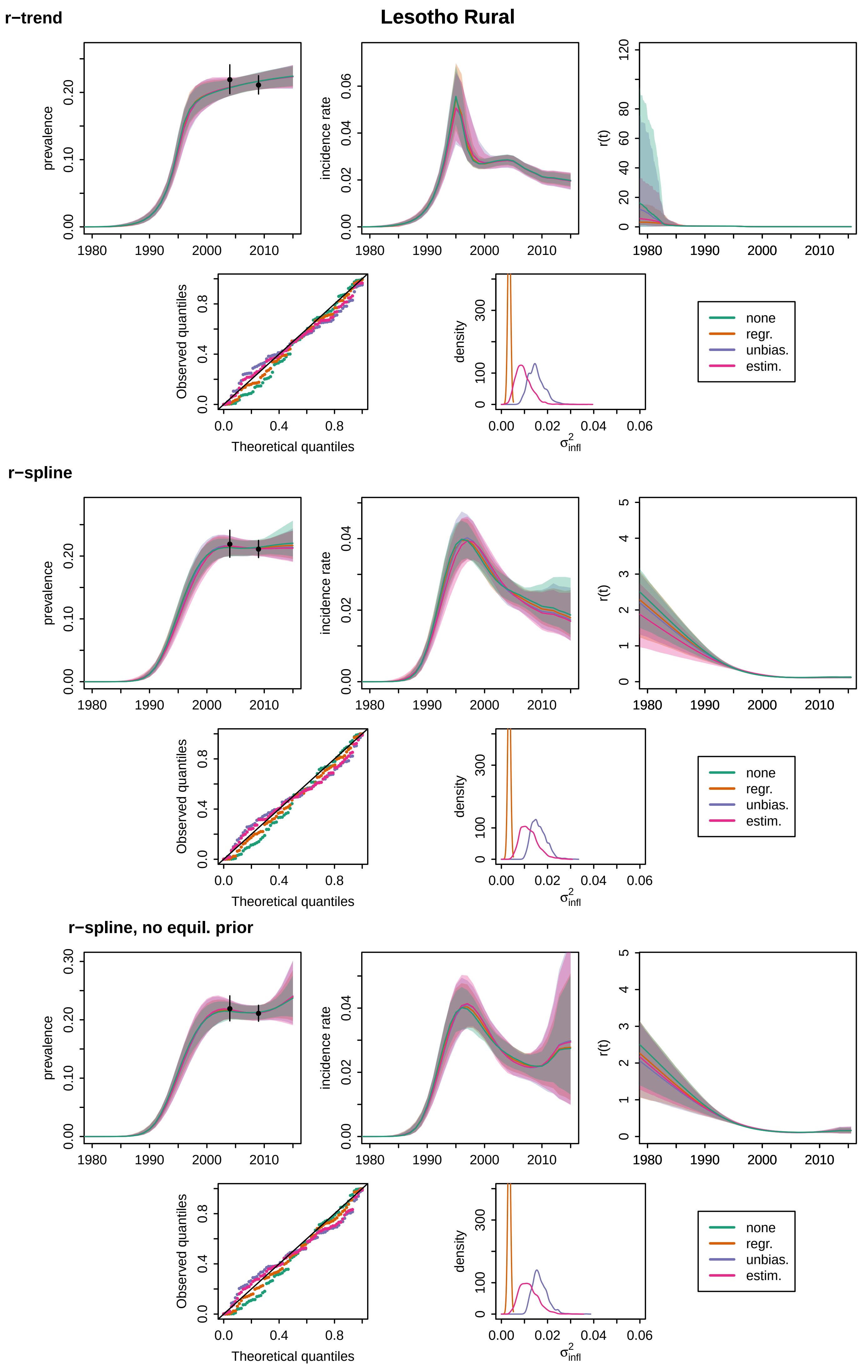
Lesotho Rural
r−trend
r−spline
r−spline, no equil. prior
prevalence
incidence rate
r(t)
Observed quantiles
Theoretical quantiles
density
σ²infl
none
regr.
unbias.
estim.

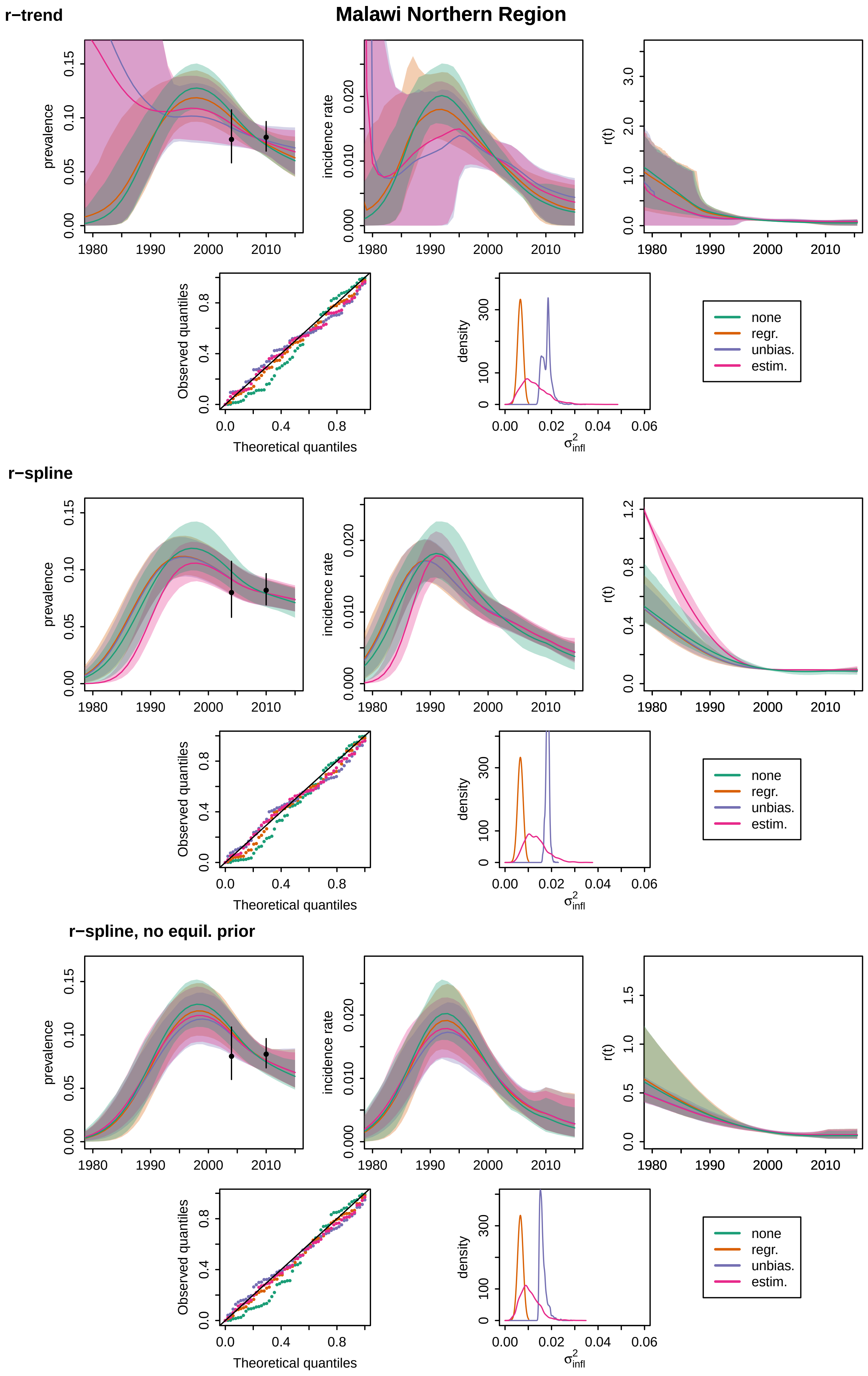
Malawi Northern Region
r−trend
r−spline
r−spline, no equil. prior
prevalence
incidence rate
r(t)
Observed quantiles
Theoretical quantiles
density
σ^2_{infl}
none
regr.
unbias.
estim.
1980
1990
2000
2010
0.00
0.05
0.10
0.15
0.000
0.010
0.020
0.0
1.0
2.0
3.0
0.4
0.8
1.2
0.5
1.5
0.02
0.04
0.06
100
300

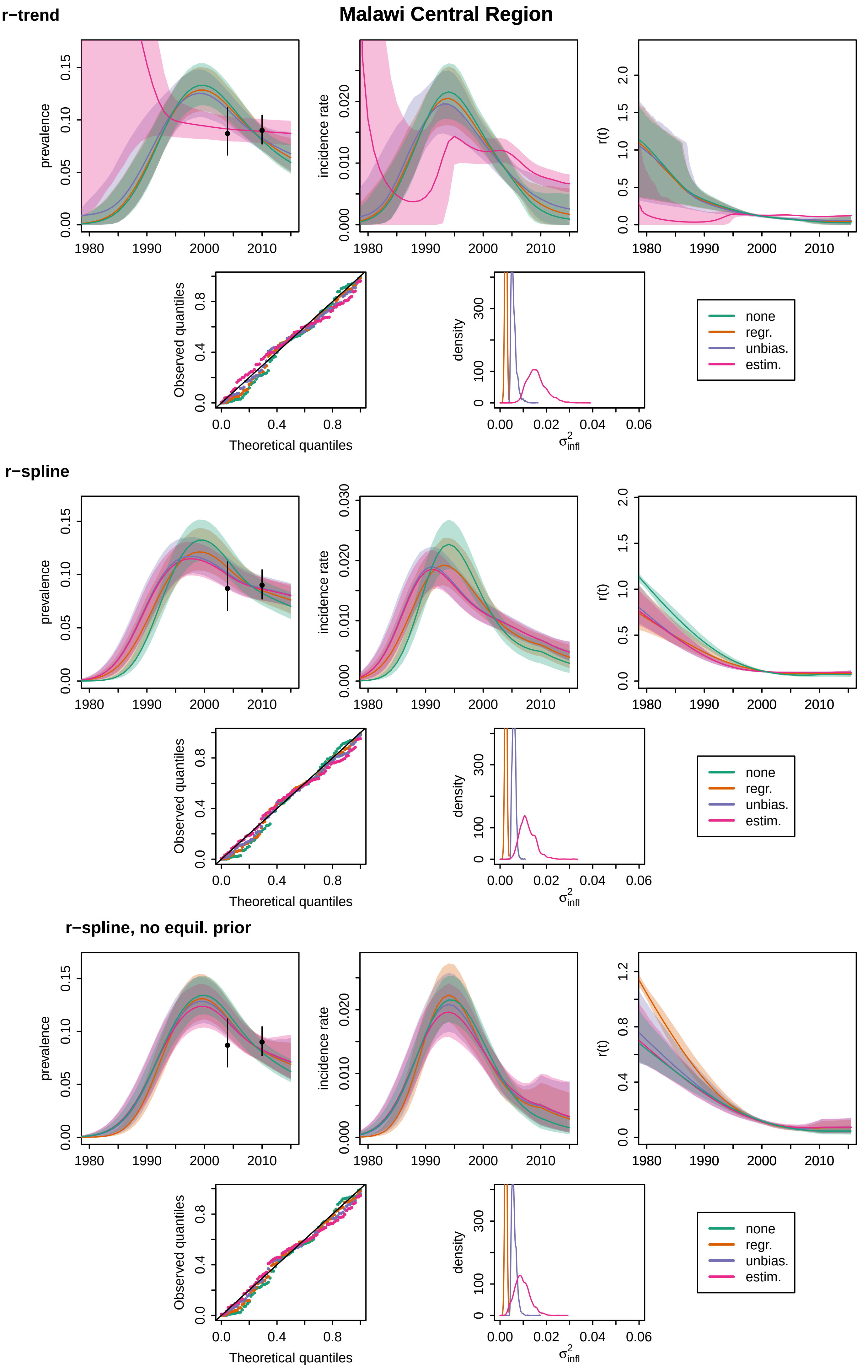
Malawi Central Region
r−trend
r−spline
r−spline, no equil. prior
prevalence
incidence rate
r(t)
Observed quantiles
Theoretical quantiles
density
σ^2_{infl}
none
regr.
unbias.
estim.
1980
1990
2000
2010
0.00
0.05
0.10
0.15
0.000
0.010
0.020
0.030
0.0
0.5
1.0
1.5
2.0
0.4
0.8
1.2
0
100
300
0.02
0.04
0.06

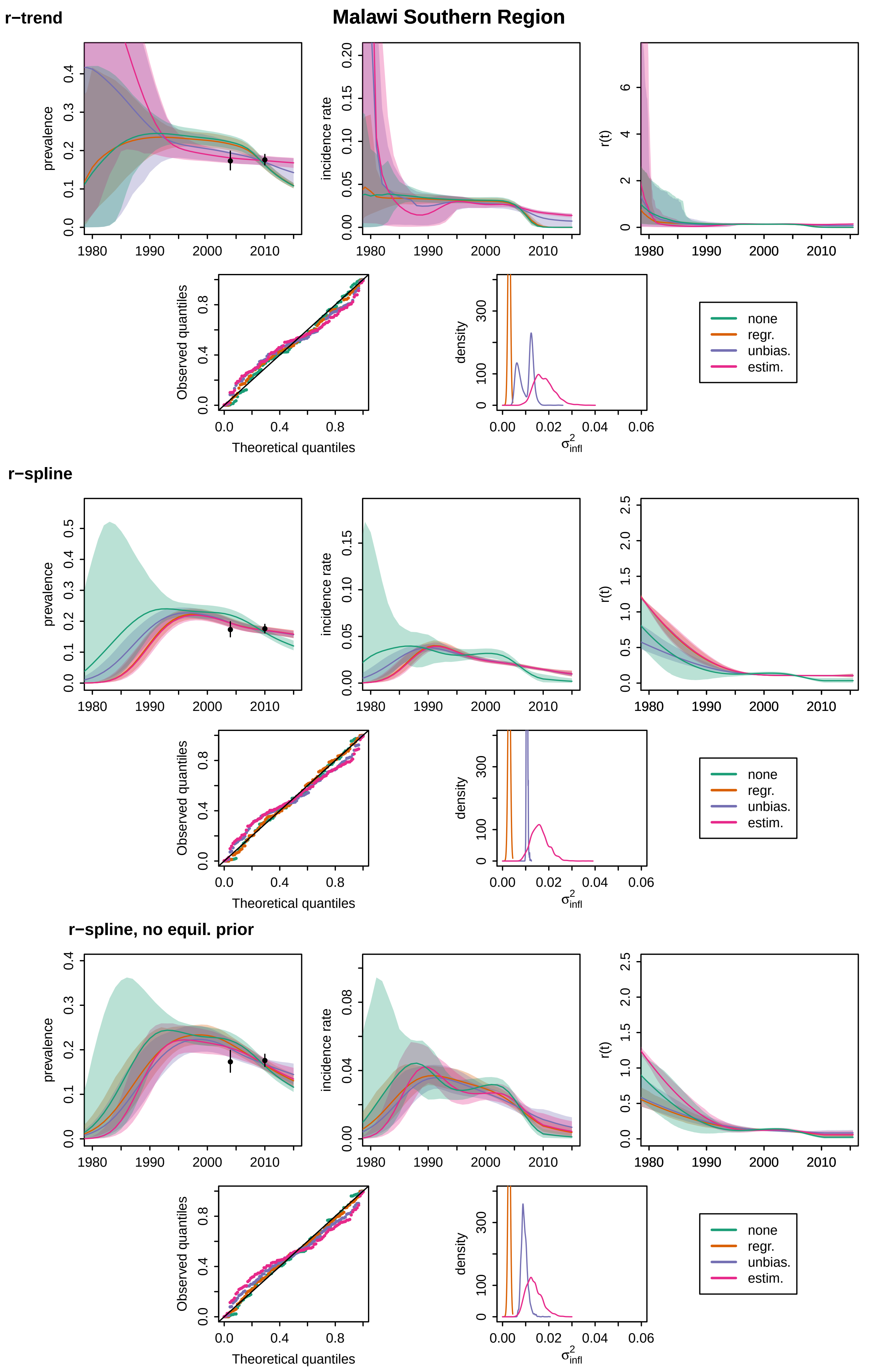

Malawi Southern Region
r−trend
prevalence
incidence rate
r(t)
Observed quantiles
Theoretical quantiles
density
σ^2_{infl}
none
regr.
unbias.
estim.
r−spline
prevalence
incidence rate
r(t)
Observed quantiles
Theoretical quantiles
density
σ^2_{infl}
none
regr.
unbias.
estim.
r−spline, no equil. prior
prevalence
incidence rate
r(t)
Observed quantiles
Theoretical quantiles
density
σ^2_{infl}
none
regr.
unbias.
estim.

United Republic of Tanzania Urban

r−trend

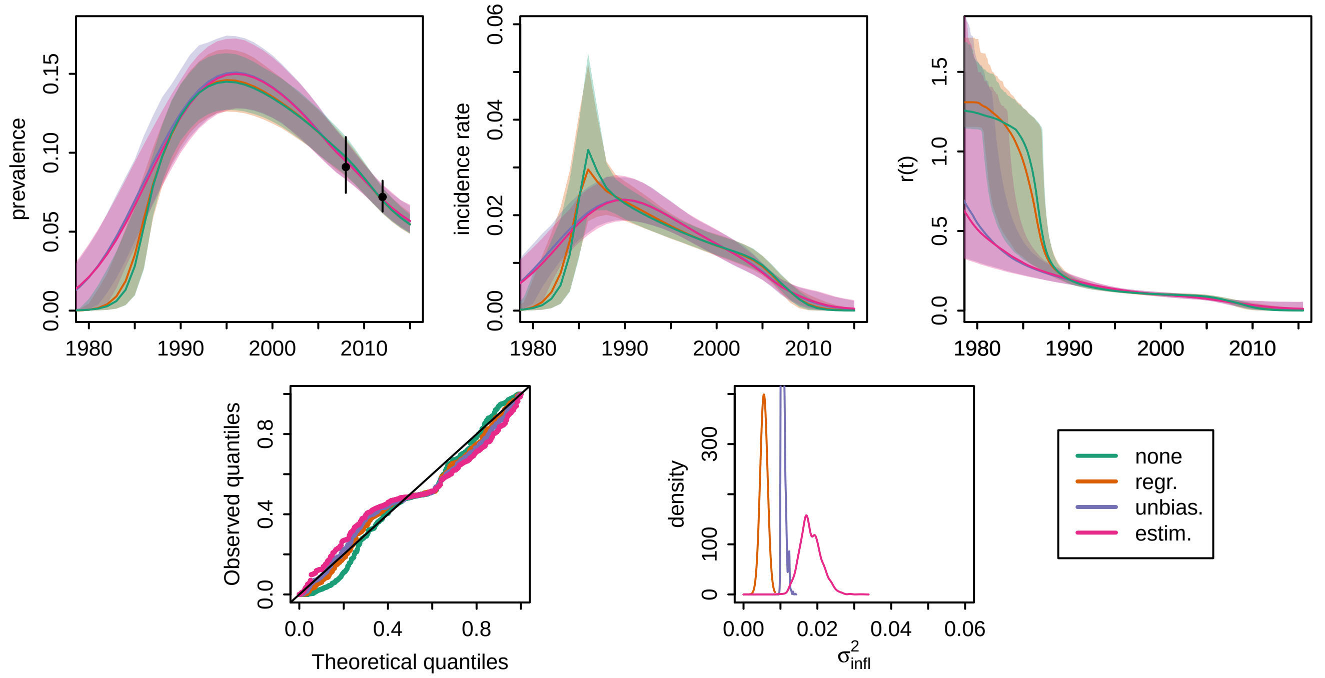

r−spline

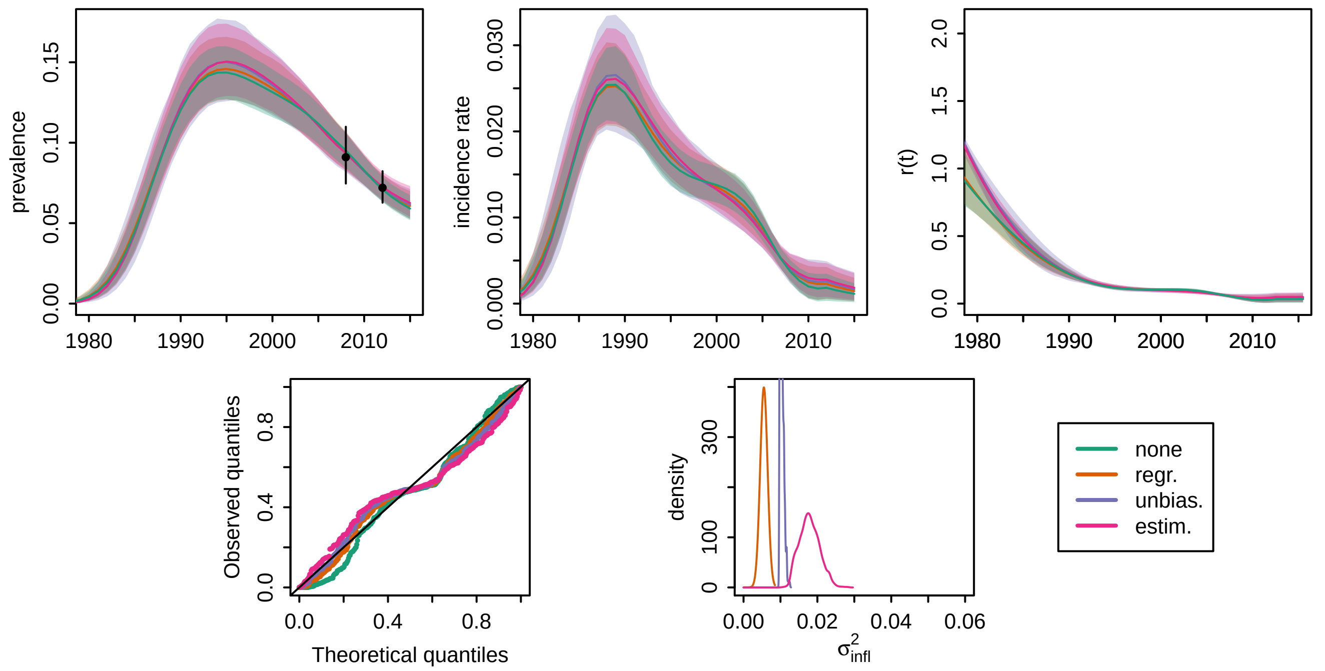

r−spline, no equil. prior

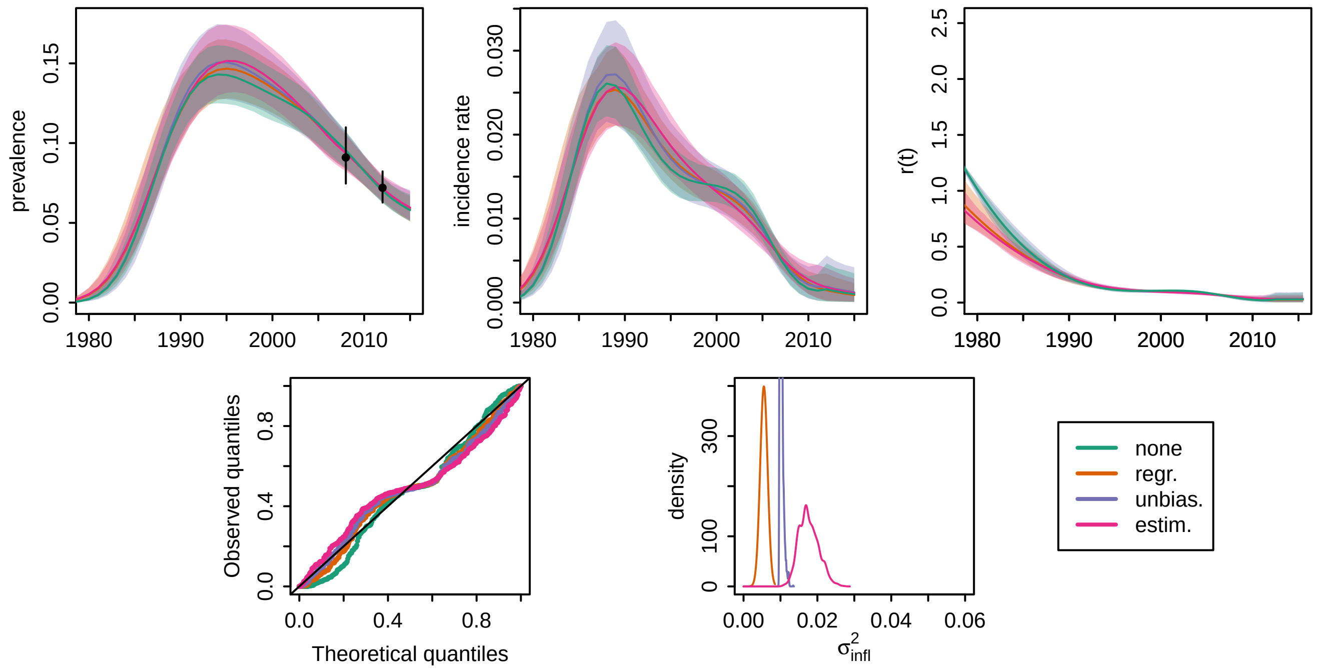

r−trend

United Republic of Tanzania Rural

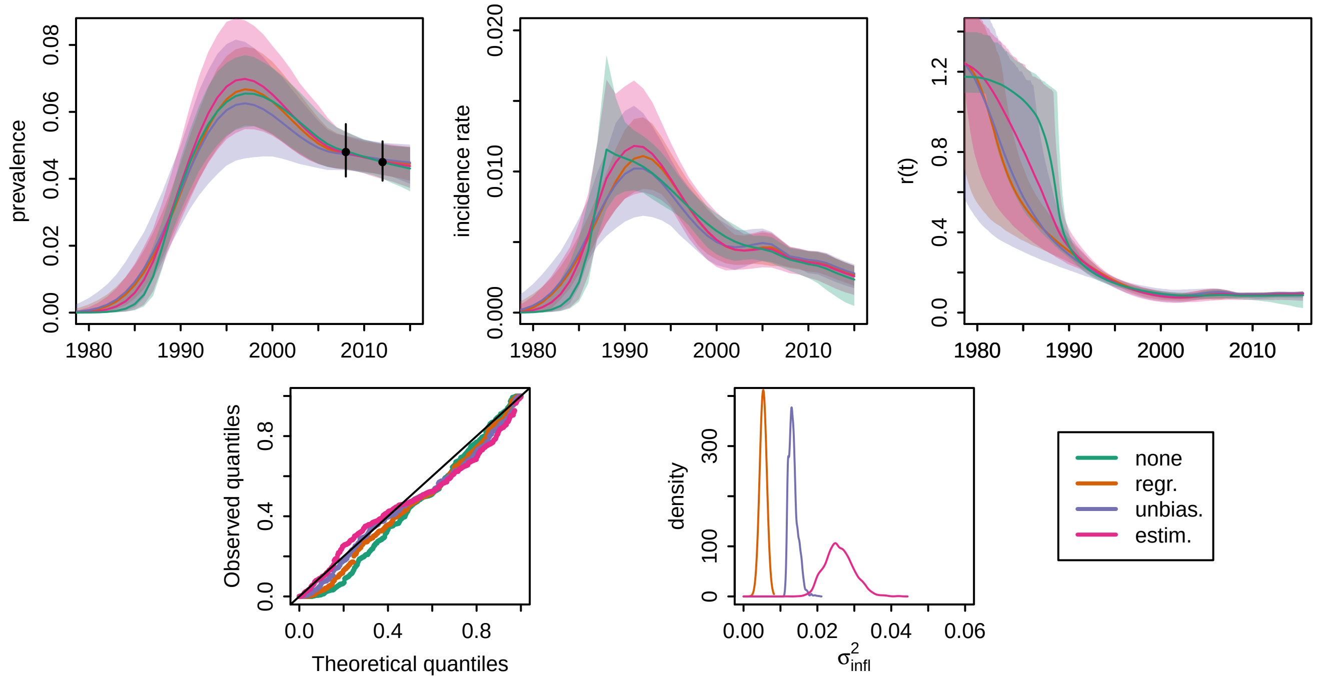

r−spline

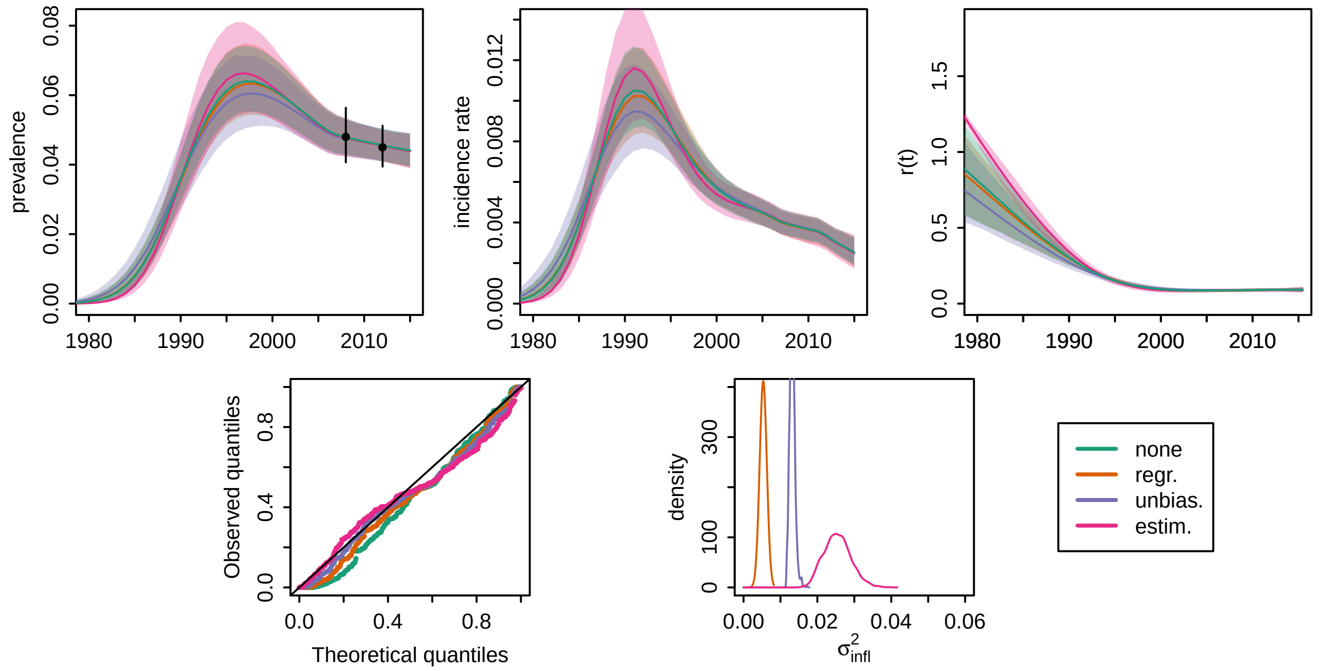

r−spline, no equil. prior

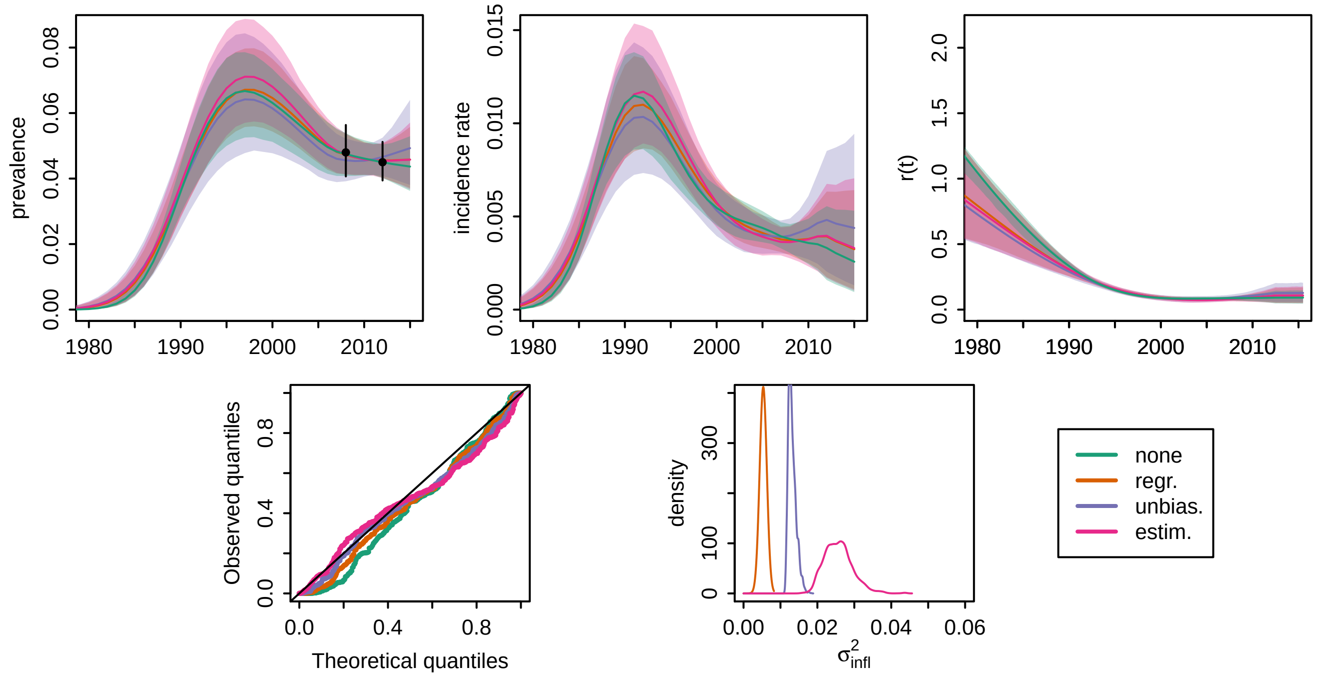

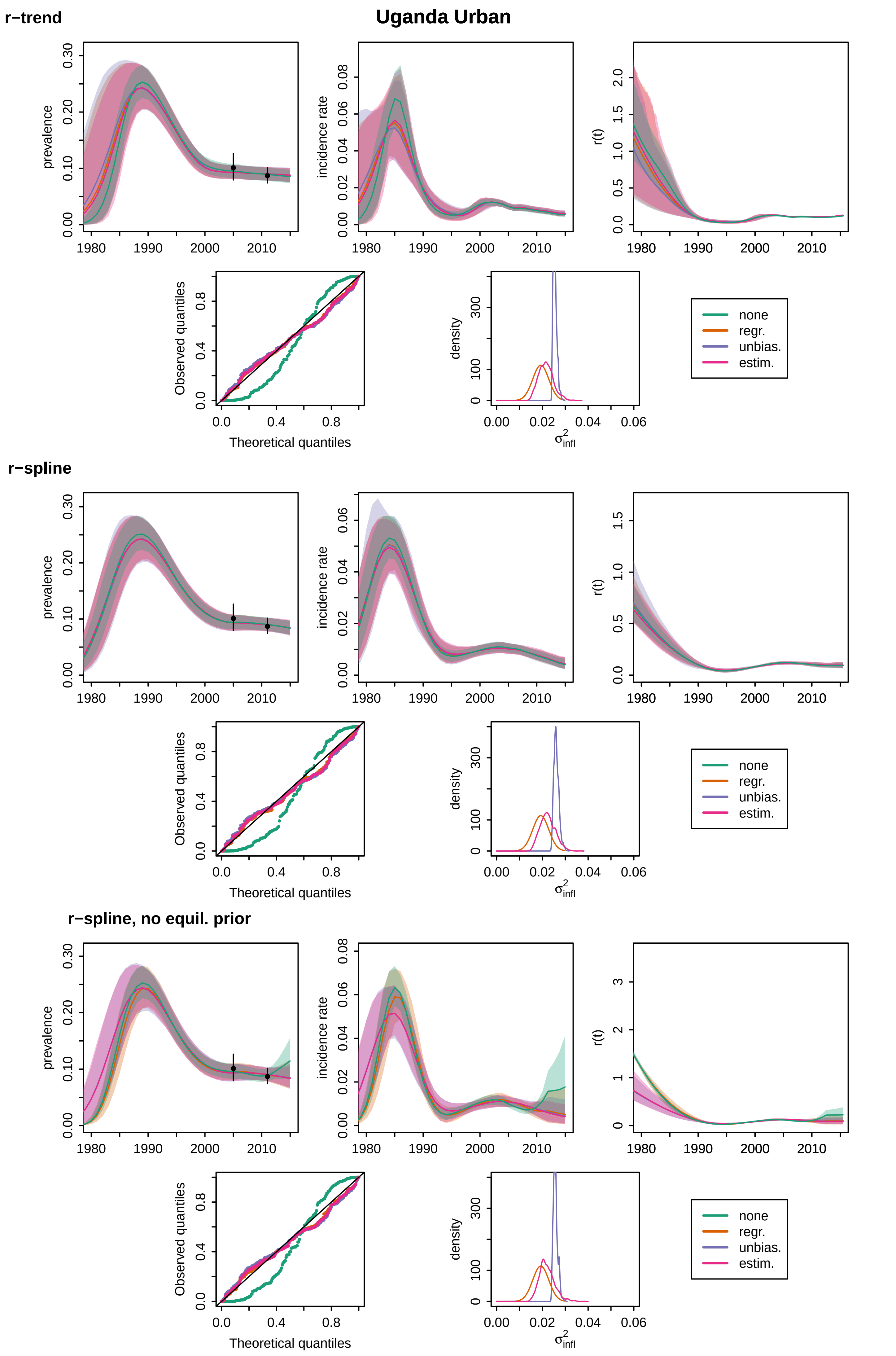

Uganda Urban
r−trend
r−spline
r−spline, no equil. prior
prevalence
incidence rate
r(t)
Observed quantiles
Theoretical quantiles
density
σ^2_{infl}
none
regr.
unbias.
estim.

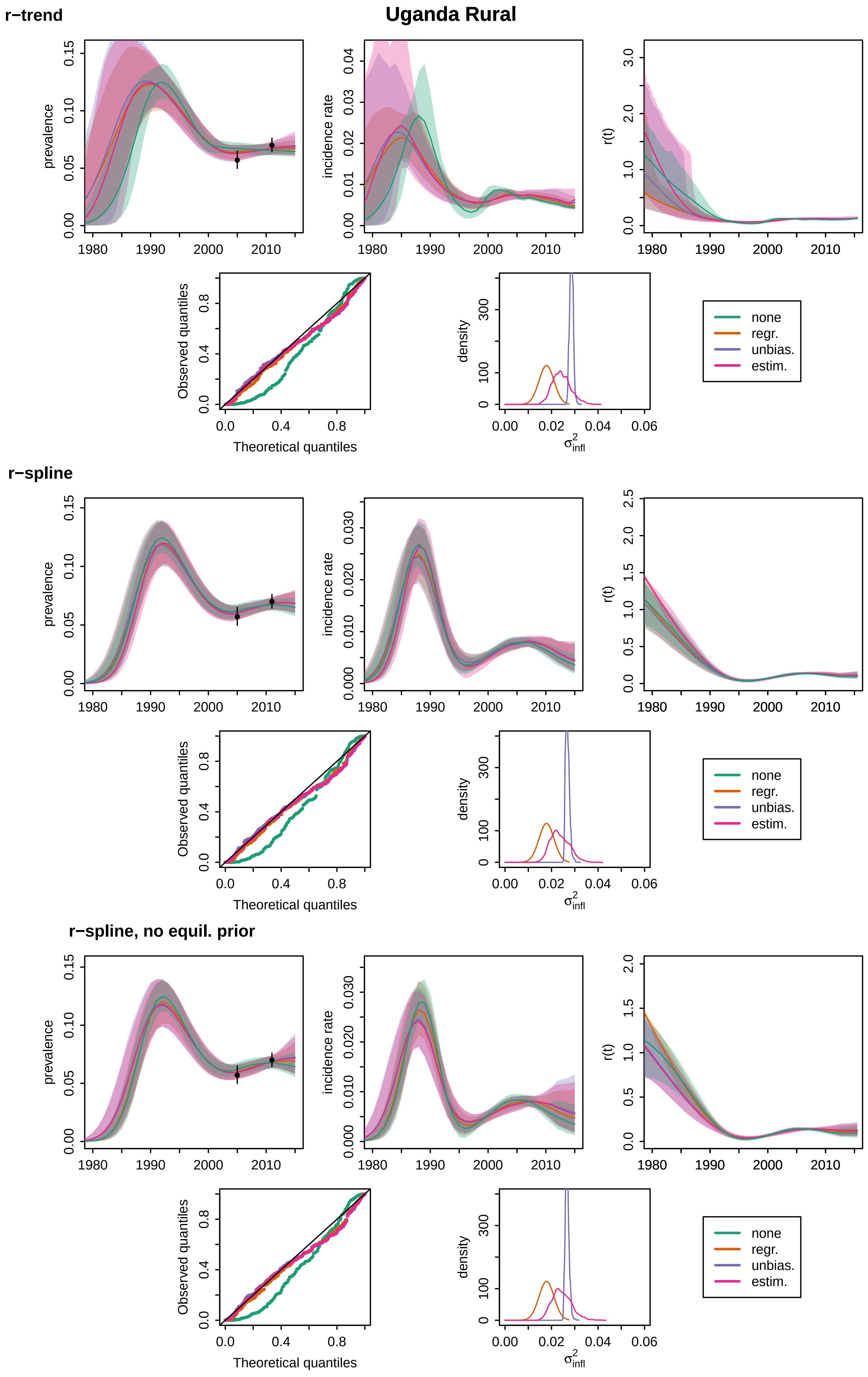

Uganda Rural
r−trend
r−spline
r−spline, no equil. prior
prevalence
incidence rate
r(t)
Observed quantiles
Theoretical quantiles
density
σ^2_{infl}
none
regr.
unbias.
estim.

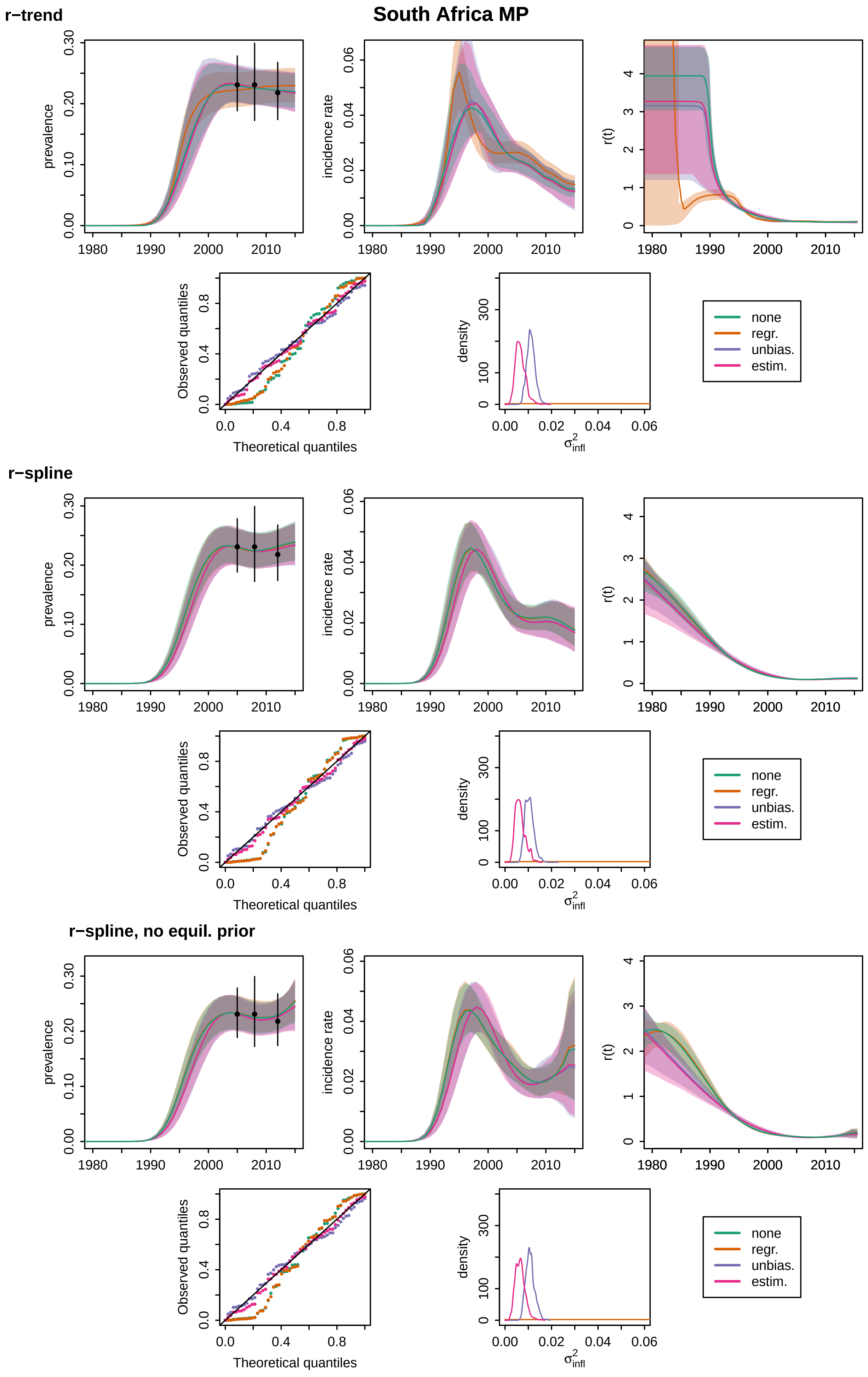
South Africa MP
r−trend
r−spline
r−spline, no equil. prior
prevalence
incidence rate
r(t)
Observed quantiles
Theoretical quantiles
density
σ^2_{infl}
none
regr.
unbias.
estim.
1980
1990
2000
2010
0.00
0.10
0.20
0.30
0.02
0.04
0.06
0.0
0.4
0.8
0
100
300
1
2
3
4

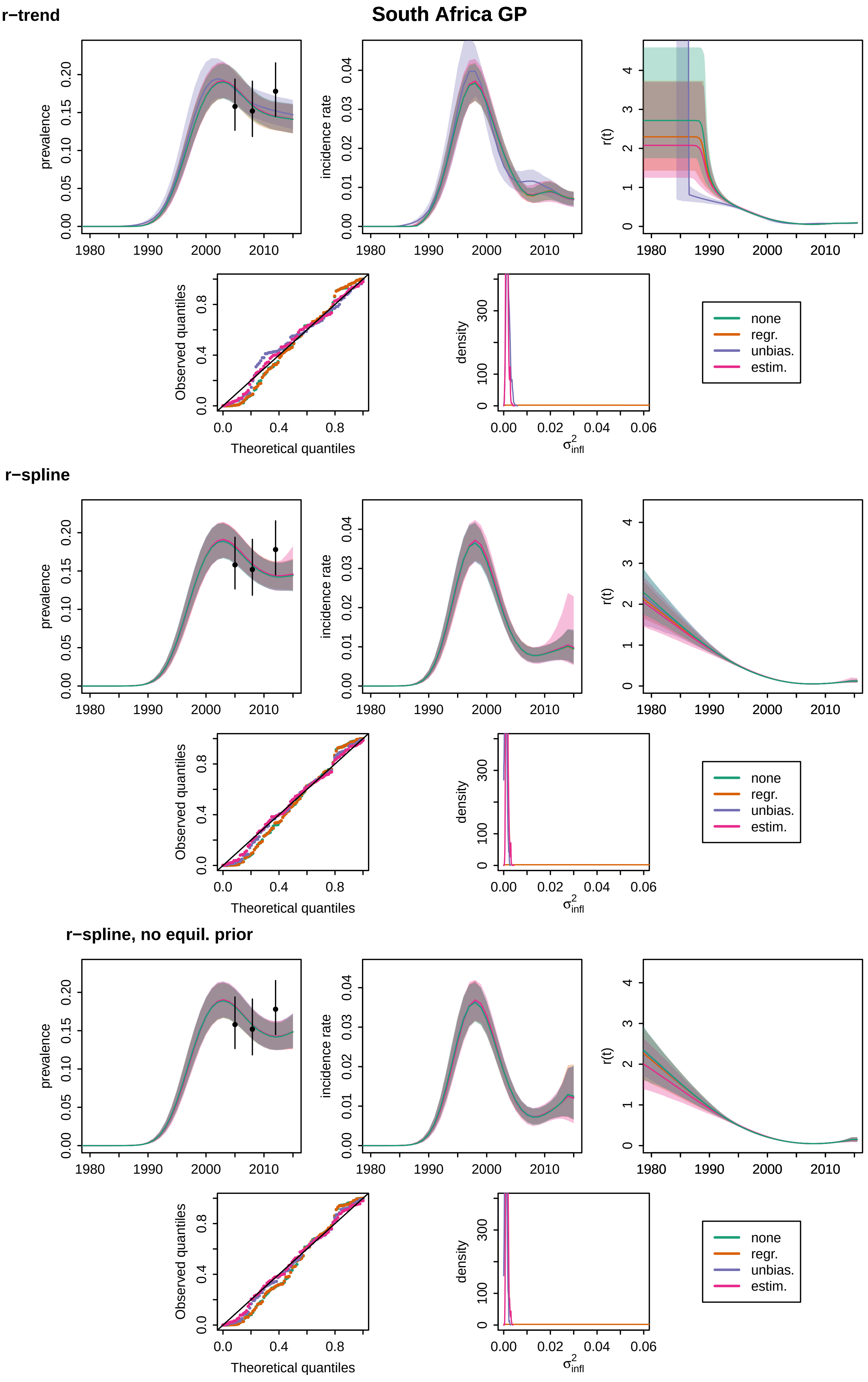

South Africa GP
r−trend
r−spline
r−spline, no equil. prior
prevalence
incidence rate
r(t)
Observed quantiles
Theoretical quantiles
density
σ^2_{infl}
none
regr.
unbias.
estim.

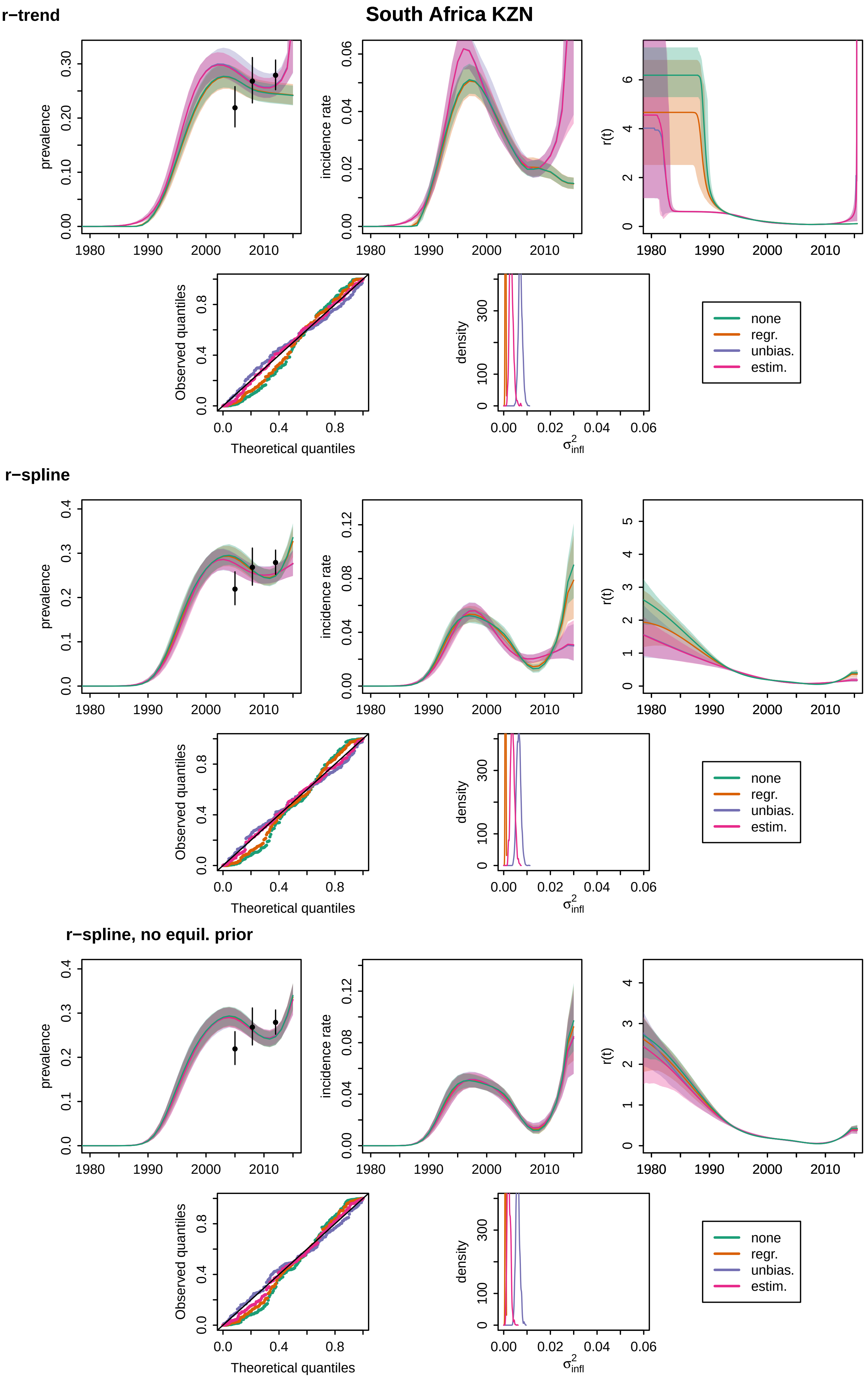

South Africa KZN
r−trend
r−spline
r−spline, no equil. prior
prevalence
incidence rate
r(t)
Observed quantiles
Theoretical quantiles
density
σ²infl
none
regr.
unbias.
estim.

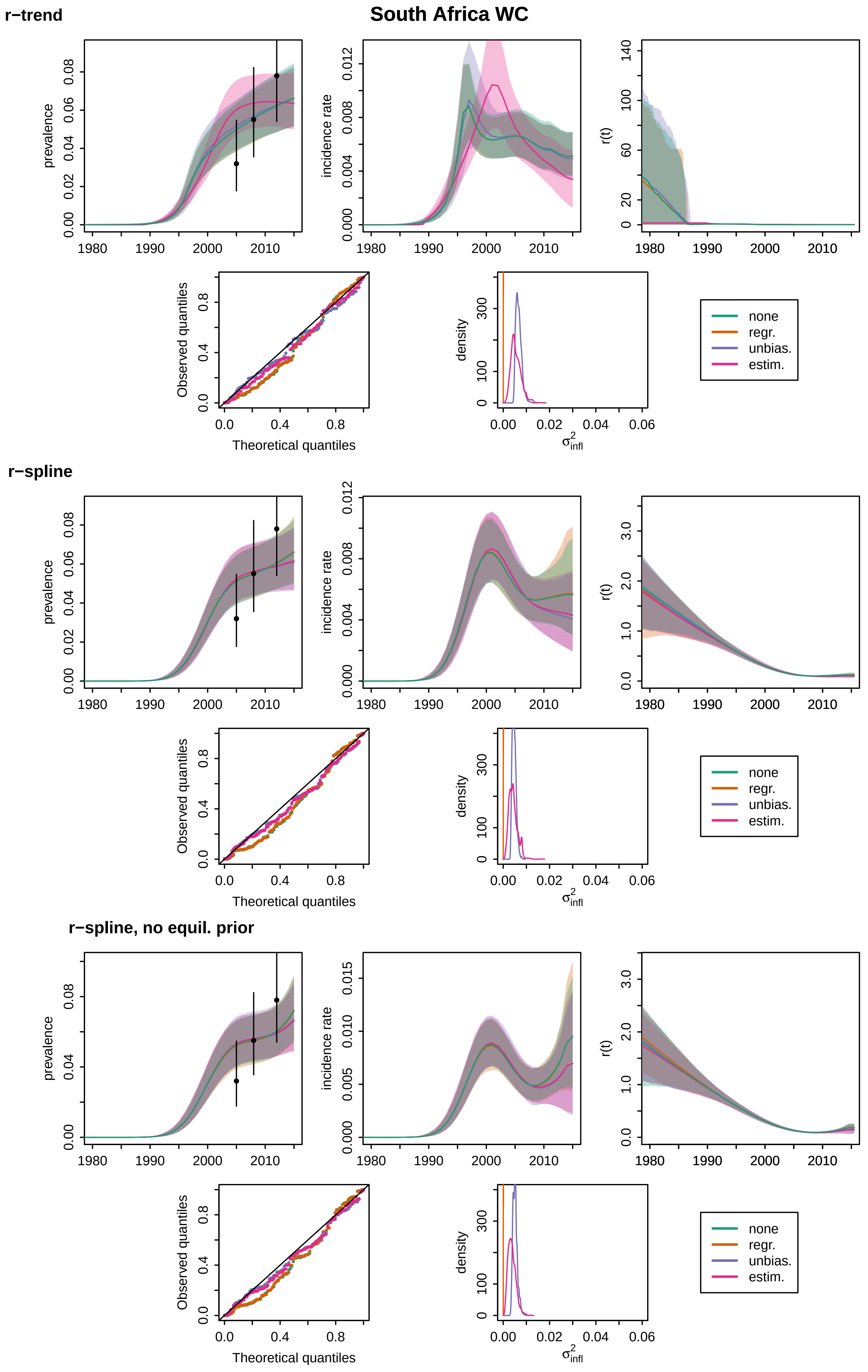

South Africa WC
r−trend
r−spline
r−spline, no equil. prior
prevalence
incidence rate
r(t)
Observed quantiles
Theoretical quantiles
density
σ^2_{infl}
none
regr.
unbias.
estim.

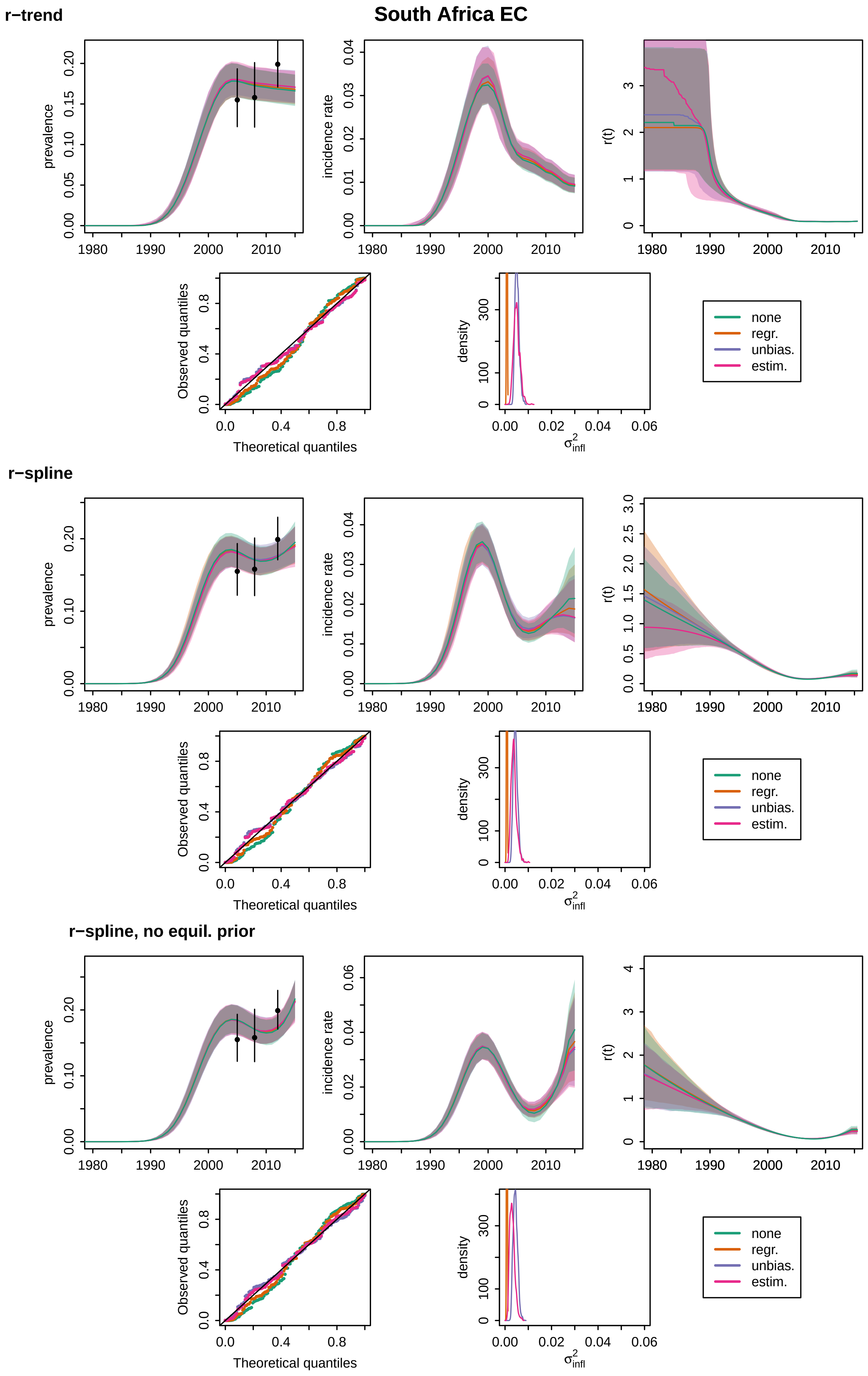
South Africa EC
r−trend
r−spline
r−spline, no equil. prior
prevalence
incidence rate
r(t)
Observed quantiles
Theoretical quantiles
density
σ^2_{infl}
none
regr.
unbias.
estim.

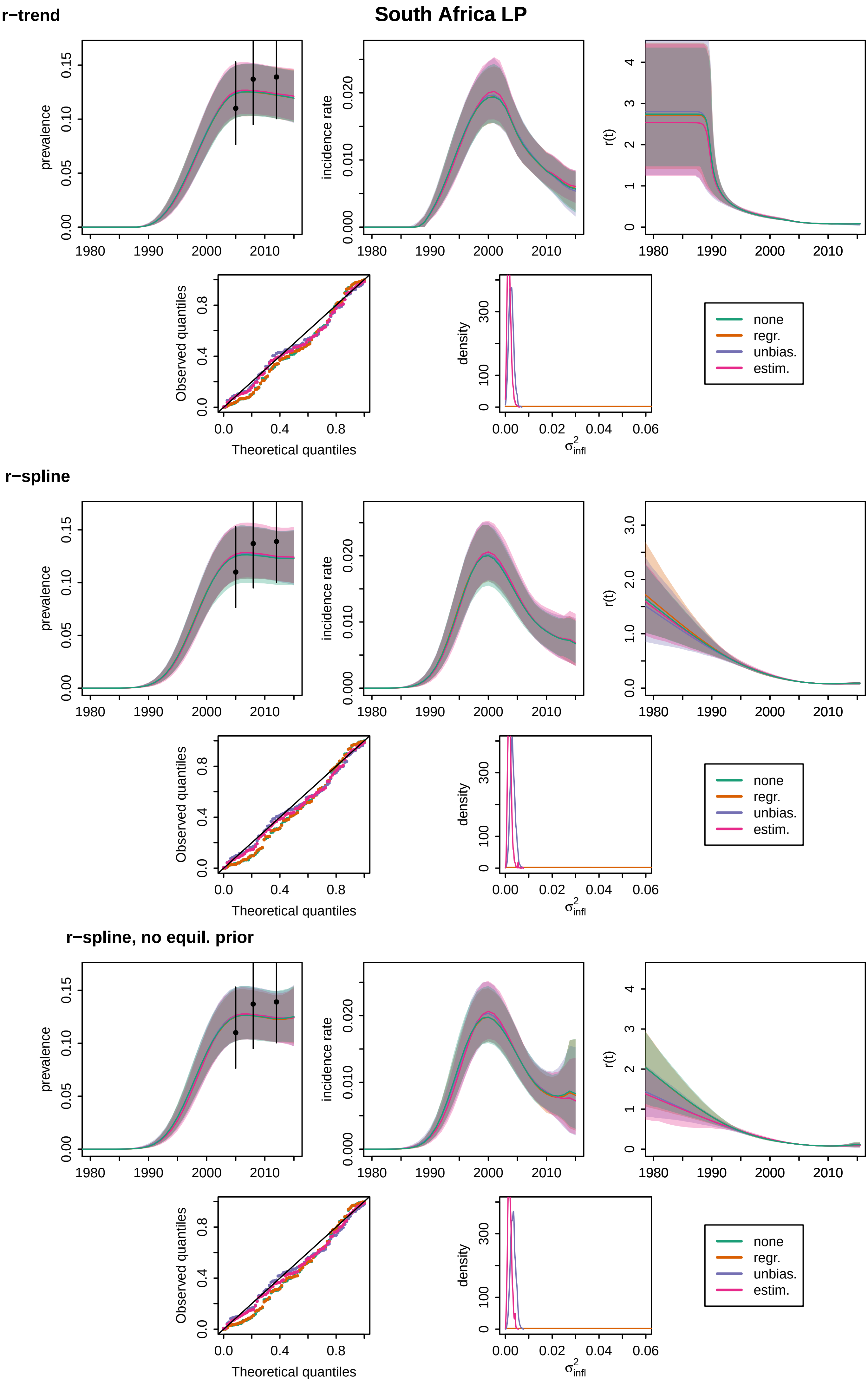
South Africa LP
r−trend
r−spline
r−spline, no equil. prior
prevalence
incidence rate
r(t)
Observed quantiles
Theoretical quantiles
density
σ^2_{infl}
none
regr.
unbias.
estim.

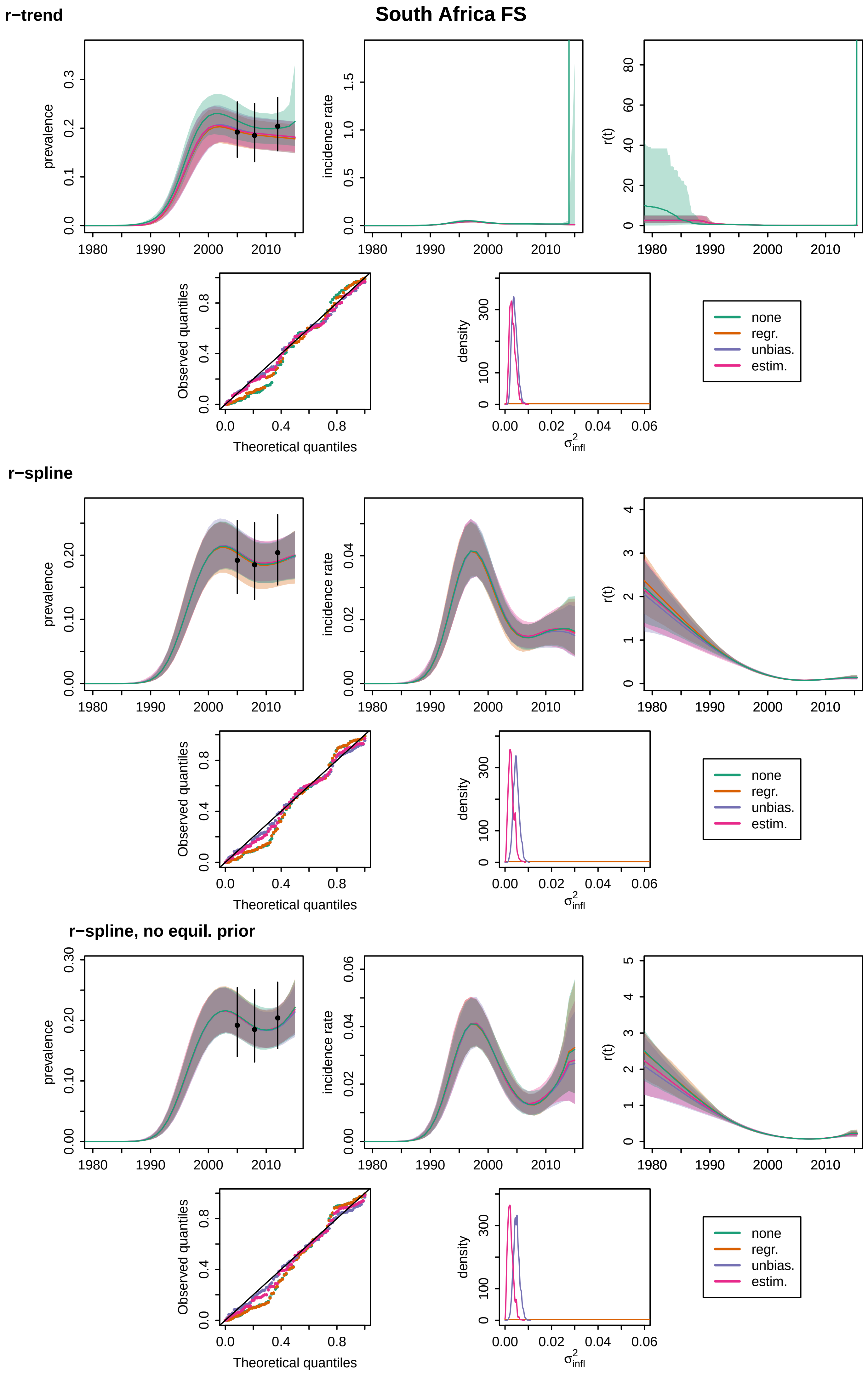
South Africa FS
r−trend
r−spline
r−spline, no equil. prior
prevalence
incidence rate
r(t)
Observed quantiles
Theoretical quantiles
density
σ^2_{infl}
none
regr.
unbias.
estim.
1980
1990
2000
2010

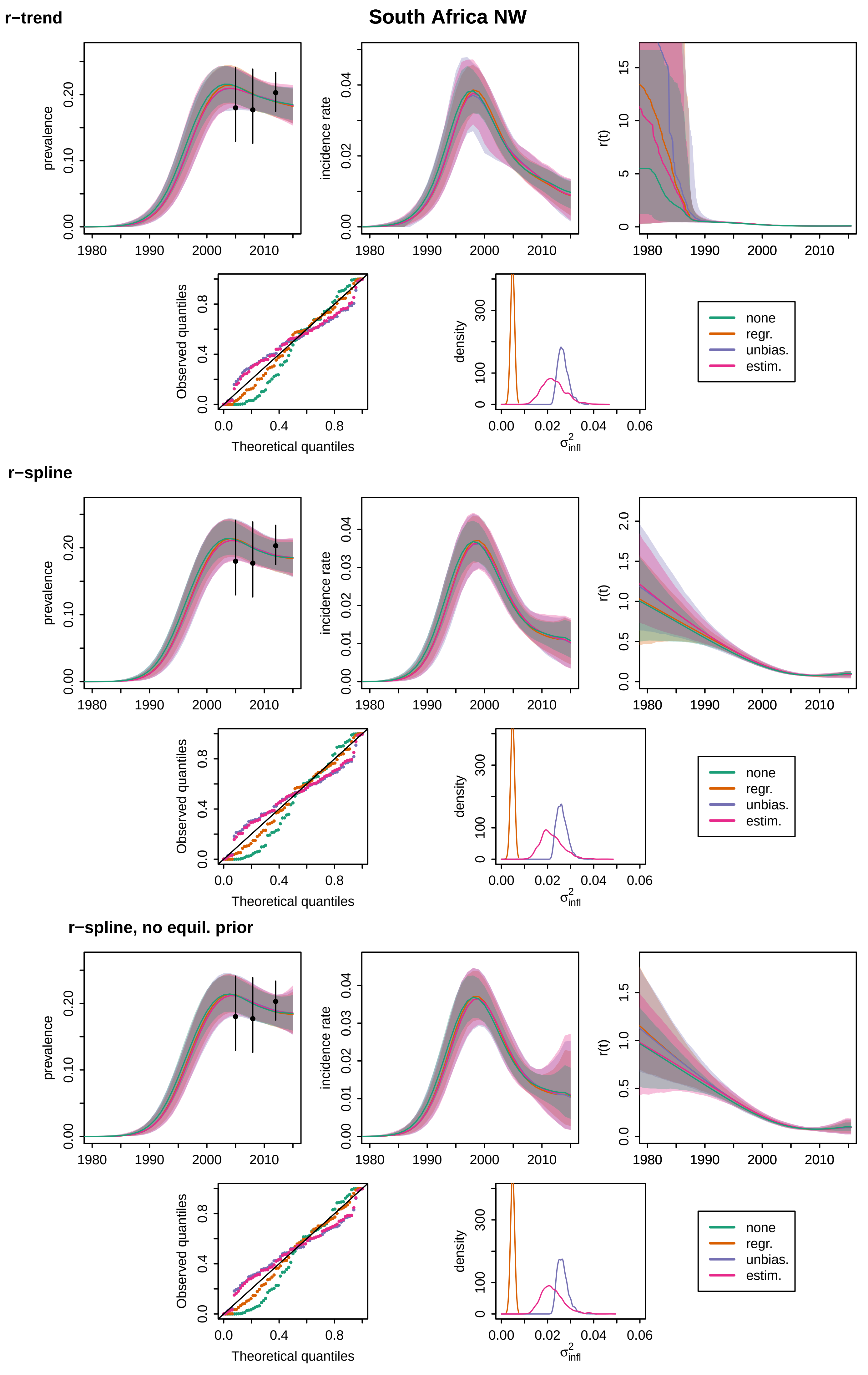
South Africa NW
r−trend
prevalence
0.00
0.10
0.20
1980
1990
2000
2010
incidence rate
0.00
0.02
0.04
r(t)
0
5
10
15
Observed quantiles
Theoretical quantiles
0.0
0.4
0.8
density
0
100
300
0.00
0.02
0.04
0.06
σ^2_{infl}
none
regr.
unbias.
estim.
r−spline
prevalence
incidence rate
0.00
0.01
0.02
0.03
0.04
r(t)
0.0
0.5
1.0
1.5
2.0
Observed quantiles
Theoretical quantiles
density
σ^2_{infl}
none
regr.
unbias.
estim.
r−spline, no equil. prior
prevalence
incidence rate
r(t)
0.0
0.5
1.0
1.5
Observed quantiles
Theoretical quantiles
density
σ^2_{infl}
none
regr.
unbias.
estim.

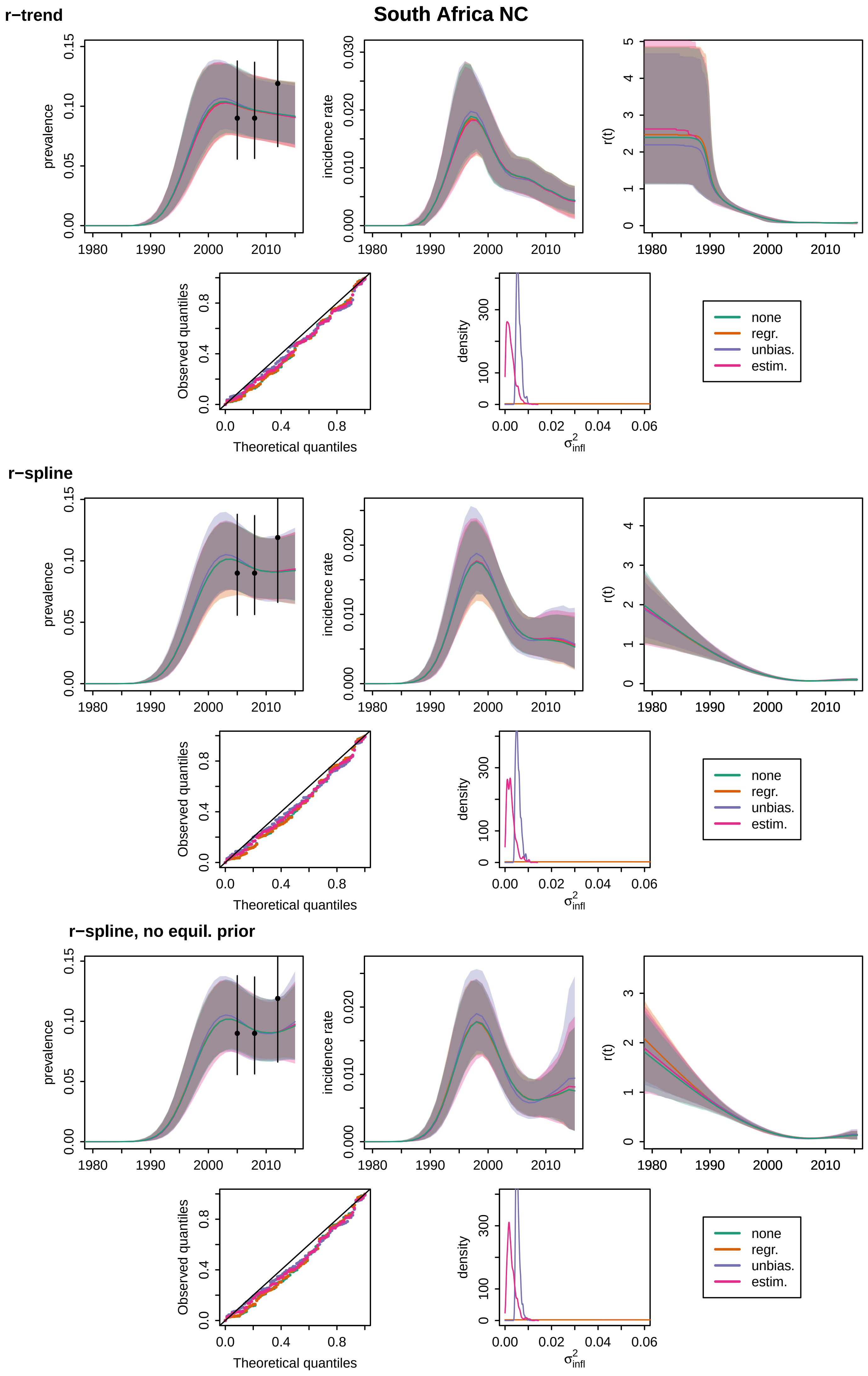
South Africa NC
r−trend
r−spline
r−spline, no equil. prior
prevalence
incidence rate
r(t)
Observed quantiles
Theoretical quantiles
density
σ^2_{infl}
none
regr.
unbias.
estim.

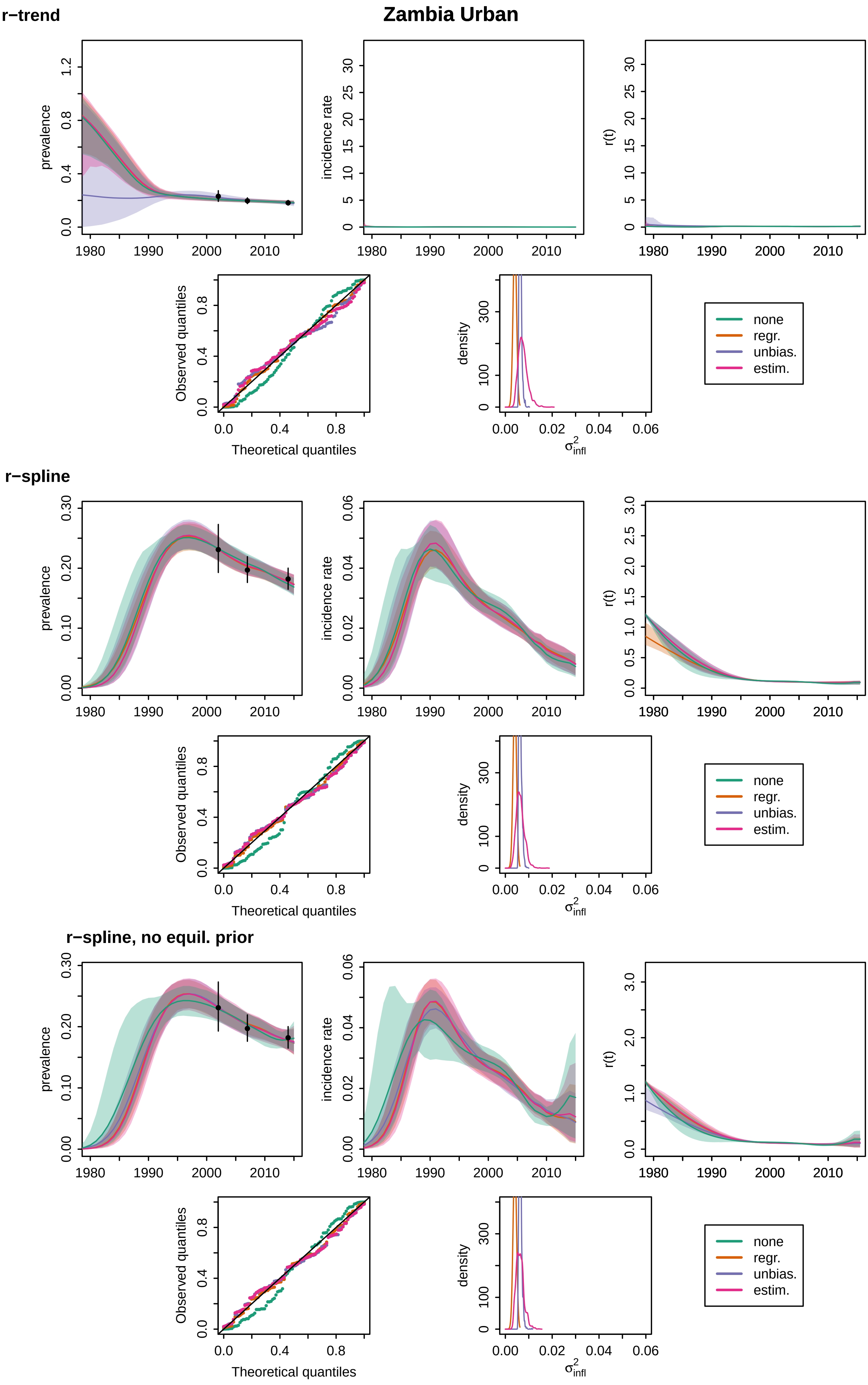
Zambia Urban
r−trend
prevalence
incidence rate
r(t)
Observed quantiles
Theoretical quantiles
density
σ²infl
none
regr.
unbias.
estim.
r−spline
r−spline, no equil. prior

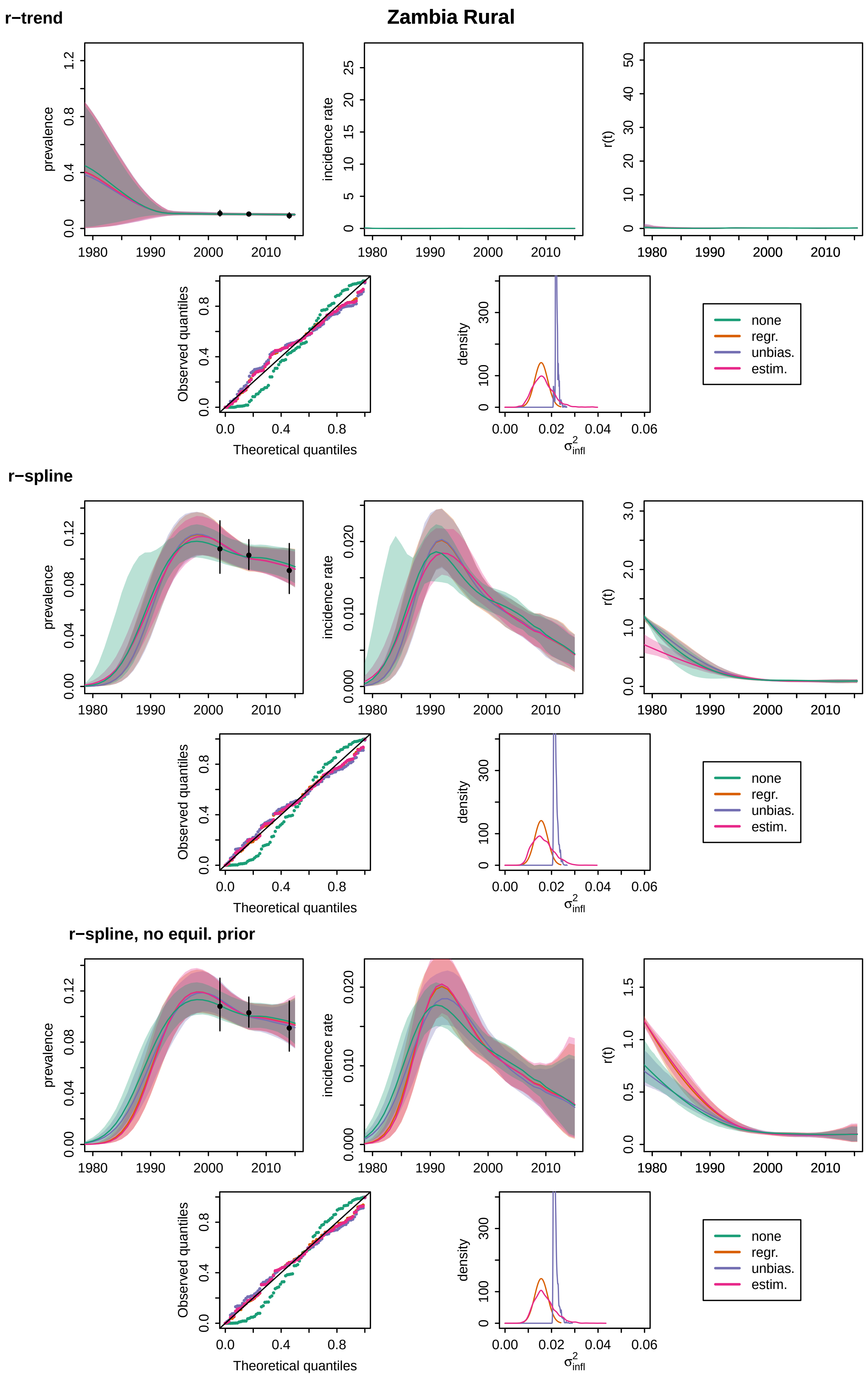
Zambia Rural
r−trend
r−spline
r−spline, no equil. prior
prevalence
incidence rate
r(t)
Observed quantiles
Theoretical quantiles
density
σ^2_{infl}
none
regr.
unbias.
estim.

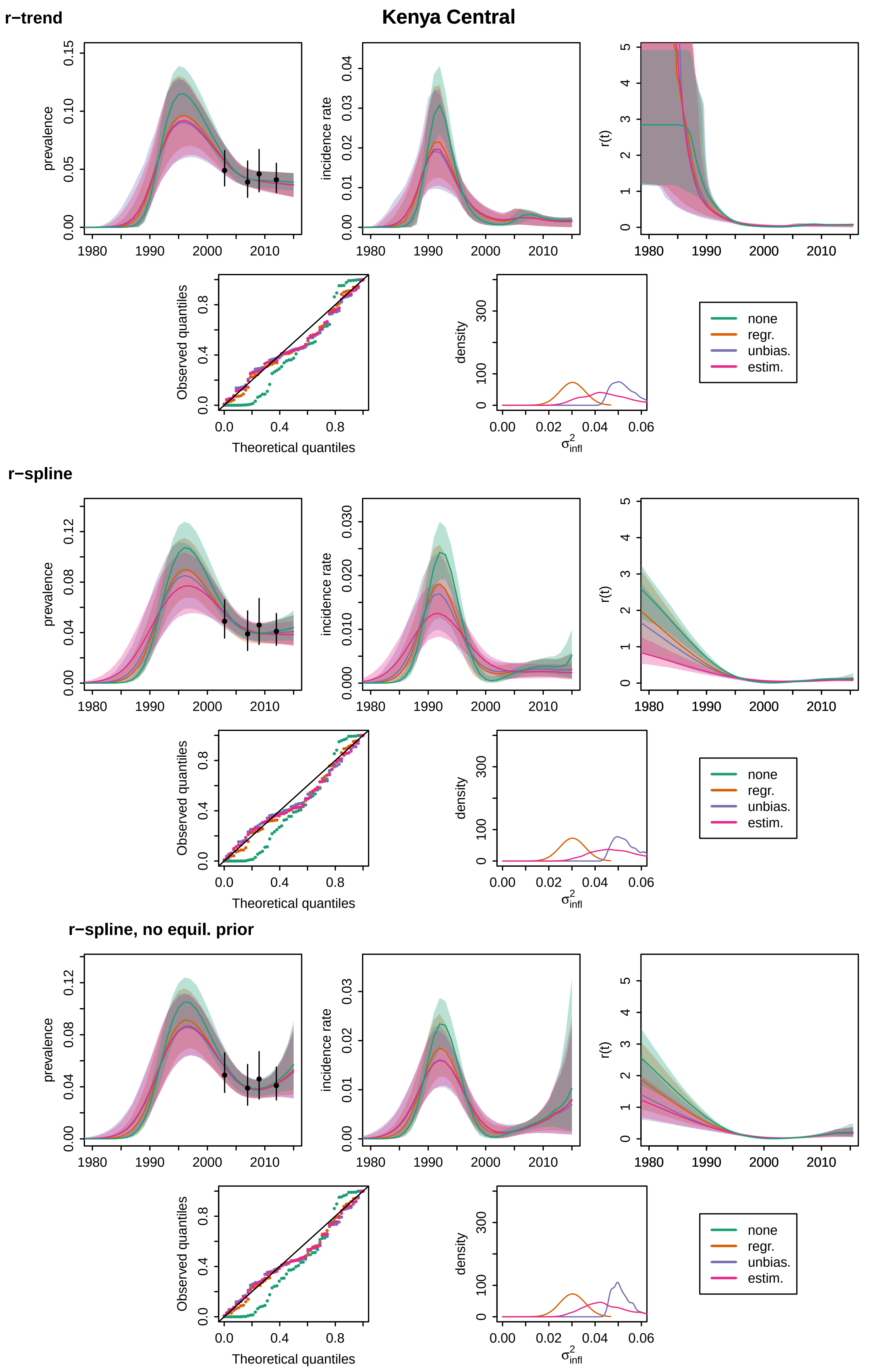

r−trend
Kenya Central
r−spline
r−spline, no equil. prior
prevalence
incidence rate
r(t)
Observed quantiles
Theoretical quantiles
density
σ^2_{infl}
none
regr.
unbias.
estim.

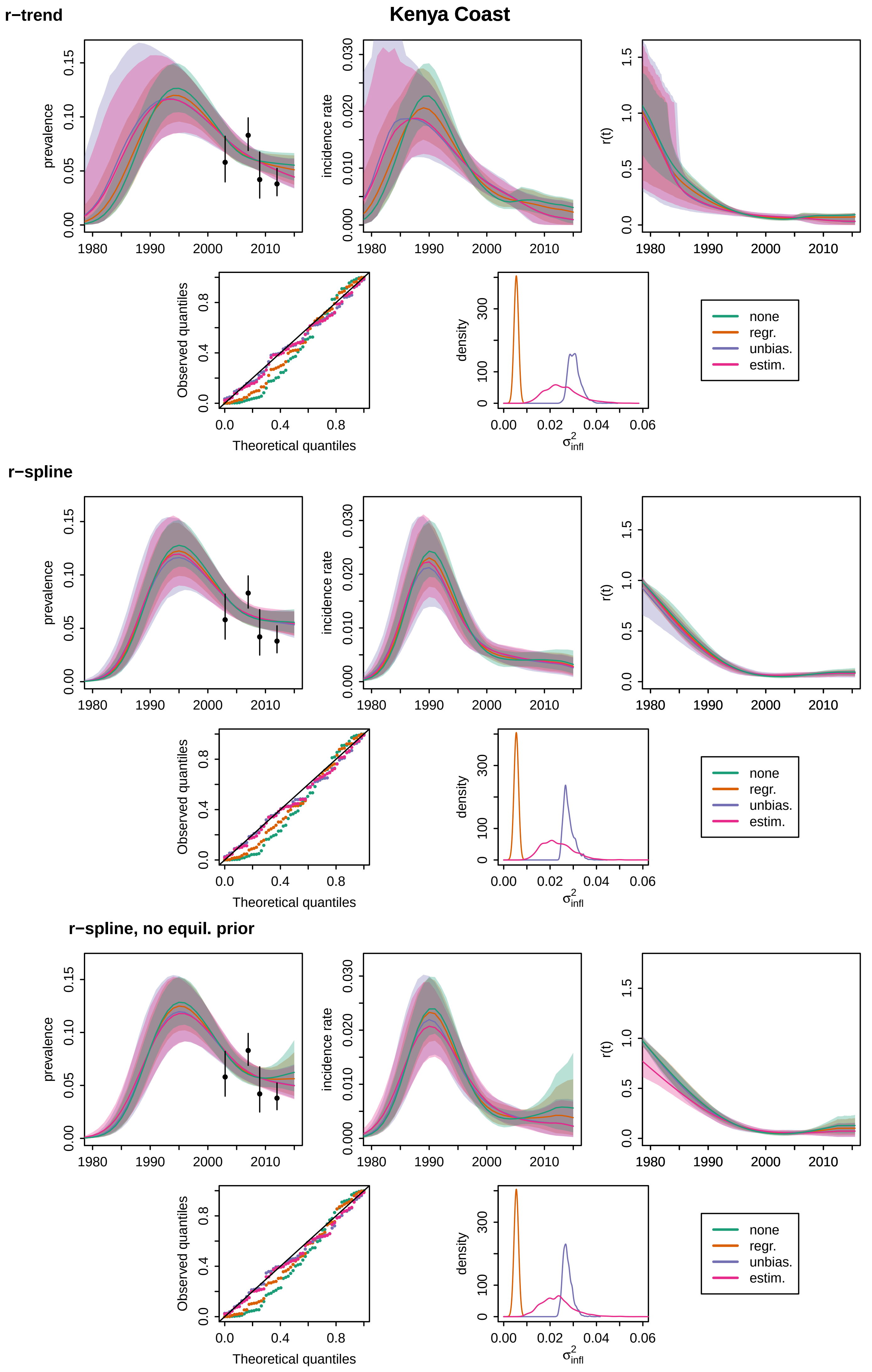
Kenya Coast
r−trend
r−spline
r−spline, no equil. prior
prevalence
incidence rate
r(t)
Observed quantiles
Theoretical quantiles
density
σ^2_{infl}
none
regr.
unbias.
estim.
1980
1990
2000
2010
0.00
0.05
0.10
0.15
0.000
0.010
0.020
0.030
0.0
0.5
1.0
1.5
0.4
0.8
0
100
300
0.02
0.04
0.06

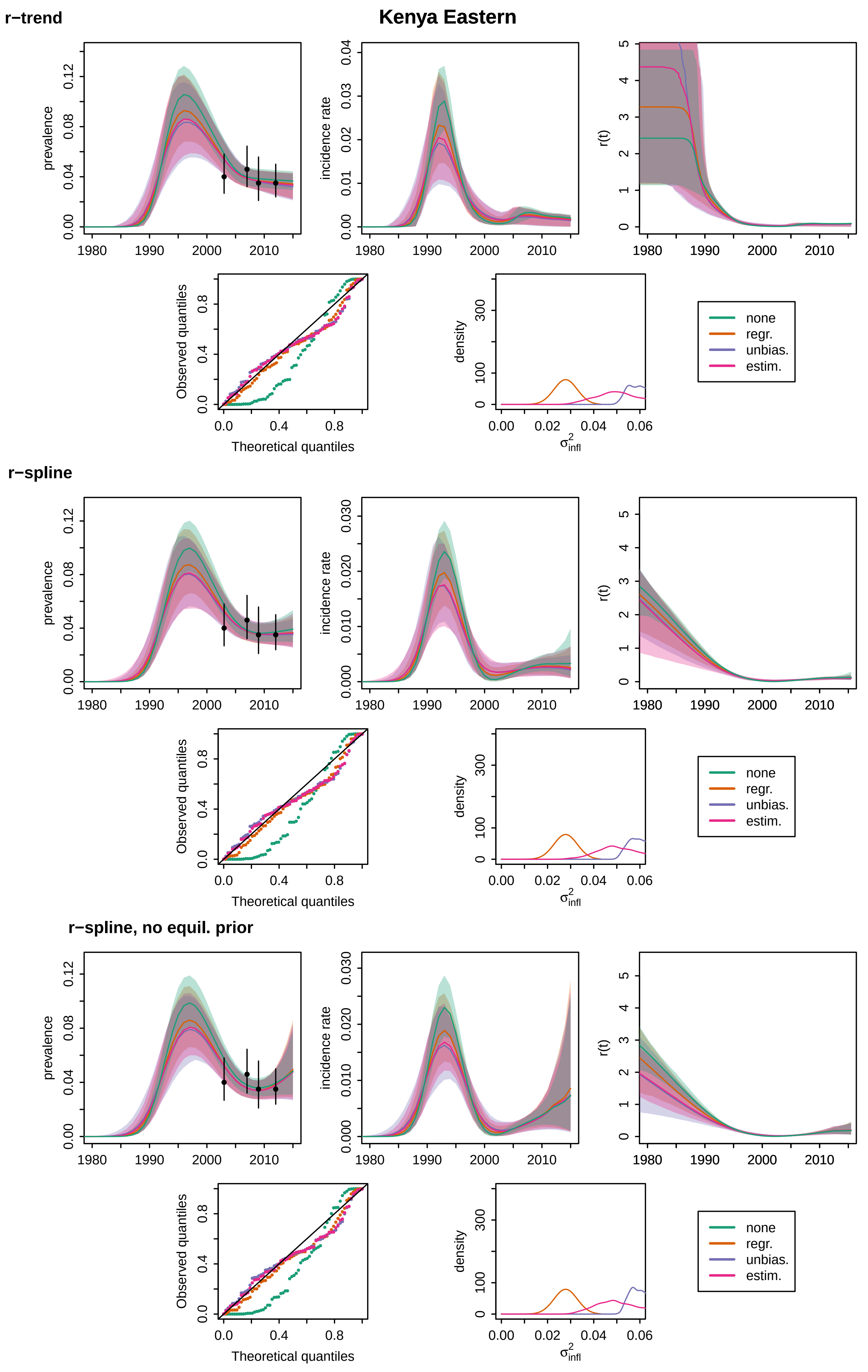
Kenya Eastern
r−trend
prevalence
incidence rate
r(t)
Observed quantiles
Theoretical quantiles
density
σ²infl
none
regr.
unbias.
estim.
r−spline
prevalence
incidence rate
r(t)
Observed quantiles
Theoretical quantiles
density
σ²infl
none
regr.
unbias.
estim.
r−spline, no equil. prior
prevalence
incidence rate
r(t)
Observed quantiles
Theoretical quantiles
density
σ²infl
none
regr.
unbias.
estim.

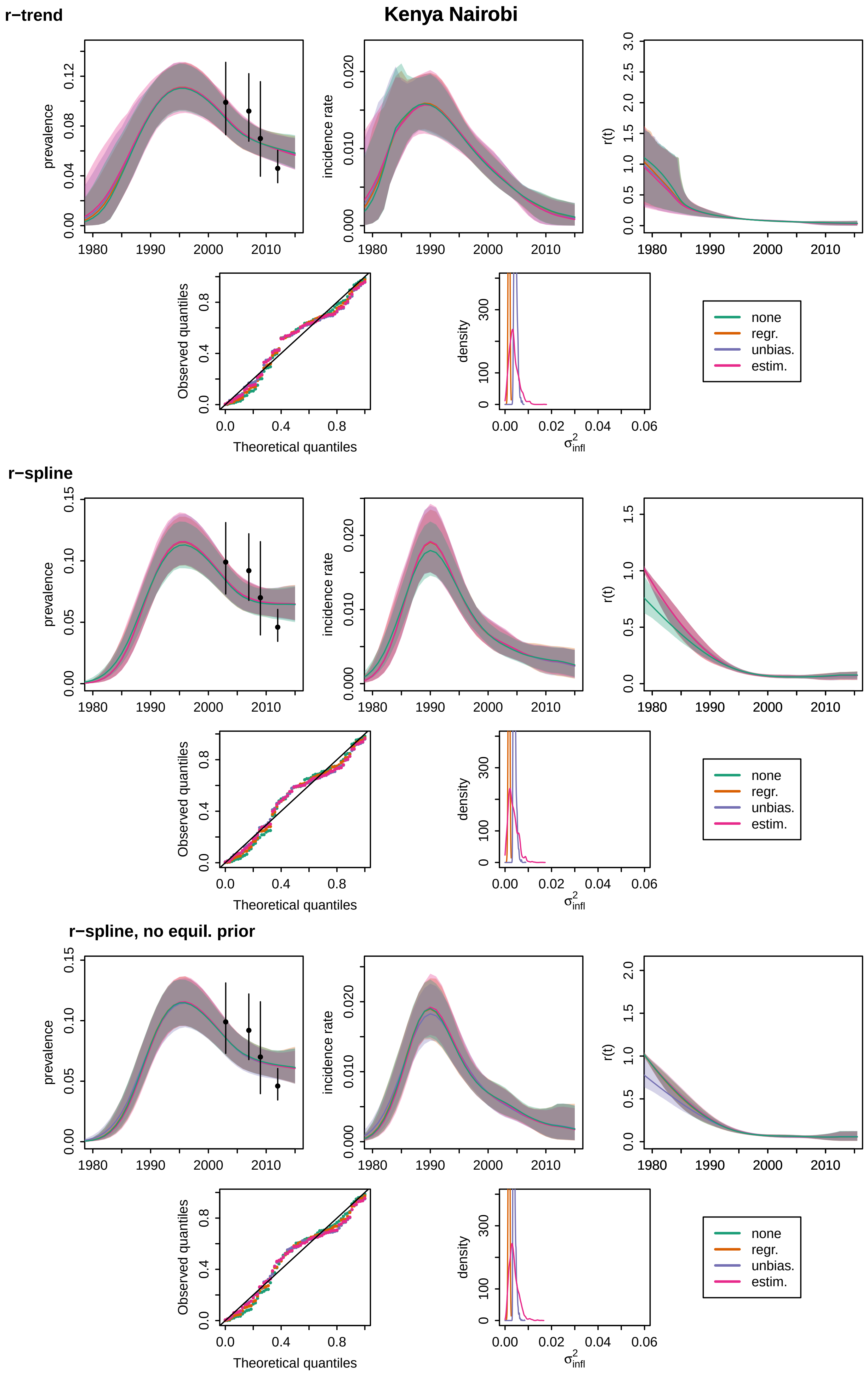
Kenya Nairobi
r−trend
r−spline
r−spline, no equil. prior
prevalence
incidence rate
r(t)
Observed quantiles
Theoretical quantiles
density
σ^2_{infl}
none
regr.
unbias.
estim.
1980
1990
2000
2010
0.00
0.04
0.08
0.12
0.05
0.10
0.15
0.000
0.010
0.020
0.0
0.5
1.0
1.5
2.0
2.5
3.0
0.4
0.8
0
100
300
0.02
0.06

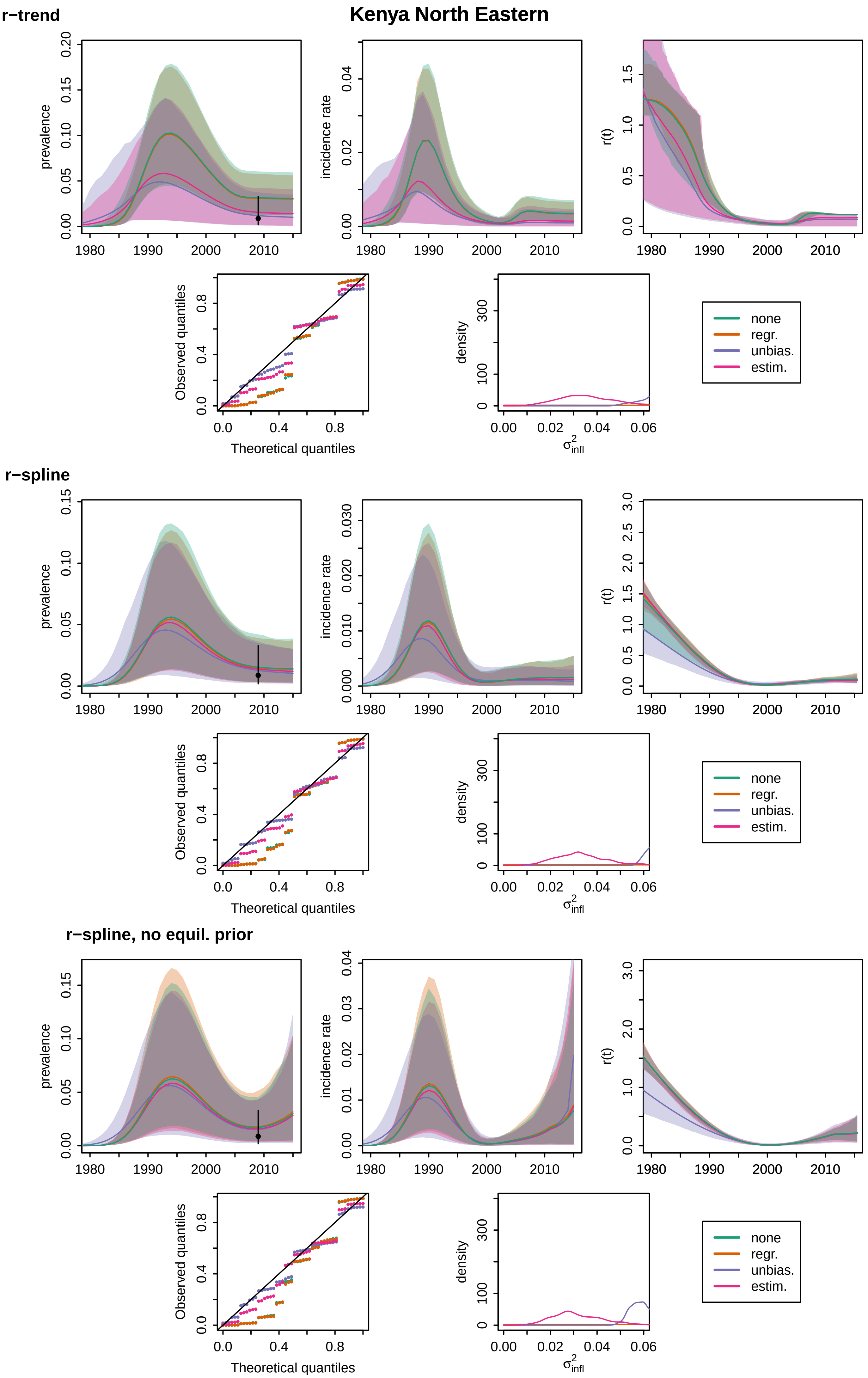
Kenya North Eastern
r−trend
r−spline
r−spline, no equil. prior
prevalence
incidence rate
r(t)
Observed quantiles
Theoretical quantiles
density
σ^2_{infl}
none
regr.
unbias.
estim.

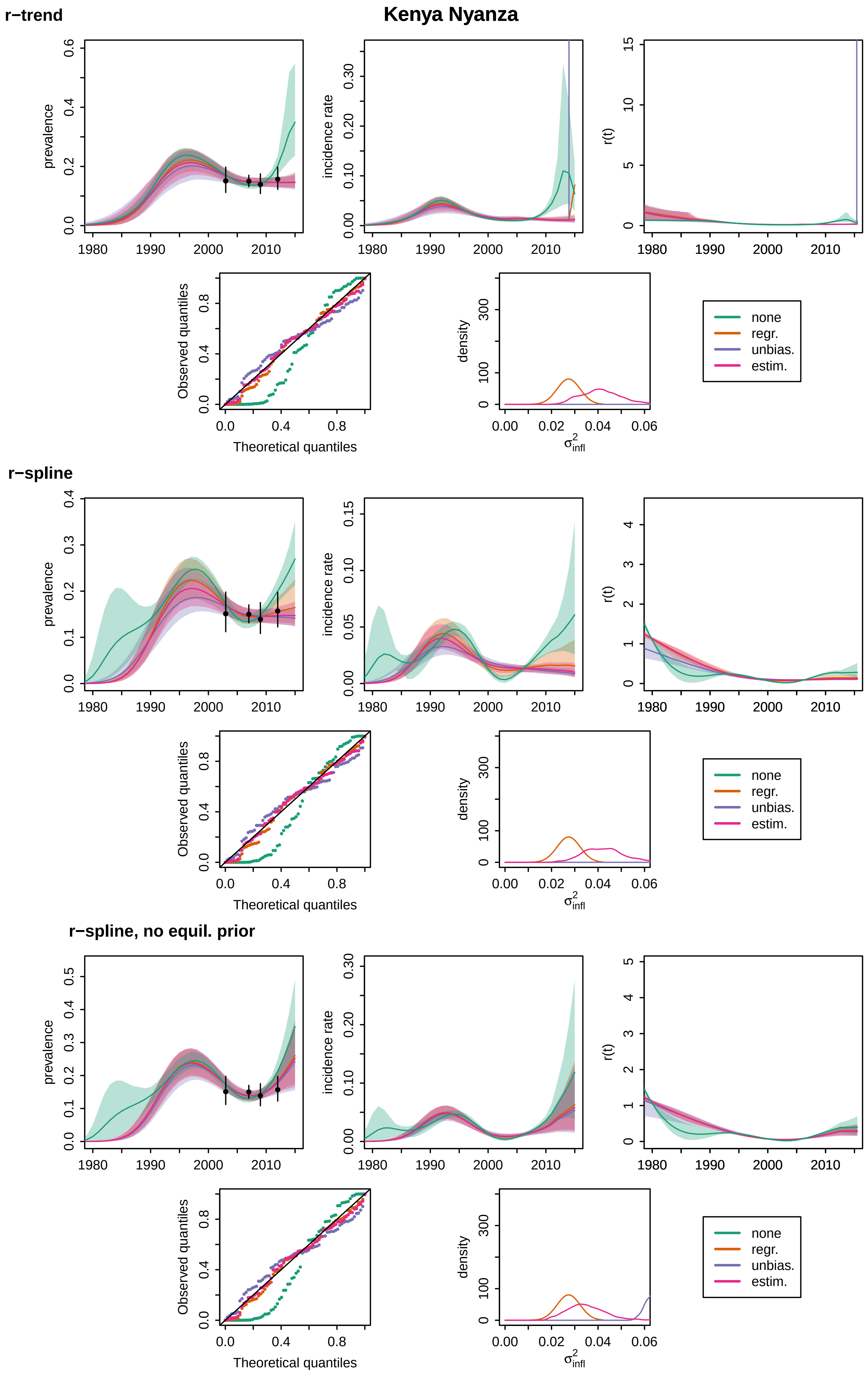
Kenya Nyanza
r−trend
r−spline
r−spline, no equil. prior
prevalence
incidence rate
r(t)
Observed quantiles
Theoretical quantiles
density
σ^2_{infl}
none
regr.
unbias.
estim.
1980
1990
2000
2010

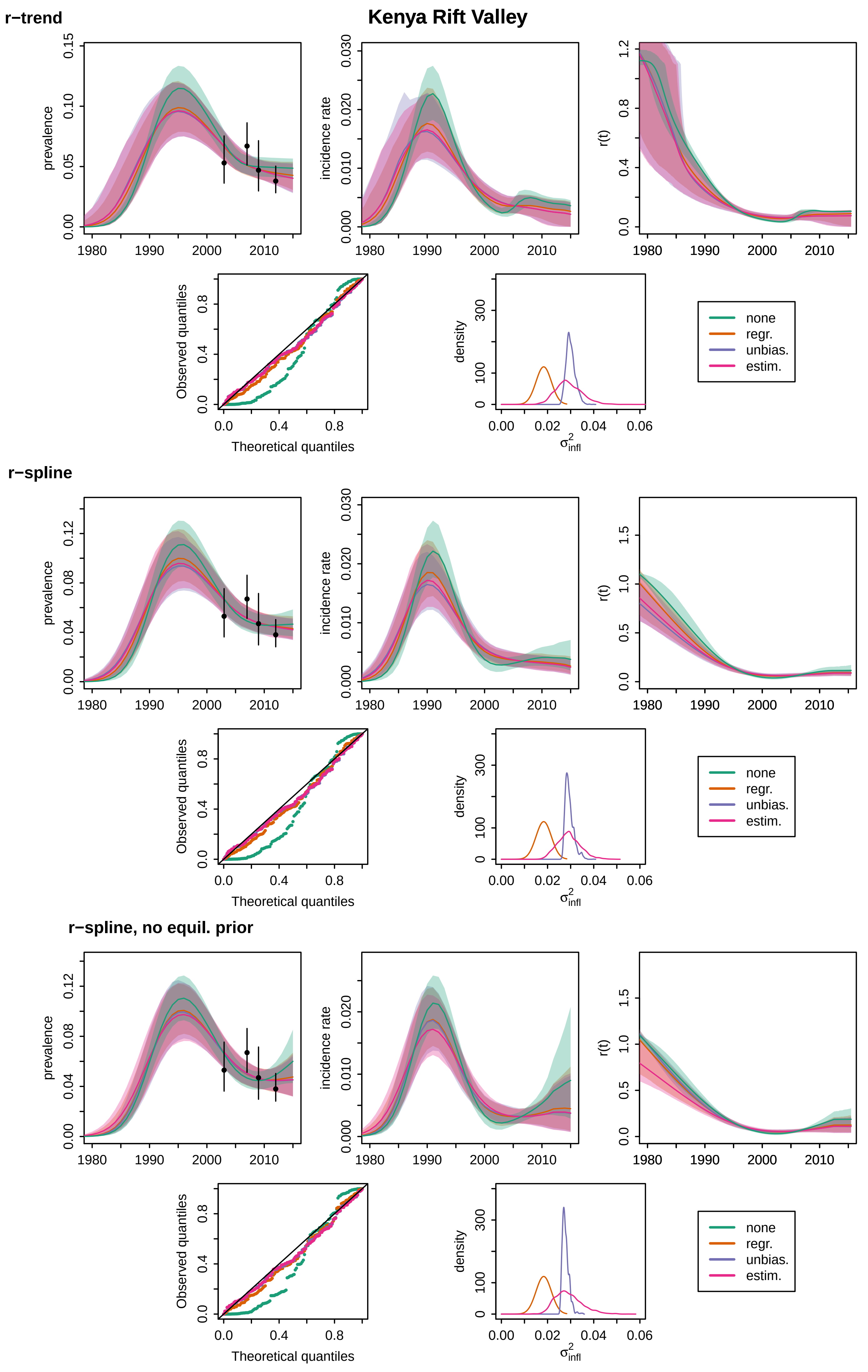

Kenya Rift Valley
r−trend
prevalence
incidence rate
r(t)
Observed quantiles
Theoretical quantiles
density
σ^2_{infl}
none
regr.
unbias.
estim.
r−spline
prevalence
incidence rate
r(t)
Observed quantiles
Theoretical quantiles
density
σ^2_{infl}
none
regr.
unbias.
estim.
r−spline, no equil. prior
prevalence
incidence rate
r(t)
Observed quantiles
Theoretical quantiles
density
σ^2_{infl}
none
regr.
unbias.
estim.

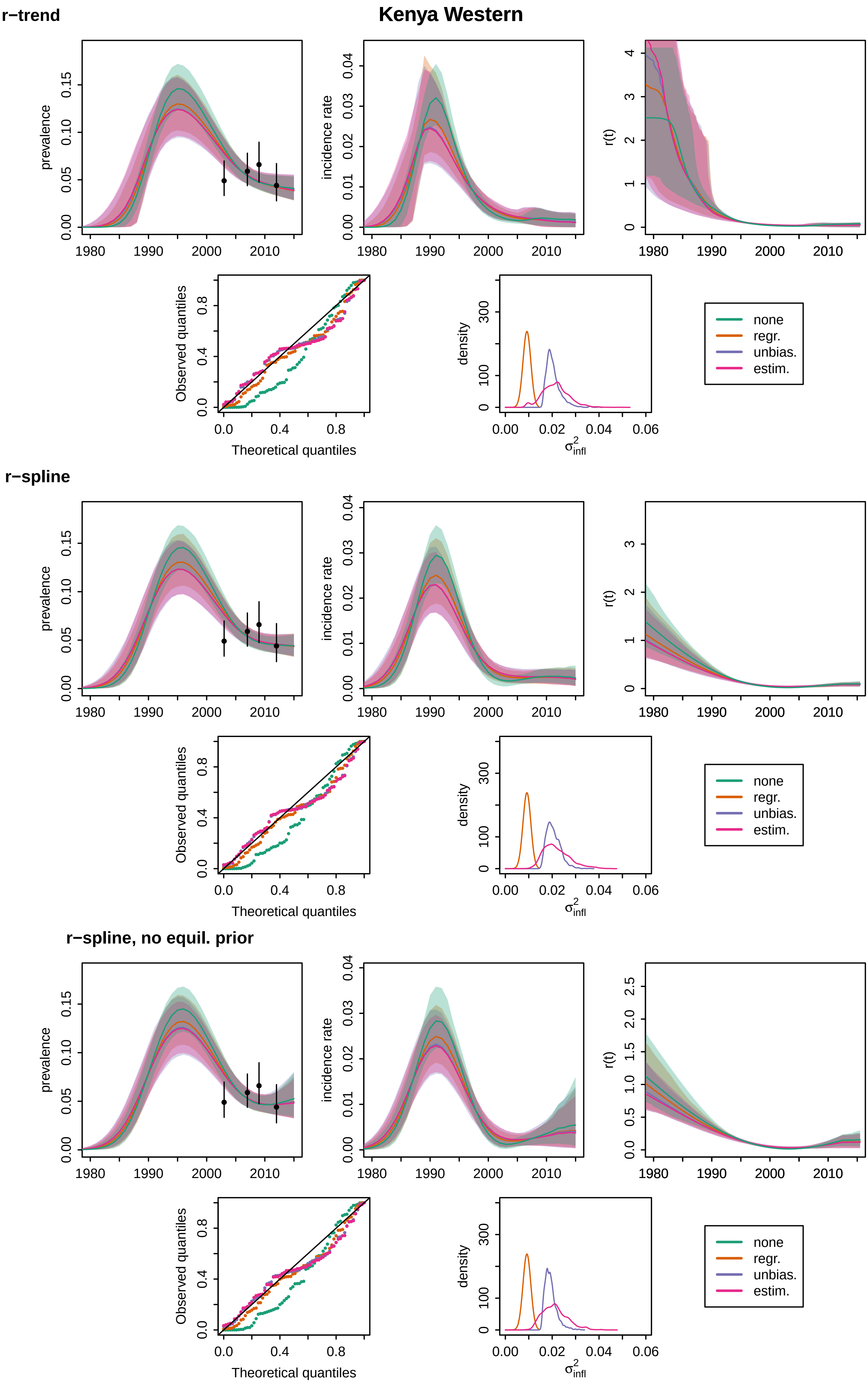
Kenya Western
r−trend
r−spline
r−spline, no equil. prior
prevalence
incidence rate
r(t)
Observed quantiles
Theoretical quantiles
density
σ^2_{infl}
none
regr.
unbias.
estim.
1980
1990
2000
2010
0.00
0.05
0.10
0.15
0.01
0.02
0.03
0.04
0.0
0.4
0.8
0.06
100
300
0.5
1.0
1.5
2.0
2.5
0
1
2
3
4

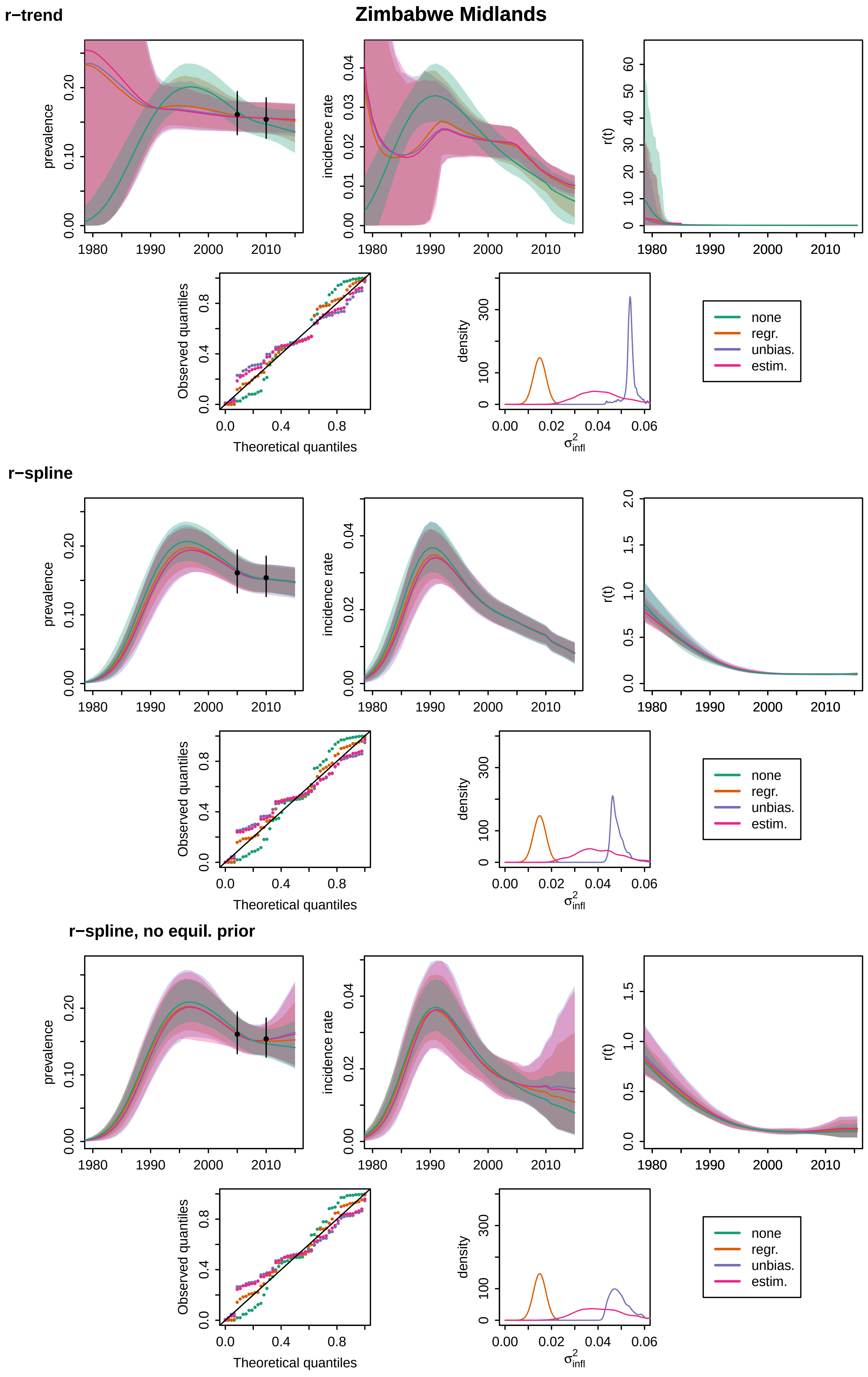
Zimbabwe Midlands
r−trend
r−spline
r−spline, no equil. prior
prevalence
incidence rate
r(t)
Observed quantiles
Theoretical quantiles
density
σ^2_{infl}
none
regr.
unbias.
estim.

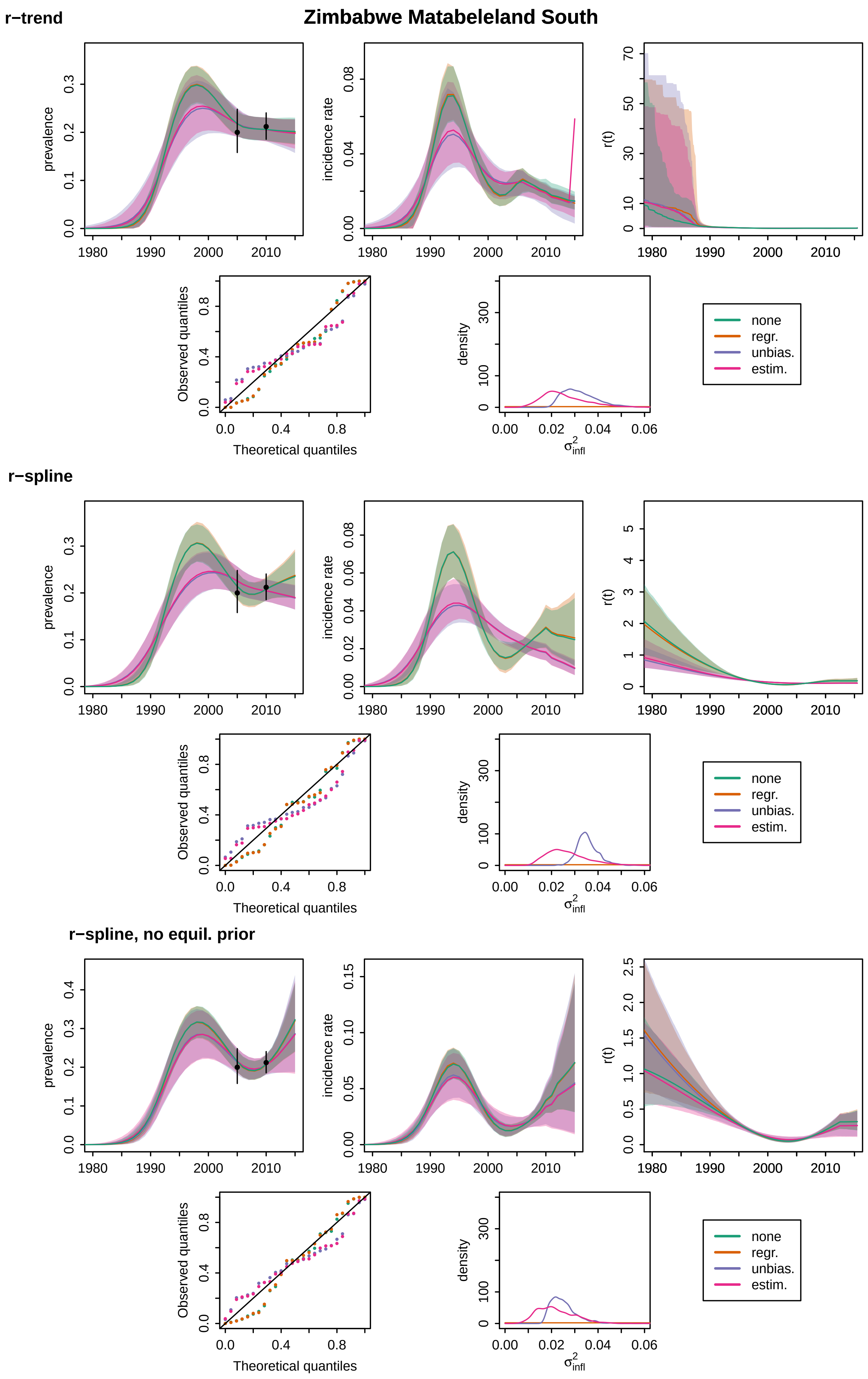
Zimbabwe Matabeleland South
r−trend
r−spline
r−spline, no equil. prior
prevalence
incidence rate
r(t)
Observed quantiles
Theoretical quantiles
density
σ^2_{infl}
none
regr.
unbias.
estim.

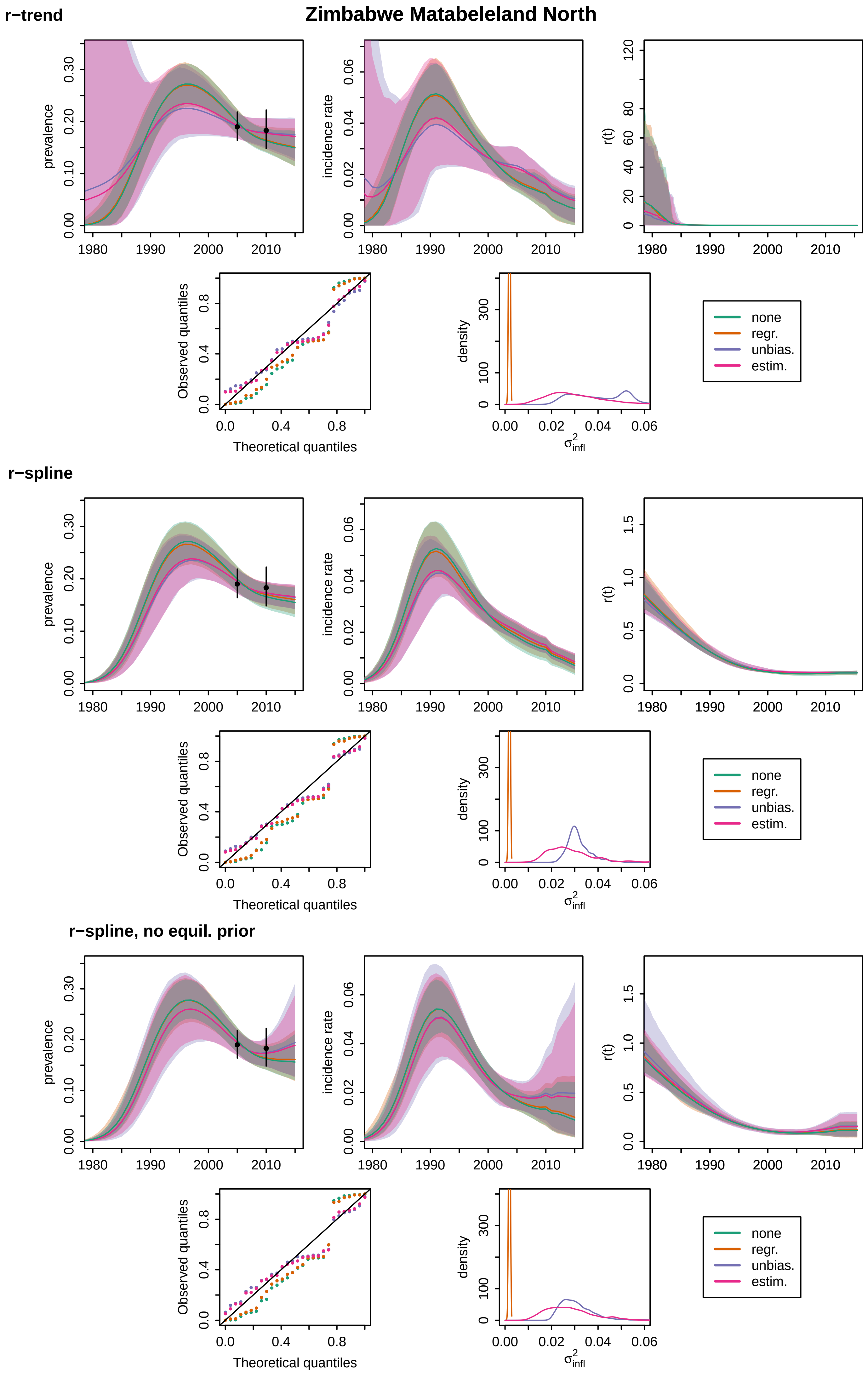
Zimbabwe Matabeleland North
r−trend
r−spline
r−spline, no equil. prior
prevalence
incidence rate
r(t)
Observed quantiles
Theoretical quantiles
density
σ^2_{infl}
none
regr.
unbias.
estim.

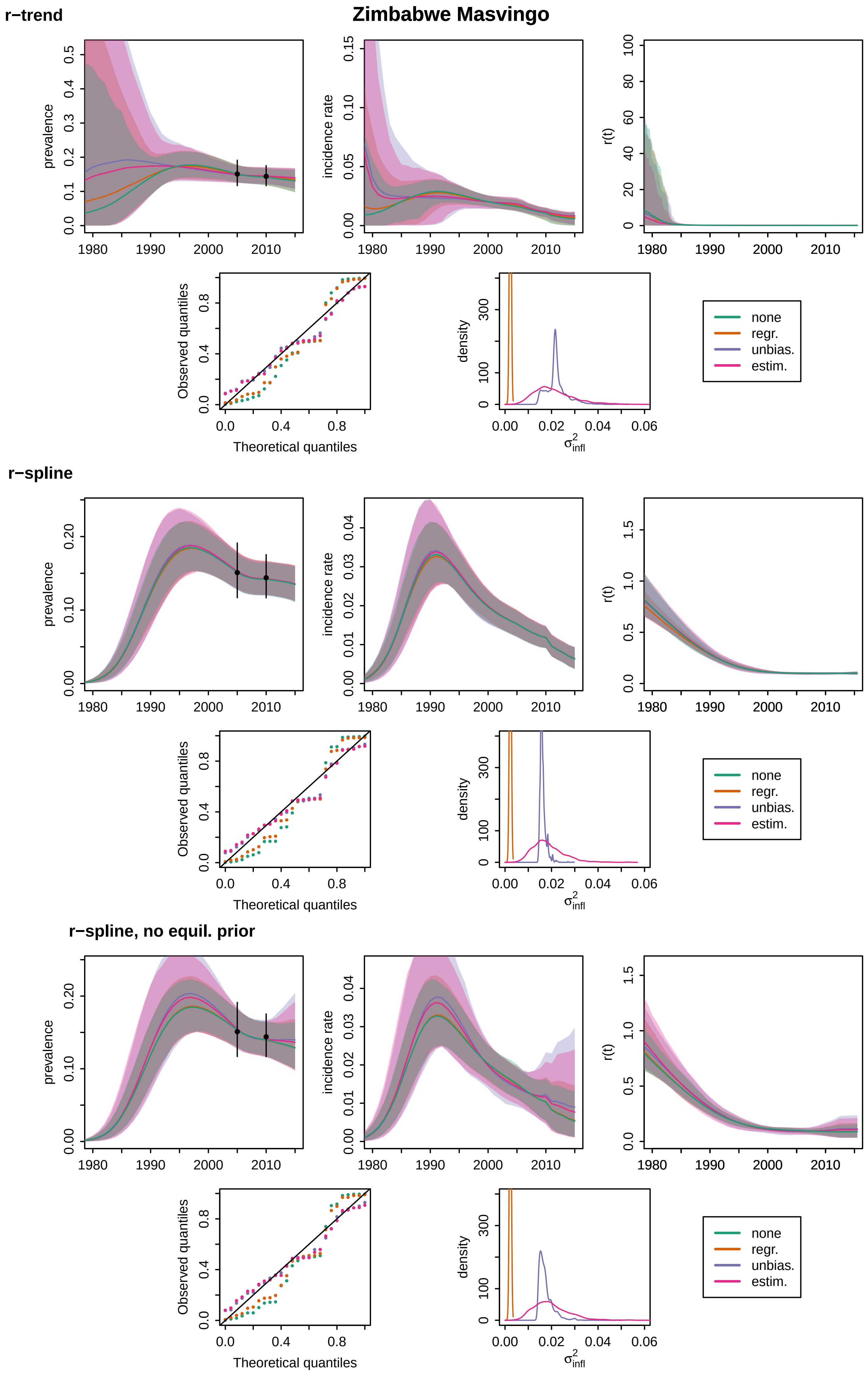
Zimbabwe Masvingo
r−trend
r−spline
r−spline, no equil. prior
prevalence
incidence rate
r(t)
1980
1990
2000
2010
Observed quantiles
Theoretical quantiles
density
σ^2_{infl}
none
regr.
unbias.
estim.

Zimbabwe Mashonaland West

r−trend

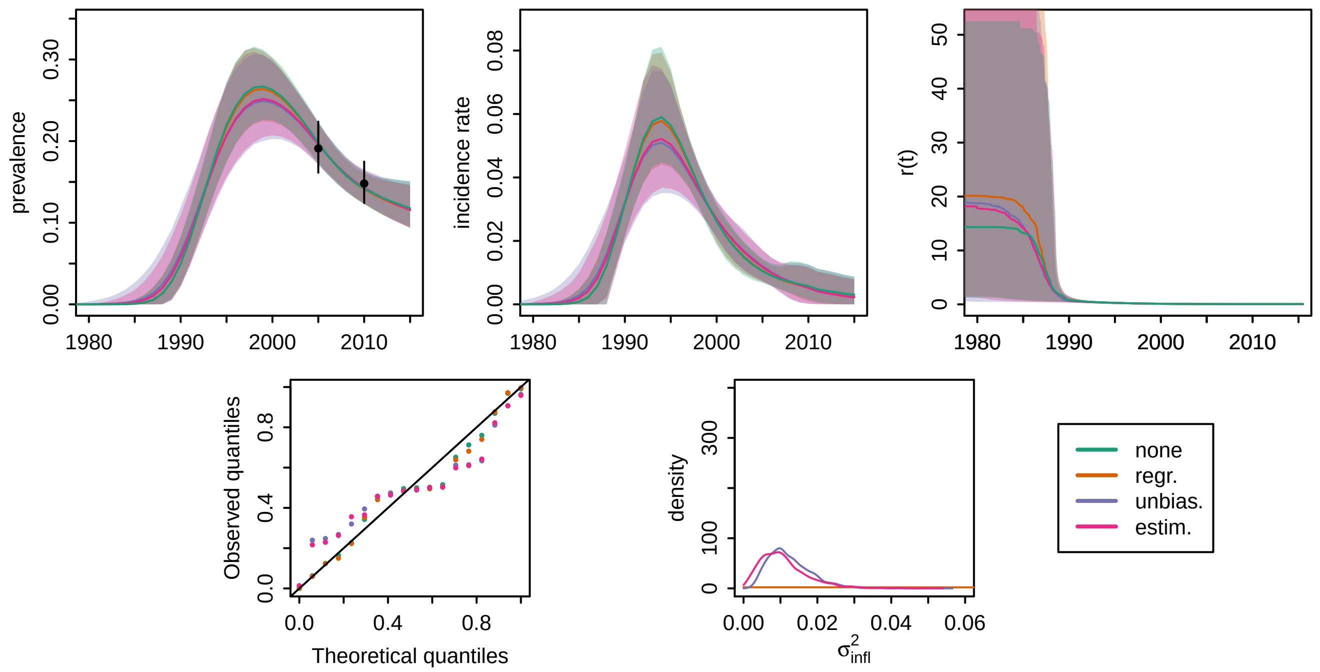

r−spline

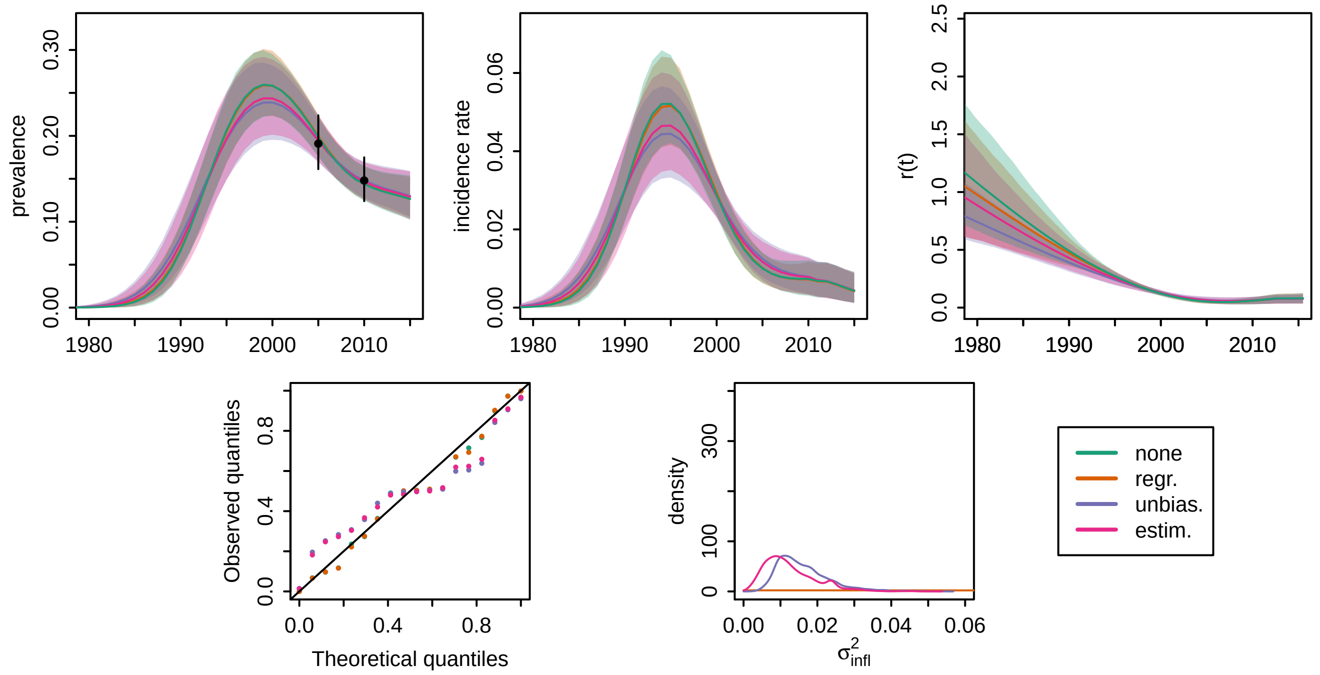

r−spline, no equil. prior

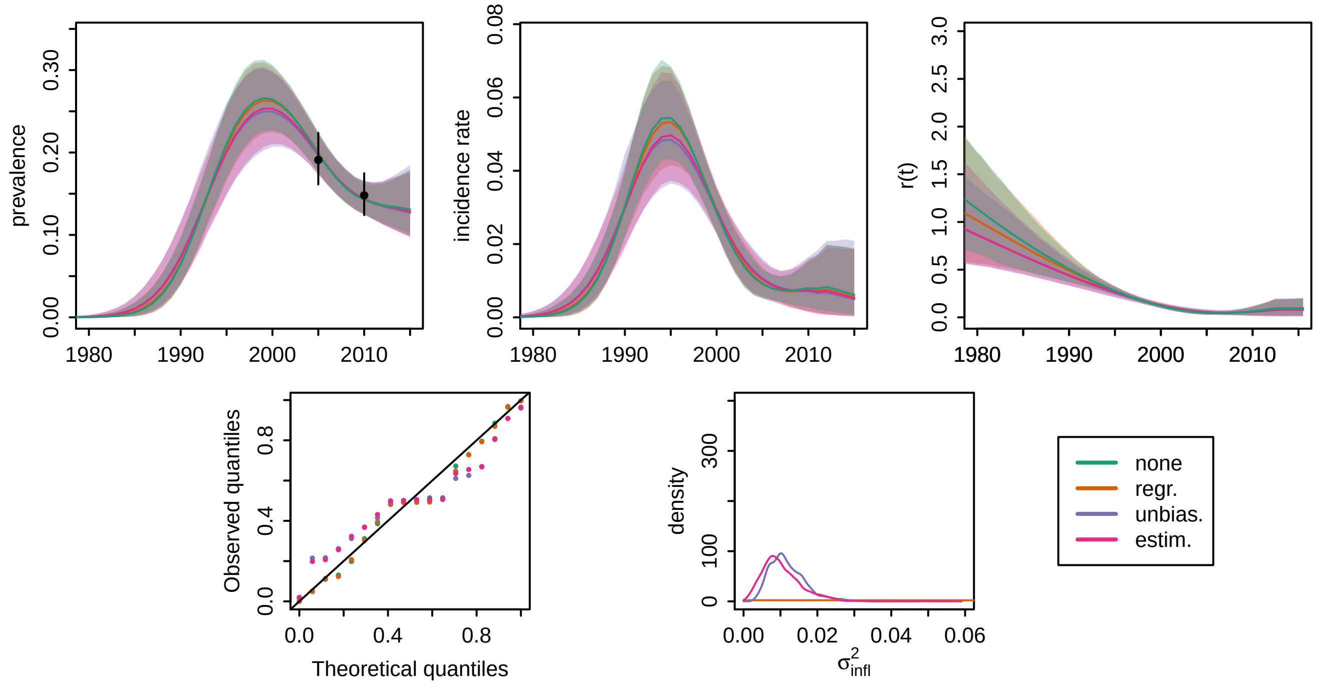

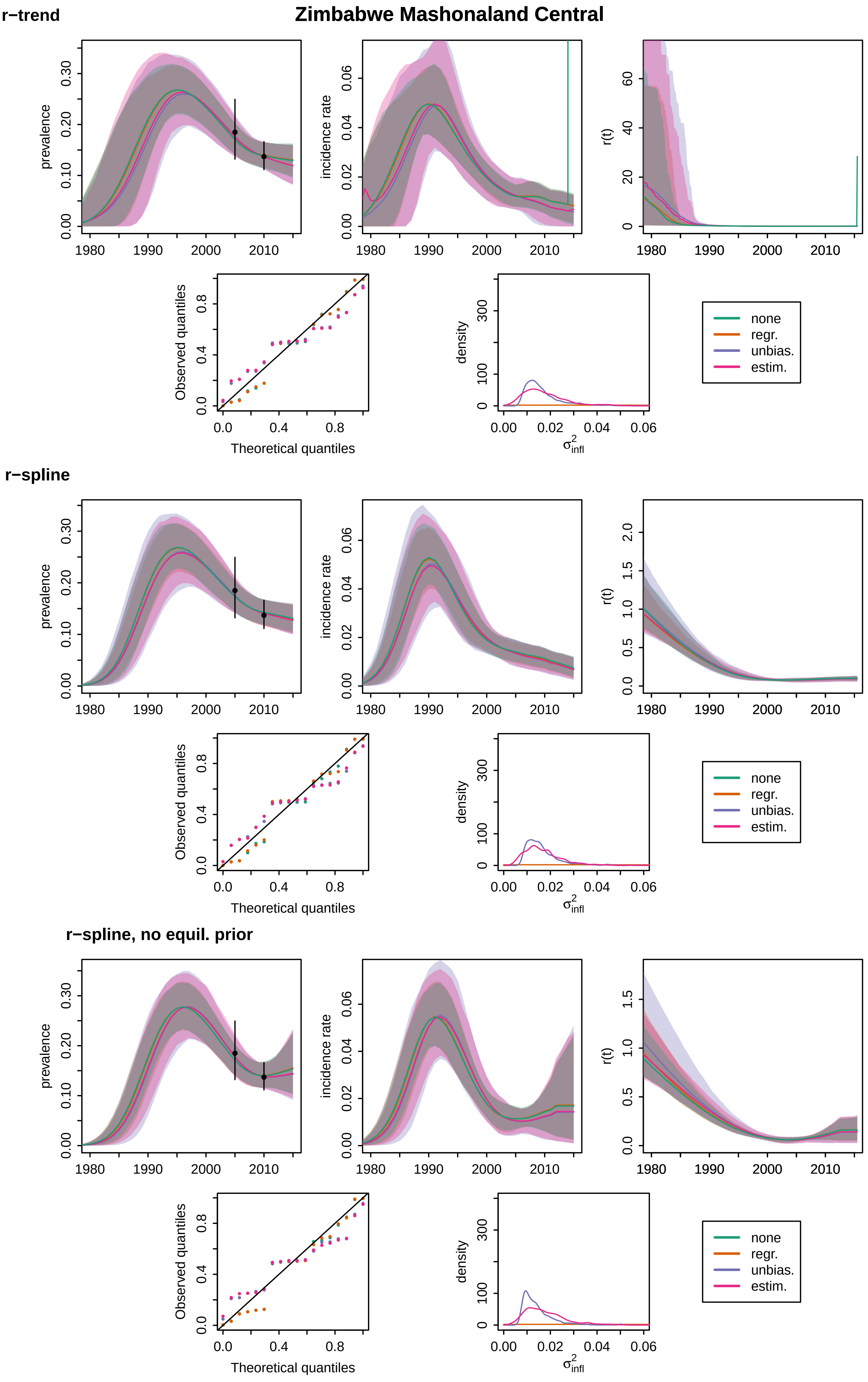
Zimbabwe Mashonaland Central
r−trend
r−spline
r−spline, no equil. prior
prevalence
incidence rate
r(t)
Observed quantiles
Theoretical quantiles
density
σ^2_{infl}
none
regr.
unbias.
estim.

Zimbabwe Manicaland

r−trend

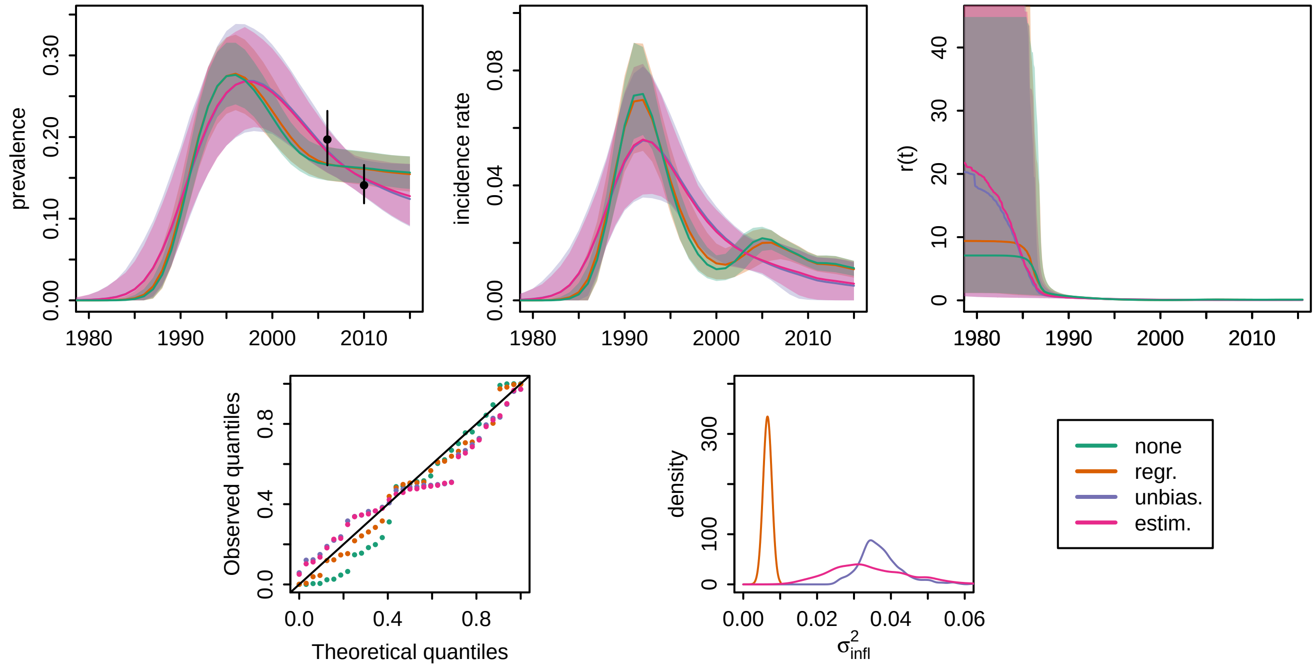

r−spline

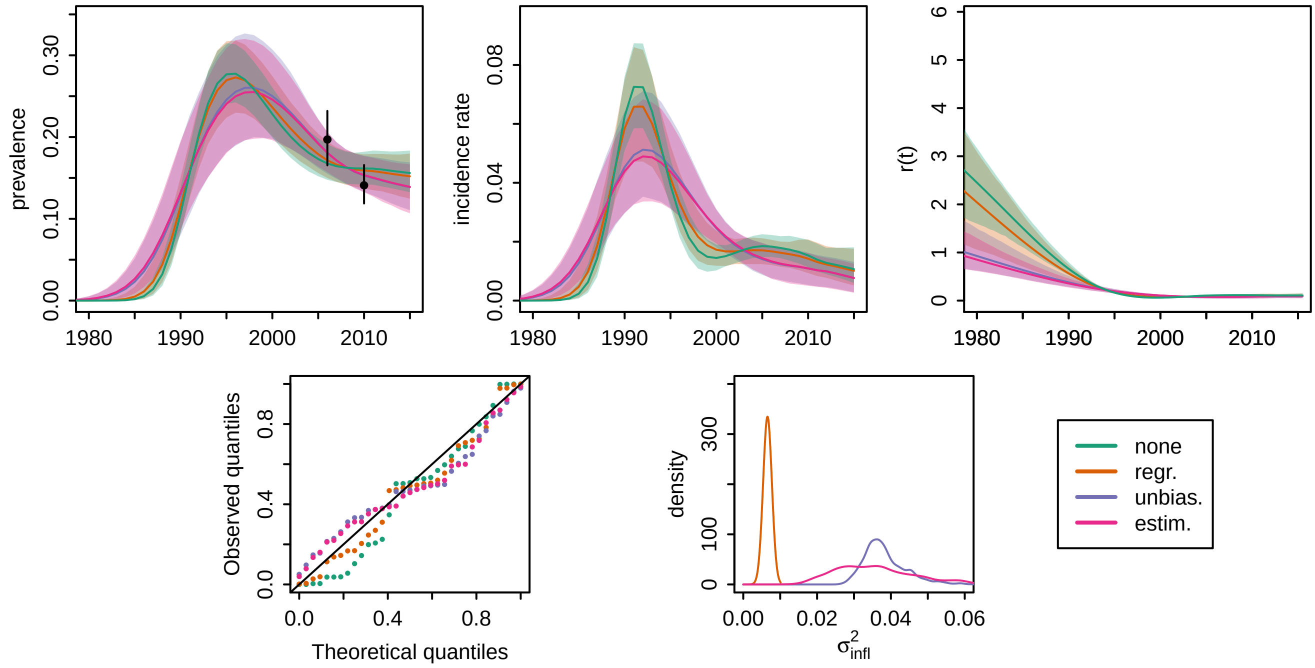

r−spline, no equil. prior

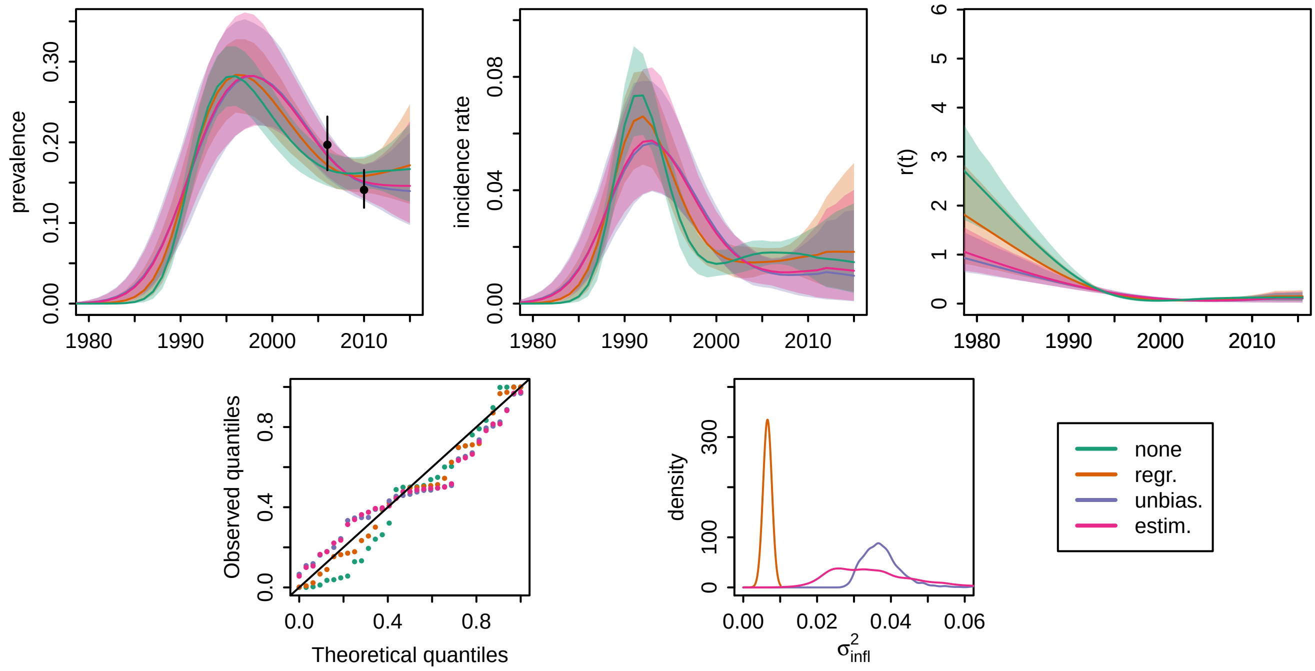

Zimbabwe Harare

r−trend

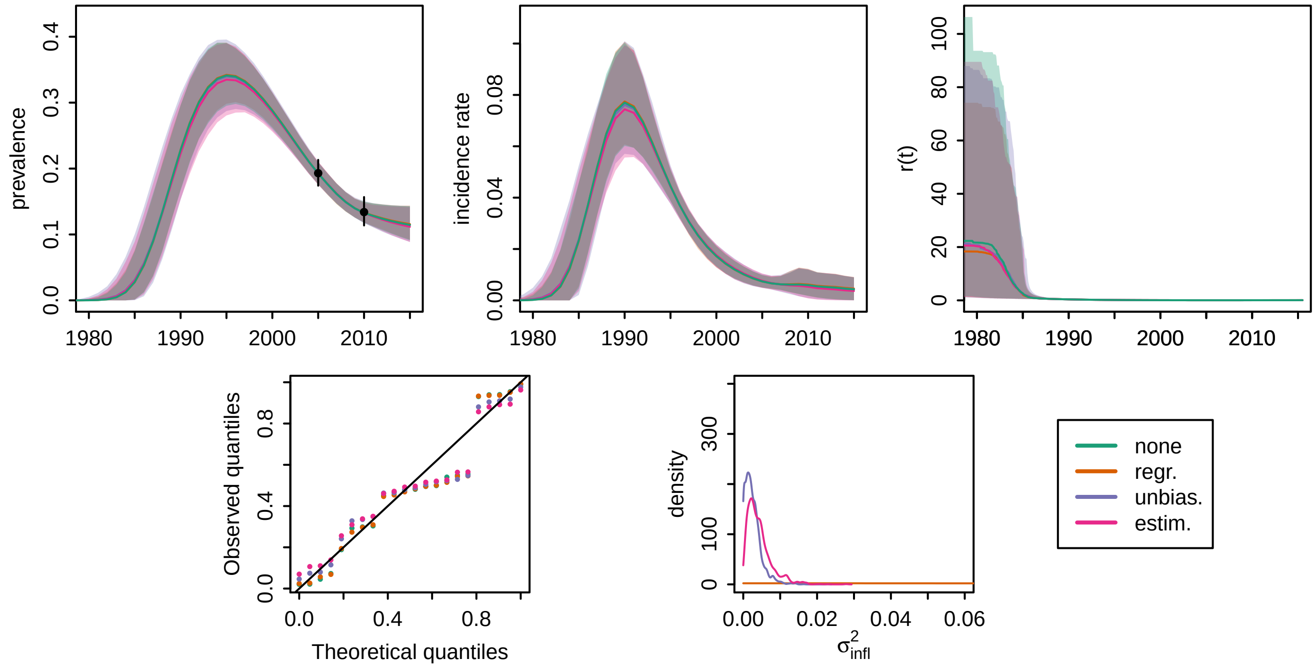

r−spline

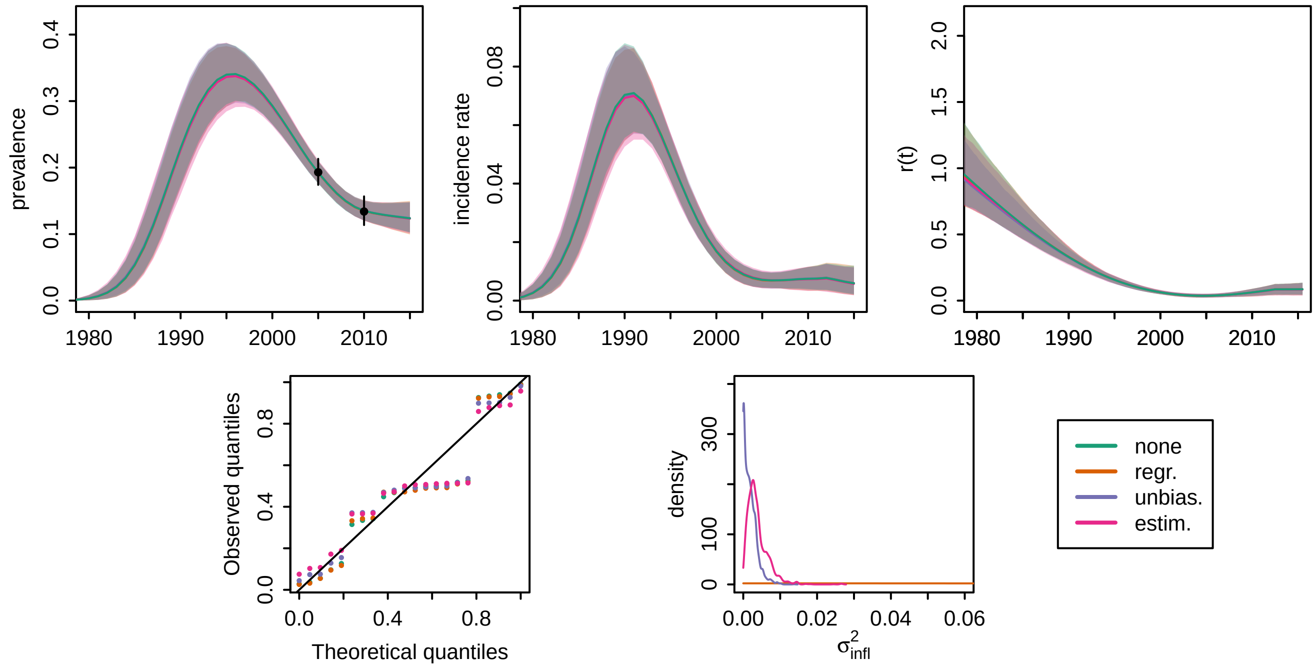

r−spline, no equil. prior

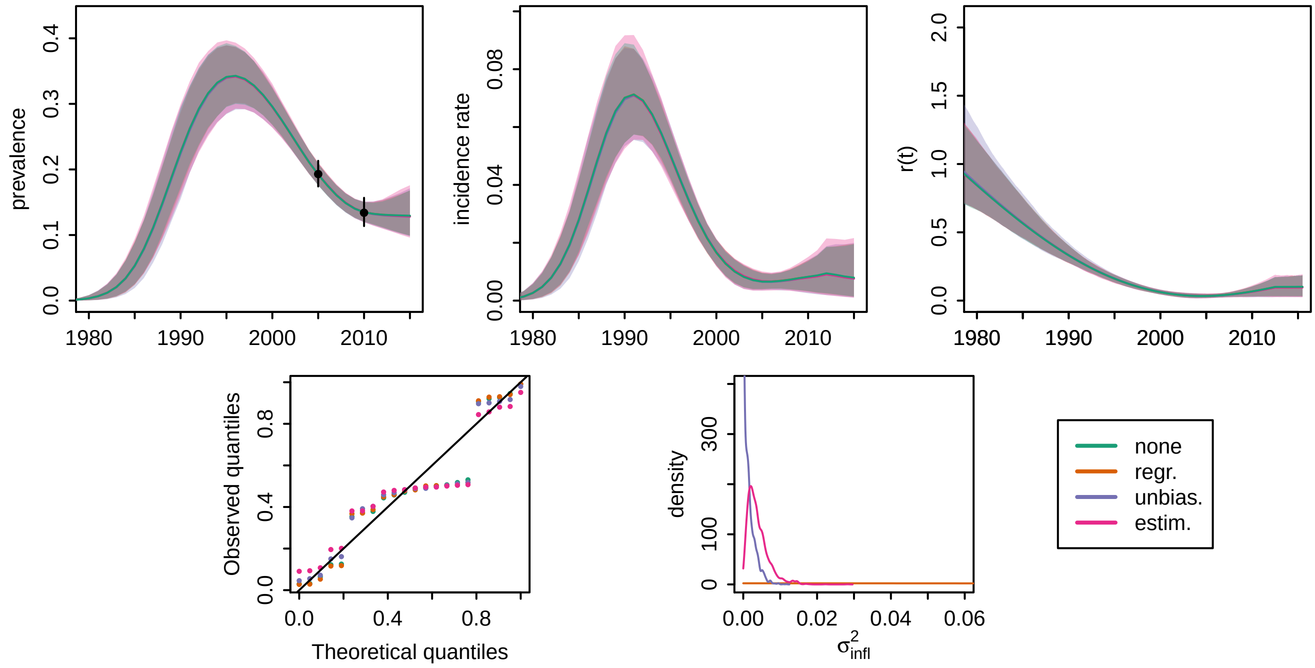

r−trend

Zimbabwe Mashonaland East

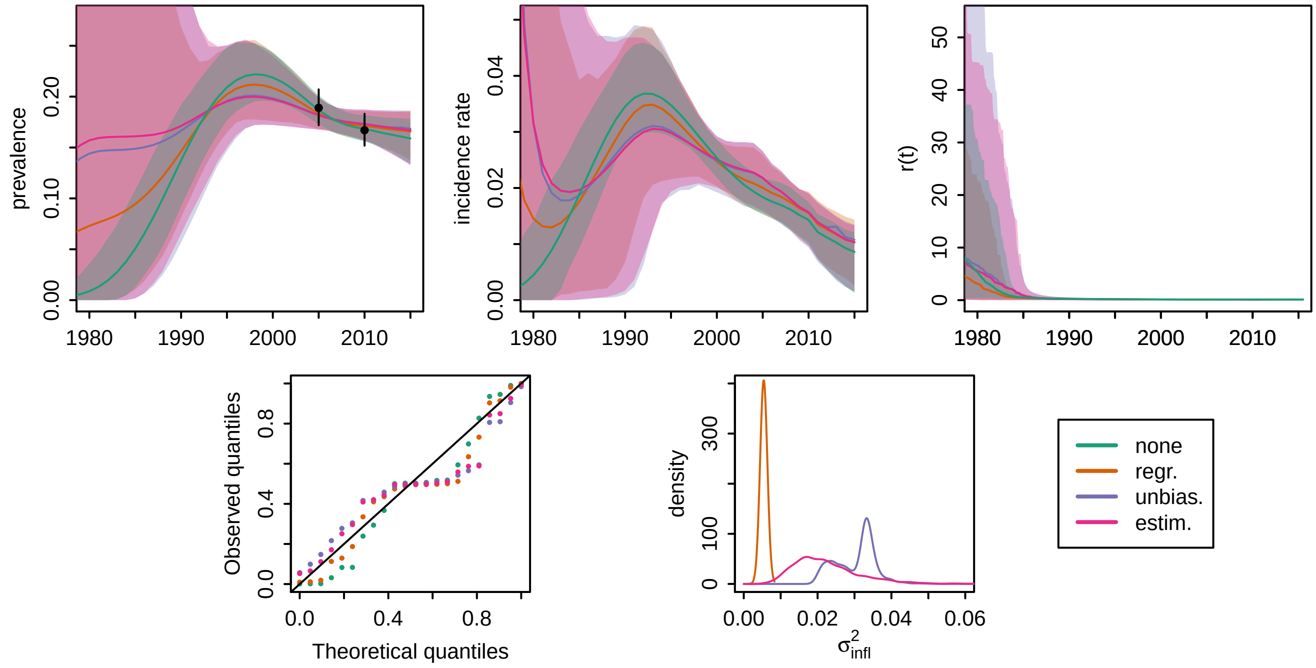

r−spline

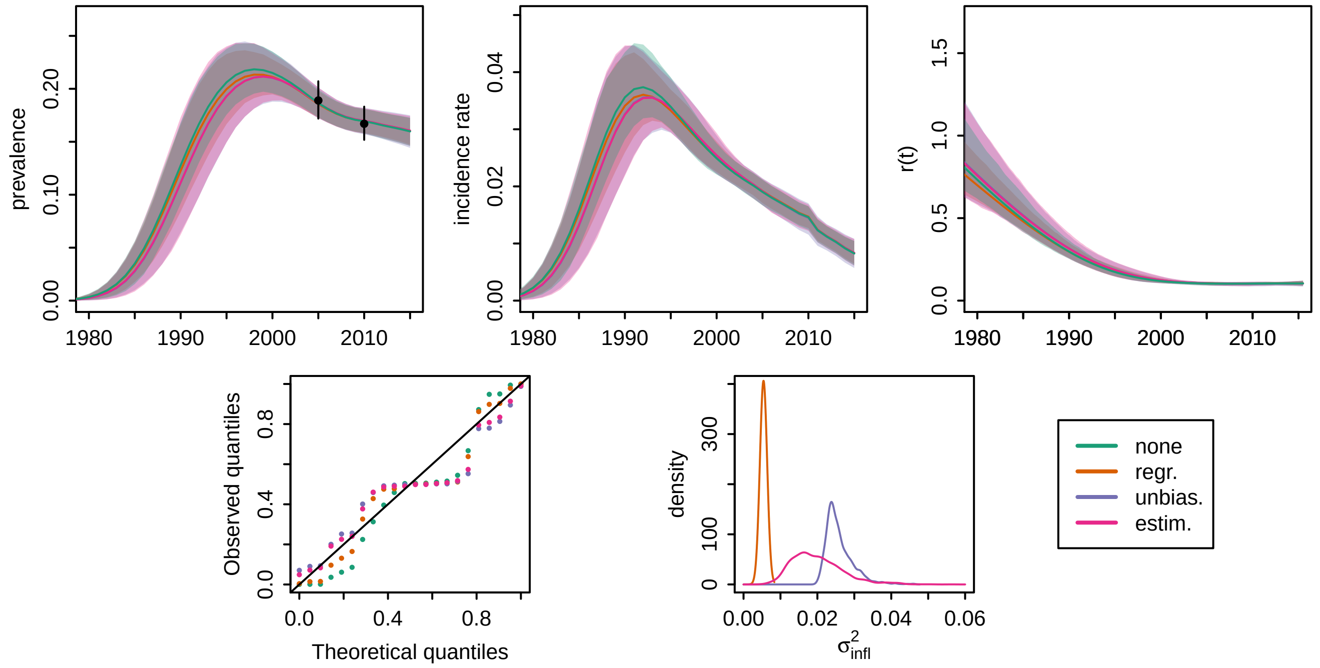

r−spline, no equil. prior

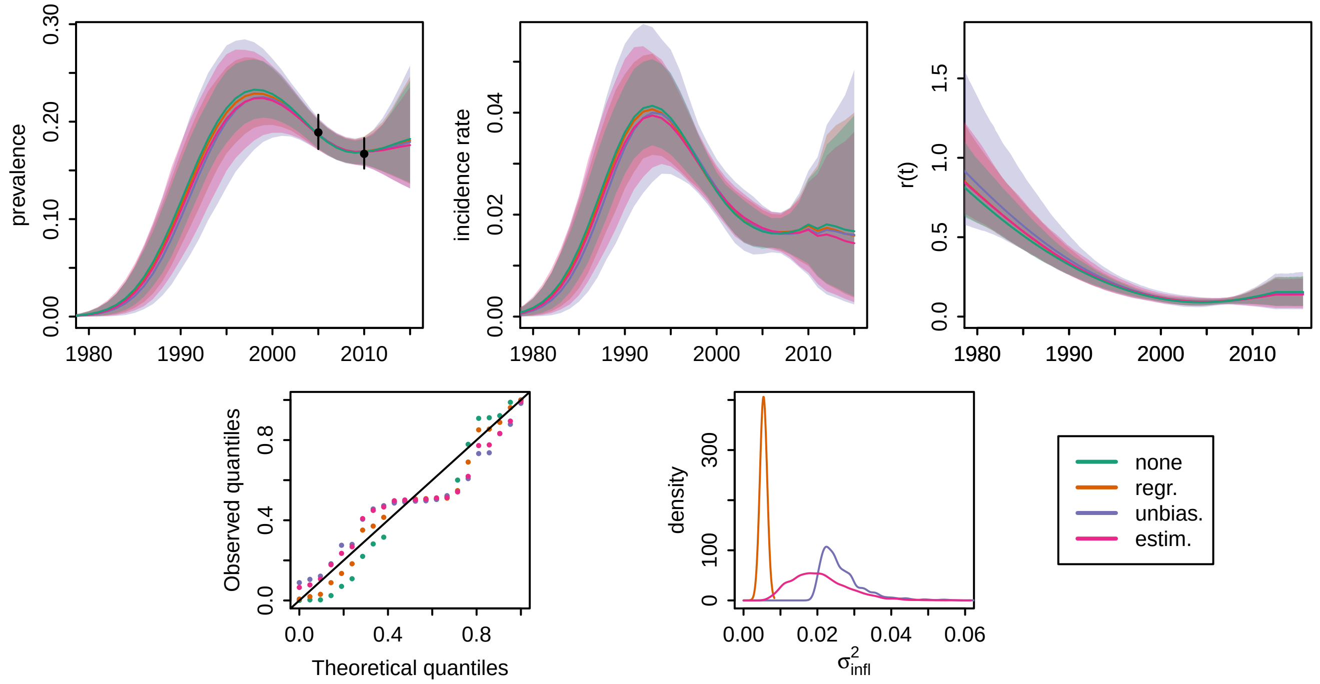

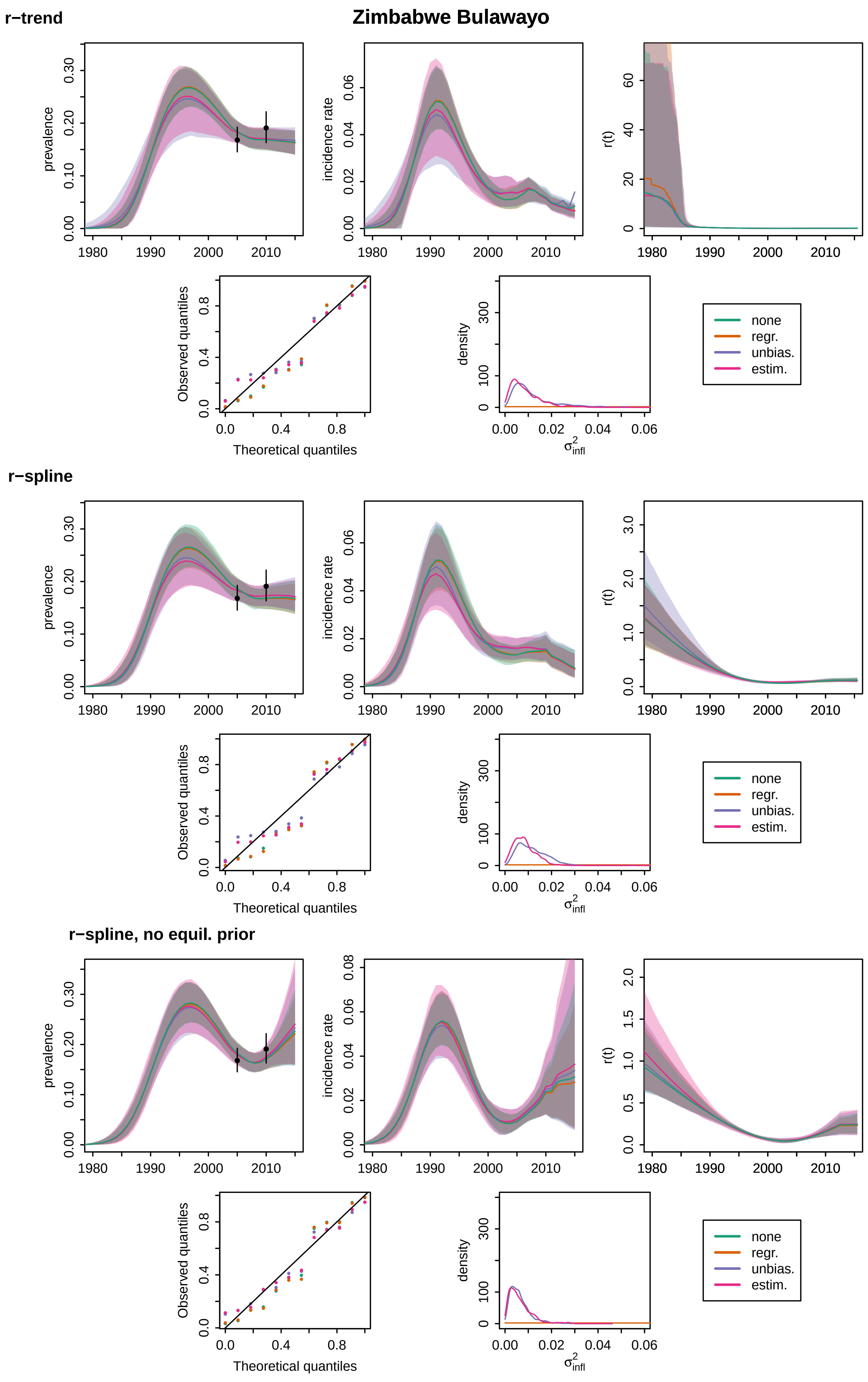

Zimbabwe Bulawayo
r−trend
r−spline
r−spline, no equil. prior
prevalence
incidence rate
r(t)
Observed quantiles
Theoretical quantiles
density
σ^2_{infl}
none
regr.
unbias.
estim.
